# Physiologic signatures within six hours of hospitalization identify acute illness phenotypes

**DOI:** 10.1371/journal.pdig.0000110

**Published:** 2022-10-13

**Authors:** Yuanfang Ren, Tyler J. Loftus, Yanjun Li, Ziyuan Guan, Matthew M. Ruppert, Shounak Datta, Gilbert R. Upchurch, Patrick J. Tighe, Parisa Rashidi, Benjamin Shickel, Tezcan Ozrazgat-Baslanti, Azra Bihorac

**Affiliations:** 1 Intelligent Critical Care Center (IC^3^), University of Florida, Gainesville, Florida, United States of America; 2 Department of Medicine, Division of Nephrology, Hypertension, and Renal Transplantation, University of Florida, Gainesville, Florida, United States of America; 3 Department of Surgery, University of Florida, Gainesville, Florida, United States of America; 4 Department of Computer & Information Science & Engineering, University of Florida, Gainesville, Florida, United States of America; 5 Department of Anesthesiology, University of Florida, Gainesville, Florida, United States of America; 6 J. Crayton Pruitt Family Department of Biomedical Engineering, University of Florida, Gainesville, Florida, United States of America; 7 Sepsis and Critical Illness Research Center, University of Florida, Gainesville, Florida, United States of America; University of Virginia, UNITED STATES

## Abstract

During the early stages of hospital admission, clinicians use limited information to make decisions as patient acuity evolves. We hypothesized that clustering analysis of vital signs measured within six hours of hospital admission would reveal distinct patient phenotypes with unique pathophysiological signatures and clinical outcomes. We created a longitudinal electronic health record dataset for 75,762 adult patient admissions to a tertiary care center in 2014–2016 lasting six hours or longer. Physiotypes were derived via unsupervised machine learning in a training cohort of 41,502 patients applying consensus *k*-means clustering to six vital signs measured within six hours of admission. Reproducibility and correlation with clinical biomarkers and outcomes were assessed in validation cohort of 17,415 patients and testing cohort of 16,845 patients. Training, validation, and testing cohorts had similar age (54–55 years) and sex (55% female), distributions. There were four distinct clusters. Physiotype A had physiologic signals consistent with early vasoplegia, hypothermia, and low-grade inflammation and favorable short-and long-term clinical outcomes despite early, severe illness. Physiotype B exhibited early tachycardia, tachypnea, and hypoxemia followed by the highest incidence of prolonged respiratory insufficiency, sepsis, acute kidney injury, and short- and long-term mortality. Physiotype C had minimal early physiological derangement and favorable clinical outcomes. Physiotype D had the greatest prevalence of chronic cardiovascular and kidney disease, presented with severely elevated blood pressure, and had good short-term outcomes but suffered increased 3-year mortality. Comparing sequential organ failure assessment (SOFA) scores across physiotypes demonstrated that clustering did not simply recapitulate previously established acuity assessments. In a heterogeneous cohort of hospitalized patients, unsupervised machine learning techniques applied to routine, early vital sign data identified physiotypes with unique disease categories and distinct clinical outcomes. This approach has the potential to augment understanding of pathophysiology by distilling thousands of disease states into a few physiological signatures.

## Author summary

In this paper, we present a machine learning approach, consensus clustering, to group hospitalized patients based on six routinely collected vital signs measured within six hours of hospital admission into previously undescribed subsets or acute illness phenotypes that may have different risks for a poor outcome or different treatment responses. We identified four acute illness phenotypes associated with distinct clinical characteristics, biomarker patterns, and clinical outcomes. We validated the reproducibility of phenotypes using different dataset and clustering approach. The early identified phenotypes, that have unique disease states and mortality risk, have the potential to augment understanding of pathophysiology by distilling thousands of disease states into a few physiological signatures and clinical decision-support systems under time constraints.

## Introduction

Each year in the United States alone there are more than 36 million hospital admissions and seven thousand in-hospital mortalities, nearly one quarter of which may be preventable [1–4]. Early in each hospital admission, clinicians formulate decisions regarding diagnostic tests, treatments, and triage destinations using information that has diluted signal-to-noise ratios [5–7]. These arduous clinical decision-making tasks are supported by analyzing vital signs representing essential physiological processes [8–12]. Identifying early vital sign trajectories may have utility for discovering unique physiological signatures that are associated with distinct patient phenotypes and clinical outcomes. Unsupervised machine learning (ML) clustering analyses of clinical variables have identified meaningful subtypes of sepsis and the acute respiratory distress syndrome, but this approach has not been reported among broad, heterogeneous cohorts incorporating all hospitalized patients [13–15].

Using electronic health record data spanning 75,762 adult hospital admissions, we test the hypothesis that unsupervised ML analysis of vital signs recorded within six hours of hospital admission reveals discrete and reproducible physiologic signatures of acute illness phenotypes (*physiotypes*) that are associated with distinct disease categories and clinical outcomes.

## Methods

### Data source and participants

We generated a longitudinal dataset of electronic health records (EHR) for 75,762 hospital admissions of 43,598 patients representing all adults (age ≥18 years) admitted to the University of Florida Health 1000-bed academic hospital between June 1, 2014 and April 1, 2016 with length of stay greater than or equal to six hours including emergency department admission if applicable. Patients completely missing at least two of the six vital sign measurements (systolic and diastolic blood pressure, heart rate, respiratory rate, temperature, and oxygen saturation) within six hours of admission were excluded (S1 Fig). A detailed description of our methods is available in S1 Text. This project was approved by the University of Florida Institutional Review Board.

### Study design

We followed Transparent Reporting of a multivariable prediction model for Individual Prognosis Or Diagnosis (TRIPOD) recommendations under the Type 2b analysis category [16] to chronologically split the dataset into training (admissions between June 1, 2014 and May 31, 2015, n = 41,502), validation (admissions between June 1, 2015 and October 31, 2015, n = 17,415), and testing (admissions between November 1, 2015 and April 1, 2016, n = 16,845) cohorts to mitigate potentially adverse effects of dataset drift due to changes in clinical practice or patient populations. To identify acute illness phenotypes (*physiotypes*) using early physiologic signatures, we applied unsupervised ML clustering to temporal measurements of six vital signs recorded within six hours of hospital admission in the training cohort. We assessed *physiotype* reproducibility by applying alternative clustering methods in the training dataset, assessing *physiotype* frequency distributions and clinical outcomes in the validation cohort, and predicting *physiotypes* in the testing cohort (S2 Fig).

### Identifying acute illness *physiotypes* using early physiologic signatures

To derive *physiotypes* with reproducible early physiologic signatures, we applied consensus *k*-means clustering [17] to 36 features derived from time series of six vital signs measured within six hours of hospital admission for each encounter in the training cohort. Based on consensus matrix plots and cumulative distribution function curves, the optimal number of physiologic clusters was four (S3 Fig) [15].

We processed raw time series to remove outliers and assess distributions, missingness, and correlation (S1 Table and S4 Fig). Raw time series were resampled to an hourly frequency, using mean values when multiple measurements were recorded during the same one-hour window. Missing values were imputed by forward and backpropagating temporally adjacent values [18]. For records with no measurements within six hours of hospitalization, we imputed median values from the training cohort. Each admission was represented by six hourly values for six vital signs, yielding 36 clustering features. Vital sign patterns were visualized using line plots with 95% confidence intervals, t-distribution stochastic neighbor embedding (t-SNE) plots, ranked plots for mean standardized difference between *physiotype* pairs, and vital sign mosaic plots (see S1 Text for a comprehensive description).

### Clinical characteristics, biological correlates, and clinical outcomes

For each admission we extracted demographics, 19 clinical biomarkers routinely measured at hospital admission (S2 Table), Sequential Organ Dysfunction Assessment (SOFA) and Modified Early Warning Score (MEWS) acuity scores, and patient outcomes [19,20]. Details on data processing are described in S1 Text. Primary outcomes were thirty-day and three-year mortality. Median follow-up duration was 4.3 years per reverse Kaplan-Meier method. Other outcomes were acute kidney injury (AKI), venous thromboembolism, sepsis, intensive care unit (ICU) admission, mechanical ventilation (MV), and renal replacement therapy (RRT).

### Statistical methods

We assessed *physiotype* reproducibility by comparing phenotype derivation with gaussian mixture modeling (GMM) [21] in the training dataset and by assessing frequency distributions in the validation and testing cohorts (S2 Fig). We assessed the robustness of derived *physiotypes* using sensitivity analyses excluding variables with high missingness, excluding both highly missing and highly correlated variables, and using a 12-hour vital sign window. We validated derived *physiotypes* in two steps. In the validation cohort we rederived clusters using consensus *k*-means and compared them with training cohort clusters. In the testing cohort, we predicted *physiotypes* based on the clinical characteristics of training cohort clusters. Predictions arose from the minimum Euclidean distance from each patient to the centroid of each *physiotype* (S1 Text). Clinical variables across clusters were compared using line plots, t-distribution stochastic neighbor embedding plots, and ranked plots.

*Physiotypes* were compared using the χ2 test for categorical variables and analysis of variance and the Kruskal-Wallis test for continuous variables. Overall survival was illustrated using Kaplan–Meier curves and compared using the log-rank test. Adjusted hazard ratios (HR) for each *physiotype* were compared using Cox proportional-hazards regression while adjusting for age, sex, comorbidities, and SOFA score on admission. We adjusted *p* values for the family-wise error rate due to multiple comparisons using the Bonferroni correction. To assure that *physiotypes* did not recapitulate existing acuity scores, we compared *physiotypes* with SOFA scores within 24 hours of admission using alluvial plots and chord diagrams. Analyses were performed with Python version 3.7 and R version 3.5.1.

## Results

### Clinical characteristics of patients

Training, validation, and testing cohorts had similar clinical characteristics, biomarker distributions, and outcomes (S3 and S4 Tables). Average patient age was 54 years and sex was equally distributed. Almost two thirds of all patients had urgent admissions, 18% were transferred from another hospital, 27% were admitted to an ICU or intermediate care unit (IMC), and 28% had surgery during admission. Among patients admitted directly to an ICU/IMC, 22–27% had high acuity scores (SOFA greater than 6 or MEWS greater than 4) on admission. Among patients admitted to hospital wards, 2–3% had high acuity scores. Overall thirty-day and three-year mortality rates were 4% and 19%, respectively.

### Derivation and characteristics of *physiotypes*

**Fig 1:**
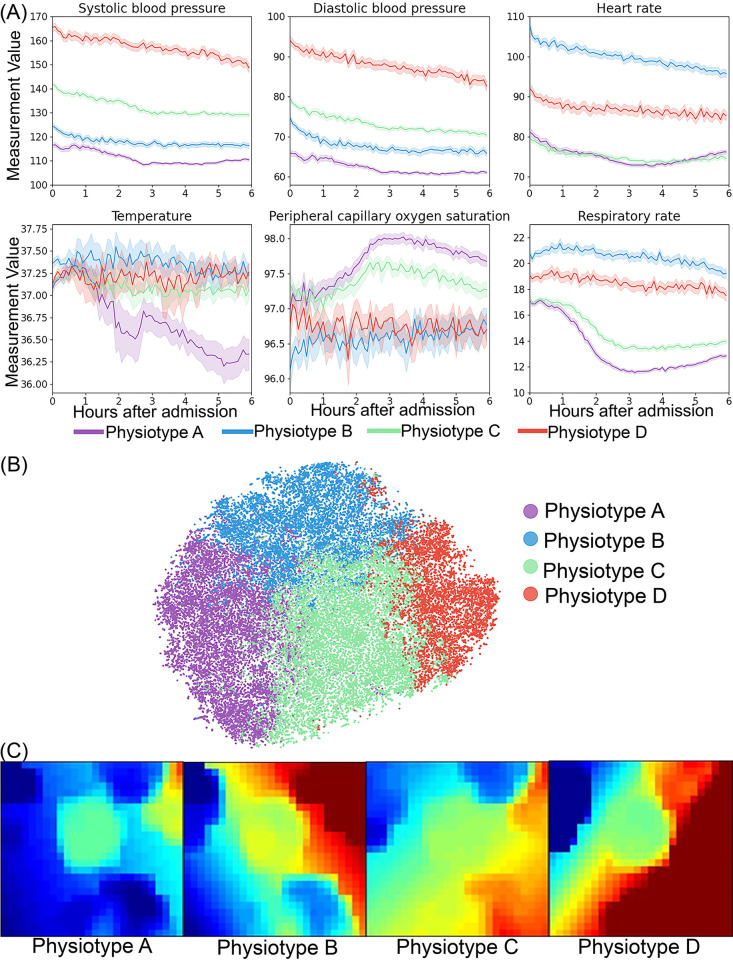
Physiotype vital sign representations. (A) Distribution of vital signs during the first six hours of hospital admission. (B) Visualization of physiotypes using the t-distributed stochastic neighbor embedding (t-SNE) technique. (C) Physiotype average vital sign mosaics using a self-organizing map.

We identified four *physiotypes* with unique pathophysiological signatures, disease categories, and clinical outcomes (Tables 1 and 2, S5 and S6 Tables, Fig 1). *Physiotypes* were labeled as Physiotype A (31% of total cohort), B (23% of total cohort), C (31% of total cohort), and D (15% of total cohort) according to ascending value of systolic blood pressure (Fig 1A).

**Table 1:** Physiotype clinical characteristics and biomarkers.

Variables	Total	Acute Illness Physiotypes			
		Physiotype A	Physiotype B	Physiotype C	Physiotype D
Number of Encounters (%)	41,502	12,695 (31)	9,710 (23)	12,962 (31)	6,135 (15)
**Preadmission clinical characteristics**					
Age, mean (SD), years	54 (19)	53 (18)^a^	50 (20)^a^	56 (18)	56 (17)
Female sex, n (%)	22,745 (55)	7,291 (57)^a^	5,585 (58)^a^	6,641 (51)	3,228 (53)
Race, n (%)					
White	29,076 (70)	9,577 (75)^a^	6,723 (69)^a^	9,195 (71)	3,581 (58)^a^
African American	9,634 (23)	2,090 (16)^a^	2,342 (24)	2,947 (23)	2,255 (37)^a^
Primary Insurance, n (%)					
Private	9,591 (23)	3,158 (25)^a^	2,278 (23)	2,991 (23)	1,164 (19)^a^
Medicare	18,499 (45)	5,604 (44)^a^	3,852 (40)^a^	6,124 (47)	2,919 (48)
Medicaid	9,231 (22)	2,767 (22)^a^	2,588 (27)^a^	2,566 (20)	1,310 (21)
Uninsured	4,181 (10)	1,166 (9)	992 (10)	1,281 (10)	742 (12)^a^
Residing neighborhood characteristics					
Proportion of African-Americans (%), mean (SD)	18.7 (17.5)	17.3 (16.1)^a^	19.3 (17.8)^a^	18.5 (17.3)	21.3 (19.4)^a^
Proportion Below Poverty (%), mean (SD)	22.7 (10.1)	21.8 (10.0)^a^	23.2 (9.9)^a^	22.6 (10.0)	24.0 (10.4)^a^
Distance from Hospital (mile), median (IQR)	18 (3, 34)	22 (3, 37)^a^	14 (3, 32)^a^	18 (3, 34)	14 (3, 27)^a^
**Comorbidities**					
Hypertension, n (%)	21,639 (52)	6,498 (51)	5,000 (51)	6,723 (52)	3,418 (56)^a^
Cardiovascular disease, n (%)^b^	12,058 (29)	3,477 (27)^a^	2,833 (29)	3,783 (29)	1,965 (32)^a^
Diabetes mellitus, n (%)	10,111 (24)	2,934 (23)	2,400 (25)	3,125 (24)	1,652 (27)^a^
Chronic kidney disease, n (%)	6,518 (16)	1,757 (14)^a^	1,454 (15)	2,056 (16)	1,251 (20)^a^
**Admission characteristics of patients**					
Emergent Admission, n (%)	30,177 (73)	7,367 (58)^a^	8,106 (83)^a^	9,244 (71)	5,460 (89)^a^
Transfer from another hospital, n (%)	7,115 (17)	1,943 (15)	1,957 (20)^a^	2,100 (16)	1,115 (18)^a^
**Primary admission diagnostic groups**					
Diseases of the circulatory system	7,719 (19)	2,142 (17)^a^	1,503 (15)^**a**^	2533 (20)	1,541 (25)^a^
Respiratory and infectious diseases	3,306 (8)	571 (4)^a^	1,403 (14)^a^	692 (5)	640 (10)^**a**^
Complications of pregnancy and childbirth	3,148 (8)	857 (7)	1,100 (11)^a^	862 (7)	329 (5)^**a**^
Diseases of the digestive/genitourinary systems	5,184 (12)	1,857 (15)^a^	1,028 (11)^a^	1661 (13)	638 (10)^a^
Diseases of the musculoskeletal/connective tissue and skin	3,651 (9)	1,489 (12)^a^	479 (5)^a^	1216 (9)	467 (8)^a^
Neoplasms	2,743 (7)	1,244 (10)^a^	377 (4)^a^	950 (7)	172 (3)^a^
**Clinical biomarkers and interventions within 24 hours of admission**					
Surgical procedure on admission day, n (%)	8,644 (21)	4,441 (35)^a^	796 (8)^a^	2933 (23)	474 (8)^a^
ICU/IMC admission within first 24 hours, n (%)	9,426 (23)	2,893 (23)^a^	3,022 (31)^a^	2151 (17)	1,360 (22)^a^
**Cardiovascular system**					
Hypotension (MAP < 60 mmHg) at any time, n (%)	14,470 (35)	7,420 (58)^a^	3,393 (35)^a^	3051 (24)	606 (10)^a^
Duration, median (IQR), minutes	57 (15, 168)	60 (18, 197)^a^	75 (30, 212)^a^	18 (6, 62)	24 (8, 68)
Vasopressors used, n (%)	7,531 (18)	4,079 (32)^a^	995 (10)^a^	2113 (16)	344 (6)^a^
Out of operating room	1,403 (3)	646 (5)^a^	494 (5)^a^	198 (2)	65 (1)
Hypertension (SBP > 160 mmHg) at any time, n (%)	14,838 (36)	2,742 (22)^a^	1,611 (17)^a^	5629 (43)	4,856 (79)^a^
Troponin, tested, n (%)	14,616 (35)	3,223 (25)^a^	4,090 (42)^a^	4214 (33)	3,089 (50)^a^
Abnormal result among tested, n (%)	3,398 (23)	791 (25)^a^	987 (24)^a^	816 (19)	804 (26)^a^
**Respiratory system**					
Highest administered FiO2, median (IQR), %	0.21 (0.21, 0.40)	0.28 (0.21, 0.40)^a^	0.21 (0.21, 0.33)^a^	0.21 (0.21, 0.40)	0.21 (0.21, 0.29)^a^
Room air only, n (%)	23,963 (58)	6,273 (49)^a^	5,580 (57)^a^	8040 (62)	4,070 (66)^a^
0.22 – 0.40, n (%)	14,790 (36)	5,419 (43)^a^	3,285 (34)	4320 (33)	1,766 (29)^a^
> 0.40, n (%)	2,749 (7)	1,003 (8)^a^	845 (8)^a^	602 (4)	299 (5)
P_a_O2/FiO_2_, tested with arterial blood gas, n (%)	6,113 (15)	2,015 (16)^a^	1,965 (20)^a^	1,345 (10)	788 (13)^a^
<200 among tested, n (%)	2,265 (5)	747 (37)^a^	837 (43)^a^	427 (32)	254 (32)
Mechanical ventilation, n (%)	2,123 (5)	808 (6)^a^	656 (7)^a^	449 (3)	210 (3)
**Kidney and acid-base status**					
Preadmission estimated glomerular filtration rate^c^ (mL/min per 1.73 m^2^), median (IQR)	95 (78, 111)	96 (80, 112)^a^	100 (83, 117)^a^	93 (77, 107)	90 (59, 105)^a^
Highest /reference creatinine^c^ ratio, mean (SD)	1.24 (0.66)	1.25 (0.71)^a^	1.31 (0.73)^a^	1.18 (0.54)	1.24 (0.67)^a^
Renal Replacement therapy, n (%)	641 (2)	170 (1)	119 (1)	128 (1)	224 (4)^a^
Highest Anion Gap, median (IQR), mmol/L	14 (12, 17)	13 (11, 16)	15 (12, 18)^a^	14 (11, 16)	15 (12, 17)^a^
Arterial Blood Gas tested, n (%)	6,115 (15)	2,016 (16)^a^	1,966 (20)^a^	1345 (10)	788 (13)^a^
pH < 7.3 among tested, n (%)	1,437 (23)	532(26)^a^	558 (28) ^a^	216 (16)	131 (17)
Highest Base deficit among tested, mean (SD), mmol/L	4.8 (4.7)	4.4 (4.2)^a^	6.4 (5.8)^a^	3.6 (3.2)	4.3 (3.7)^a^
Lactate, tested, n (%)	15,447 (37)	4,360 (34)^a^	4,660 (48)^a^	4,006 (31)	2421 (39)^a^
2 – 4 mmol/L among tested, n (%)	3,739 (24)	1,012 (23)	1,305 (28)^a^	854 (21)	568 (23)
> 4 mmol/L among tested, n (%)	1,374 (9)	379 (9)^a^	607 (13)^a^	204 (5)	184 (8)^a^
**Inflammation**					
Highest White blood cell count, median (IQR), x10^9^/L	9 (7, 13)	9 (7, 13)^a^	10 (8, 14)^a^	9 (7, 12)	9 (7, 12)
Highest Premature neutrophils (bands), median (IQR), %	10 (4, 20)	10 (4, 17)^a^	12 (5, 24)^a^	5 (2, 14)	8 (3, 15)
Lowest Lymphocytes, median (IQR), %	16 (9, 24)	16 (9, 26)^a^	12 (6, 20)^a^	18 (11, 26)	17 (10, 24)^a^
C-reactive protein, tested, n (%)	5,862 (14)	1,479 (12)^a^	1,694 (17)^a^	1,730 (13)	959 (16)^a^
Highest C-reactive protein, median (IQR), mg/L	18 (5, 77)	18 (5, 71)^a^	53 (11, 122)^a^	11 (3, 54)	12 (4, 52)
Erythrocyte sedimentation rate, tested, n (%)	3,903 (9)	962 (8)^a^	1,021 (11)	1,234 (10)	686 (11)^a^
Highest Erythrocyte sedimentation rate, median (IQR), mm/h	40 (19, 73)	37 (18, 66)	51 (23, 88)^a^	34 (17, 65)	40 (20, 72)^a^
Highest Temperature, mean (SD), Celsius	37.7 (0.6)	37.7 (0.6)^a^	37.9 (0.8)^a^	37.6 (0.5)	37.7 (0.6)^a^
38–39, n (%)	8,633 (21)	2,869 (23)^a^	2,349 (24)^a^	2,259 (17)	1,156 (19)
> 39, n (%)	1,548 (4)	349 (3)^a^	826 (9)^a^	211 (2)	162 (3)^a^
Lowest Temperature, mean (SD), Celsius	36.7 (1.0)	36.5 (1.4)^a^	36.7 (0.8)^a^	36.7 (0.8)	36.8 (0.7)^a^
**Hematologic**					
Lowest Hemoglobin, mean (SD), g/dL	11.5 (2.3)	11.1 (2.3)^a^	11.2 (2.4)^a^	12.0 (2.2)	12.0 (2.3)
Highest RDW, mean (SD), %	15.5 (2.1)	15.5 (2.2)^a^	15.9 (2.3)^a^	15.2 (1.9)	15.5 (2.0)^a^
Lowest Platelets, median (IQR), x10^9^/L	210 (161, 269)	200 (152, 258)^a^	218 (161, 285)^a^	211 (166, 265)	219 (169, 274)^a^
Platelets < 200, n (%), x10^9^/L	16,707 (40)	5,535 (44)^a^	3,874 (40)^a^	4,971 (38)	2,327 (38)^a^
< 100	2,643 (16)	976 (18)^a^	785 (20)^a^	628 (13)	254 (11)
100–200	14,064 (84)	4,559 (82)^a^	3,089 (80)^a^	4,343 (87)	2,073 (89)
International normalized ratio, tested, n (%)	20,357 (49)	5,607 (44)^a^	5,193 (53)^a^	6,150 (47)	3,407 (56)^a^
>= 2	1,836 (9)	586 (10)^a^	583 (11)^a^	465 (8)	202 (6)^a^
**Neurologic**					
Glasgow Coma Scale score, n (%)					
Moderate neurologic dysfunction (9–12)	1,708 (4)	631 (5)^a^	479 (5)^a^	401 (3)	197 (3)
Severe neurologic dysfunction (< = 8)	1,482 (4)	477 (4)^a^	479 (5)^a^	336 (3)	190 (3)
**Liver and metabolic**					
Bilirubin tested, n (%), mg/dL	21,183 (51)	5,431 (43)^a^	5,902 (61)^a^	6,110 (47)	3,740 (61)^a^
≥ 2	1,427 (7)	527 (10)^a^	481 (8)^a^	306 (5)	113 (3)^a^
Highest Glucose, median (IQR), mg/dL	126 (104, 170)	125 (102, 165)	129 (105, 175)^a^	124 (102, 167)	132 (106, 186)^a^
Albumin, tested, n (%)	21,368 (51)	5,508 (43)^a^	5,929 (61)^a^	6,172 (48)	3,759 (61)^a^
< 2.5	1,243 (6)	403 (7)^a^	555 (9)^a^	180 (3)	105 (3)
2.5–3.5	6,904 (32)	1,912 (35)^a^	2,292 (39)^a^	1,621 (26)	1,079 (29)

Abbreviations: ICU: intensive care unit; IMC: intermediate care unit; MAP: mean aterial pressure; RDW: red cell distribution width; SD: standard deviation; IQR: interquartile range.

^a^ The p-values represent difference < 0.05 compared to Physiotype C and were adjusted for multiple comparisons using Bonferroni method. Supplemental Tables list p values for all within-group comparisons.

^b^ Cardiovascular disease was considered if there was a history of congestive heart failure, coronary artery disease of peripheral vascular disease.

^c^ Reference glomerular filtration rate and reference creatinine were derived without use of race correction (see S1 Text for details).

**Table 2:** Physiotype illness severity, clinical outcomes, and resource use.

Variables	Total	Acute Illness Physiotypes			
		Physiotype A	Physiotype B	Physiotype C	Physiotype D
Number of encounters (%)	41,502	12,695 (31)	9,710 (23)	12,962 (31)	6,135 (15)
**Acuity scores within 24h of admission**					
SOFA score > 6, n (%)	3,506 (8)	1,494 (12)^a^	974 (10)^a^	720 (6)	318 (5)
Patients in ICU/IMC, SOFA score ≤ 6, n (%)	6,882 (17)	1,868 (15)^a^	2,195 (23)^a^	1,693 (13)	1,126 (18)^a^
Patients in ICU/IMC, SOFA score > 6, n (%)	2,544 (6)	1,025 (8)^a^	827 (9)^a^	458 (4)	234 (4)
Patients on ward, SOFA score ≤ 6, n (%)	31,114 (75)	9,333 (74)^a^	6,541 (67)^a^	10,549 (81)	4,691 (76)^a^
Patients on ward, SOFA score > 6, n (%)	962 (2)	469 (4)^a^	147 (2)^a^	262 (2)	84 (1)^a^
MEWS score ≥ 5, n (%)	2,828 (7)	472 (4)^a^	1,549 (16)^a^	264 (2)	543 (9)^a^
Patients in ICU/IMC, MEWS score ≤ 4, n (%)	7,235 (17)	2,507 (20)^a^	1,785 (18)^a^	1,941 (15)	1,002 (16)
Patients in ICU/IMC, MEWS score > 4, n (%)	2,191 (5)	386 (3)^a^	1,237 (13)^a^	210 (2)	358 (6)^a^
Patients on ward, MEWS score ≤ 4, n (%)	31,439 (76)	9,716 (77)^a^	6,376 (66)^a^	10,757 (83)	4,590 (75)^a^
Patients on ward, MEWS score > 4, n (%)	637 (2)	86 (1)^a^	312 (3)^a^	54 (0)	185 (3)^a^
**Resource use during hospitalization**					
Hospital days, median (IQR)	4 (2, 7)	4 (2, 6)^a^	4 (3, 8)^a^	3 (2, 6)	4 (2, 7)^a^
Surgery at any time, n (%)	11,634 (28)	5,225 (41)^a^	1502 (15)^a^	3957 (31)	950 (15)^a^
Admitted to ICU/IMC^b^, n (%)	11,121 (27)	3,330 (26)^a^	3,504 (36)^a^	2,640 (20)	1,647 (27)^a^
Days in ICU/IMC^c^, median (IQR)	4 (2, 7)	4 (3, 7)^a^	4 (3, 8)^a^	4 (2, 7)	4 (2, 6)
ICU/IMC stay greater than 48 hrs, n (%)	8,332 (75)	2,517 (76)^a^	2,722 (78)^a^	1,872 (71)	1,221 (74)
Mechanical ventilation, n (%)	3,218 (8)	1,120 (9)^a^	1,036 (11)^a^	736 (6)	326 (5)
Mechanical ventilation hours, median (IQR)^d^	35 (14, 113)	24 (11, 81)	46 (17, 142)^a^	26 (12, 105)	54 (21, 145)^a^
Mechanical ventilation greater than 2 calendar days, n (%)	1,661 (52)	492 (44)	613 (59)^a^	349 (47)	207 (63)^a^
Renal replacement therapy, n (%)	1,262 (3)	335 (3)^a^	299 (3)^a^	265 (2)	363 (6)^a^
**Complications**					
Acute kidney injury, n (%)	6905 (17)	1,971 (16)^a^	2,119 (22)^a^	1,682 (13)	1,133 (18)^a^
Community-acquired AKI, n (%)	3839 (56)	1,234 (63)^a^	1,221 (58)^a^	873 (52)	511 (45)^a^
Hospital-acquired AKI, n (%)	3066 (44)	737 (37)^a^	898 (42)^a^	809 (48)	622 (55)^a^
Worst AKI staging, n (%)					
Stage 1	4360 (63)	1,194 (61)^a^	1,241 (59)^a^	1,174 (70)	751 (66)
Stage 2	1362 (20)	404 (20)^a^	484 (23)^a^	280 (17)	194 (17)
Stage 3	848 (12)	269 (14)^a^	276 (13)^a^	171 (10)	132 (12)
Stage 3 with RRT	335 (5)	104 (5)^a^	118 (6)^a^	57 (3)	56 (5)
Venous thromboembolism, n (%)	1257 (3)	341 (3)	393 (4)^a^	350 (3)	173 (3)
Sepsis, n (%)	3750 (9)	902 (7)^a^	1,933 (20)^a^	500 (4)	415 (7)^a^
Hospital Disposition, n (%)					
Hospital mortality	1141 (3)	291 (2)^a^	502 (5)^a^	227 (2)	121 (2)
Another hospital, LTAC, SNF, Hospice	4475 (11)	1,286 (10)	1,231 (13)^a^	1,233 (10)	725 (12)^a^
Home or short-term rehabilitation	35886 (86)	11,118 (88)^a^	7,977 (82)^a^	11,502 (89)	5,289 (86)^a^
Thirty-day mortality, n (%)	1633 (3.9)	429 (3)^a^	684 (7)^a^	332 (3)	188 (3)
Three-year mortality, n (%)	8013 (19)	2,205 (17)	2,466 (25)^a^	2,109 (16)	1,233 (20)^a^

Abbreviation: SOFA: sequential organ failure assessment; MEWS: modified early warning score; ICU: intensive care unit; IMC: intermediate care unit; IQR: interquartile range.

^a^ The p-values represent difference < 0.05 compared to Physiotype C and were adjusted for multiple comparisons using Bonferroni method. Supplemental Tables list p values for all within-group comparisons.

^b^ At any time during hospitalization.

^c^ Values were calculated among patients admitted to ICU/IMC.

^d^ Values were calculated among patients requiring MV.

#### Physiotype A

*Physiotype* A exhibited early and persistent hypotension without concomitant rise in HR, high incidence of vasopressor support (32%), initial normothermia followed by decreasing body temperature, and low RR with high SpO2, consistent with having the highest proportion undergoing early surgery (35%). Despite high incidence of surgical interventions, *Physiotype* A had lower inflammatory markers (i.e., C-reactive protein, erythrocyte sedimentation rate) than two of the other three *physiotypes*. Despite early, severe illness, *Physiotype* A had favorable short-and long-term clinical outcomes, consistent with reversible surgical disease and evident by the greatest proportion of patients with SOFA score > 6 within 24 hours of admission (12%) but the second-lowest incidence of ICU/IMC admission (26%), AKI (16%), and three-year mortality (17%).

#### Physiotype B

*Physiotype* B exhibited early tachycardia, tachypnea, and hypoxemia. Unlike similarly hypotensive *Physiotype* A, *Physiotype* B had substantial biomarker evidence of inflammation, evident by the highest levels of C-reactive protein (53 mg/L compared with 11–18 mg/L in all other *physiotypes*) and erythrocyte sedimentation rate. The *Physiotype* B biomarker profile also suggested infection and perfusion deficit manifested as higher white blood cell counts, and base deficit values (6.4 vs. 3.6–4.4 mmol/L in other *physiotypes*). *Physiotype* B had the highest incidence of prolonged respiratory insufficiency (11% receiving mechanical ventilation, 59% of whom received more than 2 calendar days of ventilator support), sepsis (20%, compared with 4–7% in the other *physiotypes*), acute kidney injury (22%), hospital mortality (5%, more than two-fold greater than all other *physiotypes*) and three-year mortality (25%).

#### Physiotype C

*Physiotype* C had minimal early physiological derangement and a diffuse pattern of mild organ dysfunction. *Physiotype* C had favorable clinical outcomes manifest as the lowest incidence of ICU/IMC admission (20%), AKI (13%), sepsis (4%), hospital mortality (2%), and three-year mortality (16%), despite having comorbid disease burdens like other *physiotypes* (cardiovascular disease: 29% vs. 27–32%, diabetes: 24% vs. 23–27%, chronic kidney disease: 16% vs. 14–20%).

#### Physiotype D

*Physiotype* D had the greatest prevalence of chronic cardiovascular and kidney disease (32% and 20%, respectively), the greatest proportions of African American patients (37% vs. 16–23% in other *physiotypes*) and emergent admissions (89%), and presented with severely elevated blood pressure; 79% had a systolic blood pressure measurement greater than 160 mmHg. *Physiotype* D had the second highest incidence of ICU/IMC admission (27%) despite having the lowest proportion of patients with SOFA > 6 (5%) and had 2% hospital mortality but suffered 20% 3-year mortality.

### Vital sign signatures

**Fig 2:**
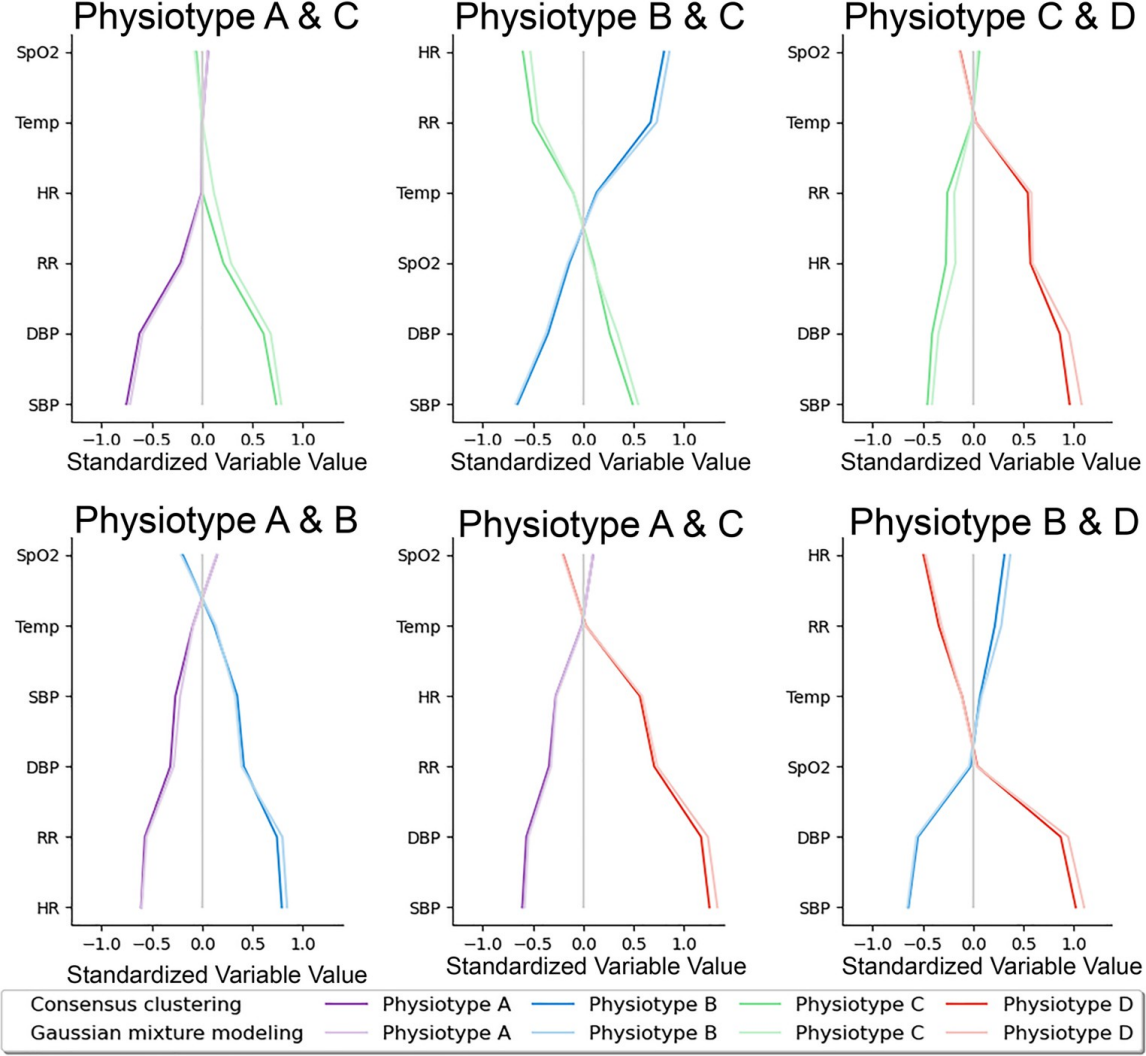
Vital sign contributions to cluster assignments. Pairwise physiotype comparisons of vital sign values standardized to mean 0 and standard deviation 1 demonstrated that temperature and oxygen saturation contributed least to phenotype differences. Systolic and diastolic blood pressure varied substantially between all Physiotypes except for A and B. SpO2: peripheral capillary oxygen saturation; Temp: temperature; SBP: systolic blood pressure; DBP: diastolic blood pressure, RR: respiratory rate; HR: heart rate.

To understand which vital signs made the greatest contributions to cluster assignments, vital sign standardized mean differences were compared between pairs of phenotypes (Fig 2). Temperature and oxygen saturation contributed least to phenotype differences. Systolic and diastolic blood pressure varied substantially between all *physiotypes* except for A and B. Fig 2 demonstrates that consensus clustering and Gaussian mixture modeling yielded similar standardized variable values. Fig 1C and S5 Fig illustrate average vital sign mosaics for each *physiotype* and individual vital sign mosaics for two example patients, demonstrating how each *physiotype* had a unique overall mosaic that was representative of individual patient mosaics.

### SOFA scores

Associations between *physiotypes* and highest SOFA score within first 24 hours of admission are illustrated in S6 Fig; SOFA components for each *physiotype* are illustrated in chord diagrams in S7 Fig. *Physiotypes* A and B had the greatest proportions of patients with cardiovascular and respiratory dysfunction. Yet, each *physiotype* contained substantial proportions of patients across the full range of SOFA scores and component subscores; clustering did not simply recapitulate SOFA acuity assessments.

### Survival probabilities

**Fig 3:**
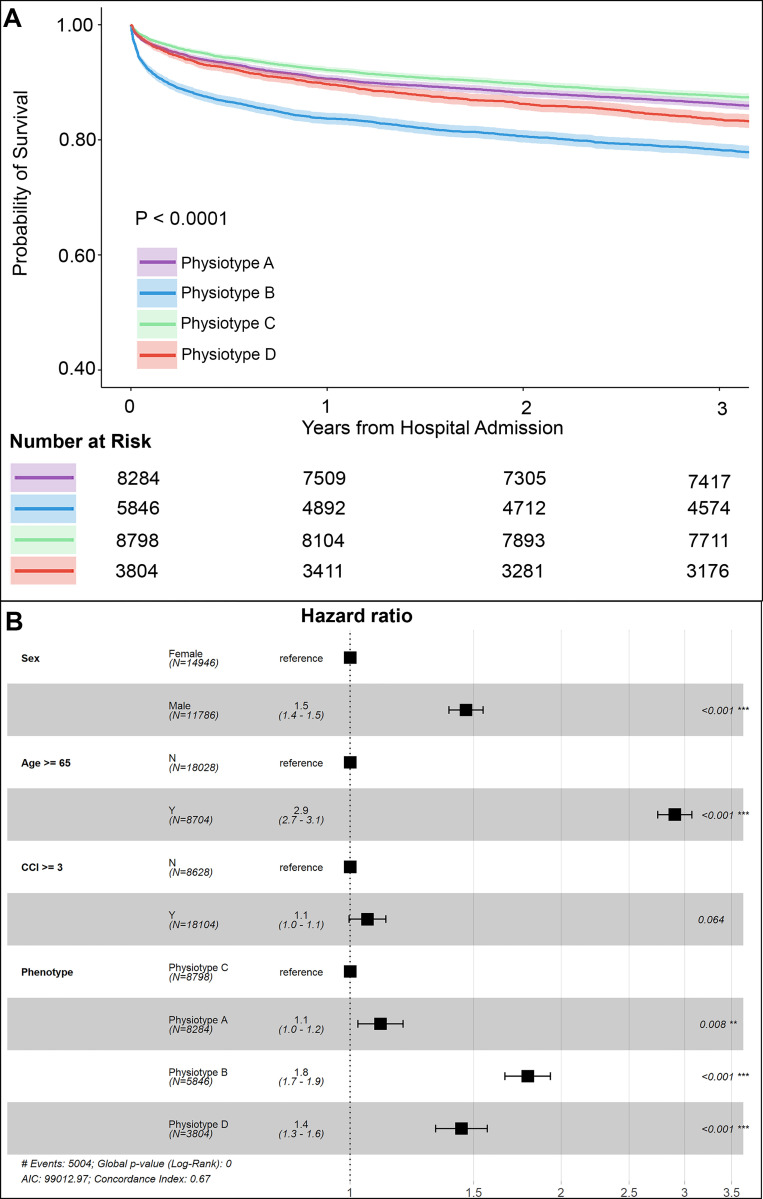
Survival curves and Cox proportional hazards modeling. (A) Physiotype survival curves adjusted using demographic information and comorbidities. (B) Adjusted Cox proportional hazards models using demographic information and comorbidities. CCI: Charlson Comorbidity Index.

Three-year survival probability was modeled adjusting for demographics and comorbidities (Fig 3), demonstrating lower probability of survival for male sex (HR 1.5, 95% CI 1.4–1.5), and age 65 years or greater (HR 2.9, 95% CI 2.7–3.1). Using *Physiotype* C as a reference, probability of survival was lower for *Physiotype* A (HR 1.1, 95% CI 1.0–1.2), D (HR 1.4, 95% CI 1.3–1.6), and B (HR 1.8, 95% CI 1.7–1.9, all p<0.008). Three-year survival probability was similarly modeled, (S8C and S8D Figs), demonstrating similar associations among demographics, comorbidities and survival, and strong associations between higher SOFA and lower survival probability (SOFA 2–4: HR 1.6, 95% CI 1.5–1.7; SOFA 5 or greater: HR 2.3, 95% CI 2.1–2.4, all p<0.001). When adjusting for SOFA, *Physiotype* A was not associated with lower survival probability (HR 1.0, 95% CI 0.9–1.0, p = 0.146), but *Physiotypes* D and B were (HR 1.5, 95% CI 1.4–1.7 and HR 1.7, 95% CI 1.6–1.8, respectively).

### Reproducibility

Proportions of the total cohort in each *physiotype* were stable across training, validation, and testing (*Physiotype* A: 31%, 30%, and 30%, respectively; *Physiotype* B: 23%, 23%, and 24%, respectively; *Physiotype* C: 31%, 31%, and 31%, respectively; *Physiotype* D: 15%, 16%, and 15%, respectively). *Physiotypes* derived in the validation cohort using consensus *k*-means clustering showed similar clinical characteristics, biomarkers, and patient outcomes as observed in the validation cohort (S9–S12 Figs, S7 and S8 Tables). No significant differences were observed after excluding temperature values with high missingness (S13 Fig, S9 and S10 Tables), or after excluding variables with high missingness and correlation (S14 Fig, S11 and S12 Tables). Similar trends were observed when using 12-hour vital sign window (S15 Fig, S13 and S14 Tables). *Physiotypes* were also reproducible in testing data (S15 Table). The clinical characteristics, biomarkers, and patient outcomes of *physiotypes* predicted in the testing cohort mimicked the training cohort (S16 and S17 Figs, S16 and S17 Tables). SOFA score distributions, survival curves, and diagnosis groups were similar across training, validation, and testing cohorts (S18–S26 Figs). Gaussian mixture modeling method confirmed the statistical fit of the 4-class model (S27 Fig and S18 Table). *Physiotypes* identified by Gaussian mixture modeling had vital sign distributions and t-distributed stochastic neighbor embedding plots that were similar to those originally derived by consensus clustering (S28–S30 Figs, S19 and S20 Tables).

## Discussion

Using six vital signs measured within six hours of hospital admission, consensus clustering identified four distinct, clinically relevant patient phenotypes with unique pathophysiological signatures, disease categories, and clinical outcomes. Blood pressure values and trends contributed substantially to cluster assignments: one hypertensive, one normotensive, and two hypotensive clusters. Among the two hypotensive clusters, one was inflammatory, the other non-inflammatory according to C-reactive protein and erythrocyte sedimentation rate values. Beyond these fundamental distinctions, clusters were also differentiated by disease categories, producing the final *physiotype* labels. *Physiotype* A, hypotensive non-inflammatory surgical shock, had physiologic signals suggesting early vasoplegia and hypothermia but low-grade inflammation relative to *Physiotype* B, a hypotensive inflammatory pulmonary dysfunction *physiotype* associated with early tachycardia, tachypnea, and hypoxemia followed by greatest burdens of prolonged respiratory insufficiency, sepsis, acute kidney injury, and short- and long-term mortality. *Physiotype* C, a normotensive, rapid normalization *physiotype*, had minimal early physiological derangement and favorable clinical outcomes. *Physiotype* D, hypertensive chronic disease exacerbation, had greatest prevalence of chronic cardiovascular and kidney disease, presented with severely elevated blood pressure, and had favorable short-term outcomes but suffered 20% three-year mortality. Each *physiotype* contained substantial patient proportions across the full ranges of SOFA scores and component subscores, suggesting that clustering did not simply recapitulate SOFA acuity assessments. Finally, *physiotype* characteristics were reproduced with fidelity in validation and testing cohorts.

Beyond the potential to augment understanding of pathophysiology by distilling thousands of disease states into a few physiological signatures, *physiotypes* could be adapted to augment clinical decision-making under time constraints and uncertainty. Early identification of hypotensive inflammatory pulmonary dysfunction could theoretically facilitate early ICU admission and high suspicion for sepsis with attention to resuscitation strategies that maintain adequate renal perfusion without inducing volume overload and hydrostatic pulmonary edema, primarily by focusing on providing the optimal balance of intravenous fluid resuscitation and vasopressor [5–8,22]. Early identification of normotensive rapid recovery could facilitate early hospital discharge or triage to low-intensity care settings (i.e., hospital floors), avoiding excessive monitoring testing that confers lower value of care and may impart harm from unnecessary treatments [9,10]. Early identification of hypertensive chronic disease exacerbation could suggest low value for critical care resources compared with careful post-discharge follow-up for mitigating long-term mortality, and could be built into a decision-support system that facilitates hospital ward admission and outpatient clinic visits to address modifiable risk factors and optimize medication regimens for treating the underlying chronic disease. Several statistical and machine learning methods can accurately predict risk for death, but these approaches do not elucidate pathophysiologic states or disease categories [23,24]. Conversely, clustering can identify patient phenotypes that have unique disease states and mortality risk, representing a potentially useful adjunct to clinical decision-support systems, particularly among heterogeneous patient cohorts with diverse disease etiologies.

We are unaware of previous studies using cluster analyses of early vital sign measurements to identify phenotypes in heterogeneous cohorts of patients hospitalized for any reason. Others have used clustering for identifying patients with unique disease subtypes with unique treatment responses; sepsis and diastolic heart failure are prominent examples. Seymour et al. [15] performed clustering analyses on a multi-center cohort of sepsis patients with the rationale that sepsis pathophysiology is heterogeneous and identifying distinct sepsis phenotypes may facilitate targeted therapy. Clustering was performed on both clinical and host immune response biomarker variables, identifying four distinct clusters. In a series of simulations, varying proportions of each cluster were applied to previously reported randomized controlled trials. Treatment effects varied significantly across simulations, suggesting unique treatment responses. Shah et al. [25] performed clustering analyses on a single-center cohort of patients with heart failure and preserved ejection fraction, another heterogeneous syndrome refractory to one-size-fits-all management. Clustering was performed on electrocardiogram and echocardiogram data as well as clinical variables, identifying three distinct phenotypes with unique risk-adjusted clinical outcomes. While Seymour et al. [15] and Shah et al. [25] both identified subgroups of patients within larger patient groups that share an established diagnosis, we instead apply clustering methods to any hospitalized patient, identifying broad, generalized patterns of pathophysiology rather than targeted treatment responses. This difference precludes further comparison of our results with others.

We also acknowledge several limitations. Our study used data from a single institution, limiting the generalizability of our findings, and external validation in databases from different centers is needed. Yet, it seems unlikely that selection bias significantly affected results, as all adult patients admitted for longer than six hours were included. Input variables were limited to the first six hours following hospital admission so that phenotypes could be identified early enough to support clinical decision-making under time constraints and uncertainty. It is possible that the same advantages for early decision-support could be achieved while incorporating historical patient data from previous encounters in the electronic health record; further research is necessary to determine whether this strategy is advantageous. Waveform data, though not universally available in EHRs, has the potential to improve the precision of phenotype clustering. Our clustering approach does not ensure temporal ordering of vital signs, which could influence cluster assignments. Finally, the potential of early clustering to augment clinical decision-making remains theoretical until evaluated in a prospective trial.

## Conclusions

Using six vital signs measured within six hours of hospital admission, clustering analyses identified four distinct patient phenotypes that had unique disease categories and clinical outcomes and did not recapitulate previously established acuity assessments. Beyond elucidating pathophysiology by distilling thousands of disease states into a few physiological signatures, identifying patient phenotypes during the early stages of hospital admission may have important implications for clinical decision-making under time constraints.

## Supporting information

S1 Text Detailed description of methods. (DOCX)

S1 Fig Cohort selection and exclusion criteria. (DOCX)

S2 Fig Purposes of training, validation, and testing cohorts. (DOCX)

S3 Fig Consensus k clustering results in training cohort (N = 41,502). (A) Unsupervised consensus k clustering in training cohort showing optimal partitioning in consensus matrix for k = 4. (B) Consensus cumulative distribution function (CDF) across k = 2 to k = 8, where more horizontal curves suggest optimal fit. (C) Relative change in the area under the CDF curve with increasing clusters (k), with little change beyond k = 4. (D) Cluster consensus plot showing the mean of all pairwise consensus values between a cluster members, for k = 2 to k = 8 where greater values for all bars suggest optimal fit.
(DOCX)

S4 Fig Spearman correlation heat map for the training cohort (N = 41,502). Spearman correlation heat map shows the pairwise spearman rank order correlation coefficient among the 6 vital signs studied in our paper. The darker red color, the higher correlation in positive direction. Abbreviations: RR: respiratory rate; SpO2: peripheral capillary oxygen saturation; Temp: temperature; HR: heart rate; SBP: systolic blood pressure; DBP: diastolic blood pressure.
(DOCX)

S5 Fig Average vital sign mosaics of phenotypes using a self-organizing map in the training cohort (N = 41,502). (DOCX)

S6 Fig Alluvial plot showing distribution of phenotypes across worst SOFA scores of patients within first 24 hours of admission in training cohort. For each phenotype, the larger percentage of patients with that score, the broader the ribbon.
(DOCX)

S7 Fig Chord diagrams showing the distribution of patients with higher SOFA scores (i.e., 2+) within first 24 hours of admission of six organ systems by phenotypes in training cohort. For each phenotype, the larger percentage of patients with higher score of that organ system, the border the ribbon.
(DOCX)

S8 Fig Survival curves and Cox proportional hazards modeling by phenotypes in training cohort. (A) Physiotype survival curves adjusted using demographic information and comorbidities. (B) Adjusted Cox proportional hazards models using demographic information and comorbidities. (C) Physiotype survival curves adjusted using demographic information, comorbidities, and SOFA scores. (D) Adjusted Cox proportional hazards model using demographic information, comorbidities, and SOFA scores. Abbreviation: CCI: charlson comorbidity index; SOFA: sequential organ failure assessment.
(DOCX)

S9 Fig Consensus k clustering results in validation cohort (N = 17,415). (A) Unsupervised consensus k clustering in training cohort showing optimal partitioning in consensus matrix for k = 4. (B) Consensus cumulative distribution function (CDF) across k = 2 to k = 8, where more horizontal curves suggest optimal fit. (C) Relative change in the area under the CDF curve with increasing clusters (k), with little change beyond k = 4. (D) Cluster consensus plot showing the mean of all pairwise consensus values between a cluster members, for k = 2 to k = 8 where greater values for all bars suggest optimal fit.
(DOCX)

S10 Fig Mean standardized differences between variables across phenotype pairs for training cohort (N = 41,502, dark line) and validation cohort (N = 17,415) using consensus clustering. In all panels, the variables are standardized such that all means are scaled to 0 and SDs to 1. A value of 1 for the standardized variable (x-axis) signifies that the mean value for the phenotype was 1 SD higher than the mean value for both phenotypes shown in the graph as a whole. Abbreviations in order: SpO2: peripheral capillary oxygen saturation; Temp: temperature; SBP: systolic blood pressure; DBP: diastolic blood pressure, RR: respiratory rate; HR: heart rate.
(DOCX)

S11 Fig Validation cohort clusters had unique distributions of vital signs during the first six hours of admission. (DOCX)

S12 Fig t-SNE plot of penotype assignments in validation cohort. Starting from the original 36 dimensional vital signs, we run the t-SNE to reduce to 2 dimensions. Each dot represents a patient. Phenotypes are shown in separate colors.
(DOCX)

S13 Fig Distribution of vital signs by phenotypes in sensitivity analysis excluding variables with high missingness (temperature) in training cohort (N = 41,502). (DOCX)

S14 Fig Distribution of vital signs by phenotypes in sensitivity analysis excluding variables with high correlation (diastolic blood pressure and respiratory rate) and high missingness (temperature) in training cohort (N = 41,502). (DOCX)

S15 Fig Distribution of vital signs by phenotypes in sensitivity analysis using a 12-hour window of EHR data in the training cohort (N = 41,502). (DOCX)

S16 Fig Testing cohort clusters had unique distributions of vital signs during the first six hours of admission. (DOCX)

S17 Fig t-SNE plot of phenotype assignments in testing cohort. Starting from the original 36 dimensional vital signs, we run the t-SNE to reduce to 2 dimensions. Each dot represents a patient. Phenotypes are shown in separate colors.
(DOCX)

S18 Fig Alluvial plot showing distribution of phenotypes across worst SOFA scores of patients within first 24 hours of admission in validation cohort. For each phenotype, the larger percentage of patients with that score, the broader the ribbon.
(DOCX)

S19 Fig Chord diagrams showing the distribution of patients with higher SOFA scores (i.e., 2+) within first 24 hours of admission of six organ systems by phenotypes in validation cohort. For each phenotype, the larger percentage of patients with higher score of that organ system, the border the ribbon.
(DOCX)

S20 Fig Alluvial plot showing distribution of phenotypes across worst SOFA scores of patients within first 24 hours of admission in testing cohort. For each phenotype, the larger percentage of patients with that score, the broader the ribbon.
(DOCX)

S21 Fig Chord diagrams showing the distribution of patients with higher SOFA scores (i.e., 2+) within first 24 hours of admission of six organ systems by phenotypes in testing cohort. For each phenotype, the larger percentage of patients with higher score of that organ system, the border the ribbon.
(DOCX)

S22 Fig Survival curves and Cox proportional hazards modeling by phenotypes in validation cohort. (A) Physiotype survival curves adjusted using demographic information and comorbidities. (B) Adjusted Cox proportional hazards models using demographic information and comorbidities. (C) Physiotype survival curves adjusted using demographic information, comorbidities, and SOFA scores. (D) Adjusted Cox proportional hazards model using demographic information, comorbidities, and SOFA scores. Abbreviation: CCI: charlson comorbidity index; SOFA: sequential organ failure assessment.
(DOCX)

S23 Fig Survival curves and Cox proportional hazards modeling by phenotypes in testing cohort. (A) Physiotype survival curves adjusted using demographic information and comorbidities. (B) Adjusted Cox proportional hazards models using demographic information and comorbidities. (C) Physiotype survival curves adjusted using demographic information, comorbidities, and SOFA scores. (D) Adjusted Cox proportional hazards model using demographic information, comorbidities, and SOFA scores. Abbreviation: CCI: charlson comorbidity index; SOFA: sequential organ failure assessment.
(DOCX)

S24 Fig Chord diagrams showing the distribution of nine most common admission diagnosis groups by phenotype in training cohort. Diagnosis groups are shown in order of frequencies of all patients. For each phenotype, the larger percentage of patients with that diagnosis, the border the ribbon. Detailed diagnosis groups from left to right are: Nonspecific chest pain, Abdominal pain, Other and unspecific lower respiratory disease, Complication of device; implant or graft, Speticemia (except in labor), Acute cerebrovascular disease, Cardiac dysrhythmias, Congestive heart failure; nonhypertensive, and Osteoarthritis.
(DOCX)

S25 Fig Chord diagrams showing the distribution of nine most common admission diagnosis groups by phenotype in validation cohort. Diagnosis groups are shown in order of frequencies of all patients. For each phenotype, the larger percentage of patients with that diagnosis, the border the ribbon. Detailed diagnosis groups from left to right are: Nonspecific chest pain, Abdominal pain, Complication of device; implant or graft, Other and unspecific lower respiratory disease, Speticemia (except in labor), Malaise and fatigue, Acute cerebrovascular disease, Osteoarthritis, and Cardiac dysrhythmias.
(DOCX)

S26 Fig Chord diagrams showing the distribution of nine most common admission diagnosis groups by phenotype in testing cohort. Diagnosis groups are shown in order of frequencies of all patients. For each phenotype, the larger percentage of patients with that diagnosis, the border the ribbon. Detailed diagnosis groups from left to right are: Nonspecific chest pain, Other and unspecific lower respiratory disease, Speticemia (except in labor), Abdominal pain, Complication of device; implant or graft, Acute cerebrovascular disease, Cardiac dysrhythmias, Osteoarthritis, and Other complications of pregnancy.
(DOCX)

S27 Fig Sensitivity analysis using gaussian mixture modeling clustering in training cohort (N = 41,502), showing probabilities of phenotype assignment. Interpretive example: Using gaussian mixture modeling to derive phenotypes, histograms of within phenotype probability demonstrated that members have high probability of being a phenotype member (>0.9).
(DOCX)

S28 Fig Distribution of vital signs by phenotypes derived using gaussian mixture modeling clustering in the training cohort (N = 41,502). (DOCX)

S29 Fig t-SNE plot of phenotype assignments in training cohort. Visualization of phenotypes using t-distributed stochastic neighbor embedding (t-SNE) technique in the training cohort with (A) physiotypes derived by consensus clustering shown in color, and (B) physiotypes derived by gaussian mixture modeling (GMM) shown in color.
(DOCX)

S30 Fig Probabilities of assignment for phenotype members and for those not assigned, using gaussian mixture modeling in the training cohort (N = 41,502). (A) Probabilities of assignment to cluster 1, and purple for those actually assigned to cluster 1, (B) Probabilities for patients assigned to cluster 2, and blue for those actually assigned to cluster 2, (C) Probabilities for patients assigned to cluster 3, and green for those actually assigned to cluster 3, and (D) probabilities for patients assigned to cluster 4, and orange for those actually assigned to cluster 4. Black lines correspond to median [IQR] of probability. Gray shading corresponds to region with a 45–55% (low or marginal) probability of assignment. Inset proportion is the % of 41,502 in the marginal region.
(DOCX)

S1 Table Processing of vital sign time series. (DOCX)

S2 Table Used LOINCS, range of values, direction of abnormal values for lab variables. (DOCX)

S3 Table Clinical characteristics and biomarkers of the cohorts. (DOCX)

S4 Table Illness severity, clinical outcomes, and resource use of the cohorts. (DOCX)

S5 Table Physiotype clinical characteristics and biomarkers in the training cohort. (DOCX)

S6 Table Physiotype illness severity, clinical outcomes, and resource use in the training cohort. (DOCX)

S7 Table Physiotype clinical characteristics and biomarkers in the validation cohort. (DOCX)

S8 Table Physiotype illness severity, clinical outcomes, and resource use in the validation cohort. (DOCX)

S9 Table Physiotype clinical characteristics and biomarkers in sensitivity analysis by excluding highly missing variable (Temperature) in the training cohort. (DOCX)

S10 Table Physiotype illness severity, clinical outcomes, and resource use in sensitivity analysis by excluding highly missing variable (Temperature) in the training cohort. (DOCX)

S11 Table Physiotype clinical characteristics and biomarkers in sensitivity analysis by excluding variables with high missingness (temperature) and correlation (diastolic blood pressure and respiratory rate) in the training cohort. (DOCX)

S12 Table Physiotype illness severity, clinical outcomes, and resource use in sensitivity analysis by excluding variables with high missingness (temperature) and correlation (diastolic blood pressure and respiratory rate) in the training cohort. (DOCX)

S13 Table Physiotype clinical characteristics and biomarkers in sensitivity analysis by using a 12 hour window of EHR data in the training cohort. (DOCX)

S14 Table Physiotype illness severity, clinical outcomes, and resource use in sensitivity analysis by using a 12 hour window of EHR data in the training cohort. (DOCX)

S15 Table Centroids of physiotypes for prediction. (DOCX)

S16 Table Physiotype clinical characteristics and biomarkers in the testing cohort. (DOCX)

S17 Table Physiotype illness severity, clinical outcomes, and resource use in the testing cohort. (DOCX)

S18 Table Statistical output from gaussian mixture modeling in the training cohort (N = 41,502). (DOCX)

S19 Table Physiotype clinical characteristics and biomarkers by physiotypes derived using gaussian mixture modeling in sensitivity analysis in the training cohort. (DOCX)

S20 Table Physiotype illness severity, clinical outcomes, and resource use by physiotypes derived using gaussian mixture modeling in sensitivity analysis in the training cohort. (DOCX)
