# Supplementary material for: Physiologic signatures within six hours of hospitalization identify acute illness phenotypes
Source: PLOS Digit Health. 2022 Oct 13;1(10):e0000110. doi: 10.1371/journal.pdig.0000110 (PMC9802629; doi:10.1371/journal.pdig.0000110)
Supplement: S1 Text — (DOCX) [file pdig.0000110.s001.docx]

# S1 Text

1. **Data source and participants and study design**

**Data source and participants**

This project was approved by the University of Florida institutional review board under a waiver of informed consent and with authorization under the Health Insurance Portability and Accountability Act. Transparent Reporting of a multivariable prediction model for Individual Prognosis Or Diagnosis (TRIPOD) recommendations were followed under the Type 2b analysis category (nonrandom split-sample development and validation). Using the University of Florida Health (UFH) Integrated Data Repository as Honest Broker, we created a longitudinal dataset from electronic health records of all adults (age ≥18 years) admitted to the 1000-bed academic hospital at UFH between June 1, 2014 and April 1, 2016 with length of stay greater than or equal to six hours including emergency department admission if applicable. The dataset includes structured and unstructured clinical data, demographic information, vital signs, laboratory values, medications, diagnoses, and procedures. Patients completely missing at least two of the six vital sign measurements used for clustering (systolic and diastolic blood pressure, heart rate, respiratory rate, temperature, and peripheral capillary oxygen saturation) in the first six hours of admission were excluded from the analysis (eFigure 1). The final cohort consisted of 75,762 hospital admissions for 43,598 patients.

**Study design**

We non-randomly split the dataset by admission dates into three cohorts: training (admissions between June 1, 2014 and May 31, 2015, n = 41,502, 55% of all admissions), validation (admissions between June 1, 2015 and October 31, 2015, n = 17,415, 22% of all admissions), and testing (admissions between November 1, 2015 and April 1, 2016, n = 16,845, 23% of all admissions). To determine acute illness phenotypes using early physiologic signatures, we derived the clinical phenotypes using unsupervised clustering methods that were applied to the repeated measurements of six vital signs available within the first six hours of hospital presentation in the training cohort. We assessed phenotype reproducibility both by comparing phenotype derivation using alternative clustering methods in the initial data set and by exploring phenotype frequency distributions in testing cohort. (eFigure 2).

1. **Approach to preprocess electronics health records (EHR) data**

EHR vital data elements in our cohort studies were irregularly sampled time series. Prior to clustering algorithms, we first excluded outliers based on the expert-defined ranges (eTable 1). We then resampled raw time series to an hourly frequency, taking the mean value when multiple measurements existed during the same one-hour window. Following resampling, gaps in the resulting time series were filled by first forward-propagating previous values and then back-propagating posterior values[1]. For all remaining missing values, which is due to having no measurements during the hospitalization in the plausible range for a variable, median values of corresponding variables in the training cohort were imputed, as listed in eTable 1. We standardized values using Min-Max scaler (eTable 1).

1. **Consensus K clustering**

Consensus clustering, also known as cluster ensemble or aggregation of clustering, is designed to discover a single (consensus) partition that fits as much as possible with existing basic partitions[2]. The core idea of the algorithm is to merge complementary perspectives of the data into a more stable partition to improve the quality and robustness of clustering results. It has been broadly used in clinical data[3-5] and bioinformatics[6] fields.

Generally, a consensus clustering can be divided into two steps: 1) Generate the individual partitions to be combined. 2) Combine the partitions to generate the final partition. In the first step, a bootstrap, a resampling strategy by independently sampling with replacement, is repeatedly used to sample several subsets with the same sample size from the full data set. Then for each subset, an independent clustering algorithm is deployed to generate the corresponding partition result, named basic partition. In our case, we use the centroid-based classical K-means algorithm, considering its low computational cost and easy parallel process. In the second step, a “consensus matrix” is calculated based on all the basic partitions, and the matrix elements are “consensus rate” information between all pairs of samples. Specifically, the “consensus rate” indicates how frequently two samples are grouped together in all the basic partitions. This “consensus matrix” is further used for visual demonstration whether a final single partition exists, assessing the partition stability, and inferring the optimal cluster number. Eventually, a hierarchical clustering method is applied to this “consensus matrix” to generate a final cluster assignment for each sample.

There is widespread recognition that the consensus clustering can achieve some desirable clustering properties, such as robustness, stability, and novelty[7-9]. By inducing the resampling technique, it provides the consensus over multiple runs of a basic clustering method on different subsets. The ensemble combination strategy generally can achieve more robust and better performance than each basic partition. It is noteworthy that the basic clustering method can be initialized independently with different parameters. Aggregating these diverse and individual partitions can significantly improve the resulting stability and make clustering less sensitive to noise, outliers, and sample variations. Besides, the combination can also enable us to discover novel partitions that are not generated by the basic cluster methods. Based on the favorable properties, we decide to choose the consensus clustering as our clustering algorithms.

1. **Gaussian mixture model**

We applied gaussian mixture model (GMM) in a sensitivity analysis for deriving phenotypes in the training data. Similar to consensus *k* clustering, GMM can be used to (1) determine an optimal number of clusters for continuous data and (2) obtain cluster assignment for each subject[10]. For each subject and each cluster, GMM produces a posterior probability ranging from 0 to 1 describing the likelihood of a subject belonging to the cluster. We assigned each subject to the cluster with highest posterior probability. We determined the optimal number of clusters (*k*) using a combination of criteria, i.) a higher Bayesian information criterion, ii.) adequate sample size with cluster, iii.) high median posterior probabilities of group memberships and clinical features of potential clusters. Analysis was done with Mclust R package[11].

1. **Data visualization**
   - Chord plots

Chord diagram is widely used to represent connection and relationship between several entities. We generated two sets of chord diagrams to visualize the patients’ distribution regarding different studied variables.

One set of chord diagrams were created to visualize the distribution of phenotypes across worst SOFA scores of six organ systems within first 24 hours of admission. These six organ systems include:

- - - Cardiovascular
    - Respiratory
    - Coagulation
    - Liver
    - Neurologic
    - Renal

For each organ system, percent of patients with organ dysfunction, that is with SOFA score of 2 or more were calculated. For each phenotype, the larger percent of patients with higher score of that organ system, the border the ribbon. Phenotypes are shown in separate colors.

The other set of chord diagrams were created to visualize the distribution of nine most common admission diagnosis groups by phenotypes. These most common admission diagnosis groups vary from cohorts, including:

- - - Nonspecific chest pain
    - Abdominal pain
    - Complication of device; implant or graft
    - Other and unspecific lower respiratory disease
    - Septicemia (except in labor)
    - Acute cerebrovascular disease
    - Cardiac dysrhythmias.
    - Congestive heart failure; nonhypertensive
    - Malaise and fatigue
    - Osteoarthritis
    - Other complications of pregnancy

For each phenotype, the larger percentage of patients with that admission diagnosis group, the border the ribbon. Phenotypes are shown in separate colors. Diagrams were generated with Circlize R package[12].

- - Alluvial plots

Alluvial plots were generated to visualize distribution of phenotypes across worst Sequential Organ Failure Assessment Score (SOFA) scores of patients within first 24 hours of admission. Phenotypes were grouped in the left column and the total SOFA scores were categorized into 3 levels (0-1, 2-4 and 5+) listed in the right column. Ribbons connect the phenotypes and SOFA categories, which indicates a percentage of patients in a phenotype fall into a particular SOFA category and vice versa. The larger percentage of patients, the boarder the ribbon. Phenotypes are shown in separate colors. Plots were generated with Alluvial R package[13].

- - t-SNE plots

t-Distributed Stochastic Neighbor Embedding (t-SNE) is a nonlinear dimensionality reduction technique well-suited for embedding high-dimensional data for visualization in a low-dimensional space. In our work, the t-SNE plots depicted the 2 dimensional feature space of the patients vital signs after reducing their original dimension from 36 to 2 by t-SNE algorithm. Each dot represents a patient, and patients in different phenotypes are colored differently. Plots were generated by scikit-learn t-SNE Python package[14].

- - Mosaic plots

Mosaic plots were created to visualize the average vital patterns across phenotypes. These plots were created using the Gene Expression Dynamics Inspector (GEDI) software[15]. The algorithm that creates the vital mosaic is a self-organizing map. For each phenotype, we randomly picked 5% samples of that phenotype to calculate the average vital pattern due to the limitation of the software for big datasets. Patterns of individual samples of each phenotype were also created.

- - Line plots

Line plots were generated to visualize the time-series vital sign data as it is well-suited for analyzing trends of different variables along time. We created a line plot for each vital sign studied in our work. To better observe the trends of different vital signs, for each encounter, we resampled the raw time series data to 5 minute frequency by averaging multiple measurements every 5 minutes. Then line plots of phenotypes for each vital sign were created by plotting the mean value and 95% confidence interval around the mean. The six vital signs include:

- - - Systolic blood pressure
    - Diastolic blood pressure
    - Heart rate
    - Temperature
    - Blood oxygen saturation
    - Respiratory rate

Phenotypes are shown in separate colors. Plots were created by Seaborn lineplot Python package[16].

1. **Predicting cluster members in new datasets**

In the testing cohort, we used a prospective approach to assign phenotype membership to subject based upon clinical characteristics of typical cluster members in the training cohort.

To accomplish this, we first preprocessed the data using the procedure above (B). We then predicted phenotype assignments by calculating the Euclidean distance from each testing cohort admission to the centroid of each phenotype from training cohort. Consider the *i*th subject with *p* features. We represent it as $X_{i}=[x_{i1}, x_{i2}, \cdots, x_{ip}]$. We denote the mean of the *k*th phenotype with $\mu_{k}=[\mu_{k1}, \mu_{k2},\cdots,\mu_{kp}]$ and represent it as the center of the phenotype. Thus, we calculate the Euclidean distance of the *i*th admission to the center of the *k*th phenotype, $d_{i, k}$ as:

$$d_{i, k}= \sqrt{\sum_{j=1}^{p} {(x_{ij-}\mu_{kj})}^{2}}$$

We calculate distances of all admissions to all phenotype centroids and assigned each admission to its nearest phenotype. All phenotype centroids are listed in eTable 15.

1. **Definition of clinical characteristics**

Chronic disease burden was characterized by Charlson-Deyo comorbidity index scores.[17] Chronic kidney disease was determined from medical histories obtained prospectively at the time of enrollment and from a validated combination of International Classification of Diseases codes from electronic health records[18]. Severity of illness was characterized by SOFA and Modified Early Warning Score (MEWS) based on worst values within first 24 hours of hospital admission[19]. Missing SOFA and MEWS scores were imputed with 0.

Measurements for clinical biomarkers that fell outside of expert-defined ranges were considered outliers and were removed from the data. All measurements within 24 hours of hospital admission were used to detect highest or lowest value. Ranges of outliers and directionality of worst values are listed in eTable 2. Only results among patients with measurements were reported. We presented continuous variables as mean (SD) and median values with interquartile ranges and as frequencies and percentages for categorical variables.

For blood pressure, invasive measurements were used, and in absence of invasive measurements at a specific date and timestamp, noninvasive measurements were used. Duration of blood pressure below certain cutoff was determined in minutes after forward-propagating previous values. We identified number of pressors and need for inotrope in the first 24 hours of admission based on medications file where dopamine, droxidopa, midodrine, ephpedrine, epinephrine, norepinephrine, phenylephrine, and vasopressin were considered for vasopressors and dobutamine and milrinone for inotrope. Troponin measurements includes Troponin T and Troponin I. In order to determine FiO2 value at each date and time stamp, formulas were used to imputed FiO2 from oxygen delivery device and corresponding oxygen flow rate.[19] If no oxygen flow rate is given, default FiO2 was imputed based on respiratory device. If oxygen flow rate is outside specified range, minimum and maximum flow rate were used for imputing FiO2. If formula result is greater than maximum per-device FiO2, the maximum FiO2 was imputed. In absence of PaO2 to calculate PaO2/FiO2 ratio, SpO2/FiO2 to PaO2/FiO2 conversion was used[19, 20].

To determine reference creatinine, we used previously validated modification of the NHS England alert algorithm.[21] For patients with available preadmission measurements, reference value was defined as either the lowest in the last 7 days or a median of values from the preceding 8 to 365 days depending on availability of previous results. For patients with no available preadmission measurements and no history of chronic kidney disease (CKD) we used the lowest of admission creatinine and estimated baseline creatinine using the Modification of Diet in Renal Disease Study equation assuming that baseline estimated glomerular filtration rate (eGFR) is 75 ml/min per 1.73 m2. For patients with known history of CKD and no available preadmission measurements we used lowest creatinine value on admission day. After first seven days of hospitalization, minimum serum creatinine measurements in preceding 7 days was used as the reference creatinine. Reference creatinine was used to estimate preadmission reference glomerular filtration rate using Chronic Kidney Disease Epidemiology Collaboration equation.[22] Chronic kidney disease was determined from medical histories obtained prospectively at the time of enrollment and from a validated combination of International Classification of Diseases codes from electronic health records.[22] Chronic kidney disease stages were determined based on reference eGFR according to guidelines[23, 24].

**Diagnosis codes**

We determined category of admission diagnosis codes, which are assigned either as International Classification of Diseases, 9th Revision, Clinical Modification (ICD-9-CM) or International Classification of Diseases, 10th Revision, Clinical Modification (ICD-10-CM) code. We used general equivalence mappings to assist with the conversion ICD-10-CM codes to ICD-9-CM codes[25]. The Clinical Classification Software (CCS)[26] consists of two related classification systems, single-level and multi-level, which are designed to meet different needs. We used multi-level CCS which expands the single-level CCS into a hierarchical system and enables evaluating larger aggregations of conditions and procedures or exploring them in greater detail. The multi-level system has four levels for diagnoses and three levels for procedures, which provide the opportunity to examine general groupings or to assess very specific conditions and procedures. We showed distribution of most common Level 1 and Level 2 codes for each cluster as well as distribution of all admission diagnosis codes that are present in at least 1% proportion of patients.

1. **Definition of clinical outcomes**

We determined complications occurring anytime during hospitalization, including infectious and mechanical wound complications (wound complications), acute kidney injury (AKI), mechanical ventilation (MV) and intensive care unit (ICU) admission for greater than 48 hours, cardiovascular (CV) complications, neurological complications and/or delirium, sepsis, and venous thromboembolism (VTE). We used the exact dates and times to calculate the duration of MV, ICU, and hospital stay. In order to determine the duration of invasive mechanical ventilation, we developed an algorithm to identify the start and stop times for ventilation based on flowsheet data. Patient was determined to be on mechanical ventilation at a time point if the respiratory device is recorded as ventilator or endotracheal tube (ETT) or there is a recorded measurement value for tidal volume, end-tidal carbondioxide (etCO2), positive end-expiratory pressure (PEEP), mechanical respiratory rate, or ventilator mode. We identified need for pressors or inotropes (dobutamine, dopamine, droxidopa, midodrine, milrinone, ephpedrine, epinephrine, norepinephrine, phenylephrine, or vasopressin) during hospitalization based on detailed medication records data as binary variable. Acute kidney injury (AKI) was determined using available clinical information according to Kidney Disease: Improving Global Outcomes criteria (0.3 mg/dl increase in serum creatinine within 48 hours or 50% increase from baseline within seven days or decrease in urine output to less than 0.5 ml/kg/hr for six hours).[23] Community-acquired AKI was defined as development of AKI within 24 hours of hospital admission. Delirium was defined as at least one positive Confusion Assessment Method (CAM) score or having ICD-9 or ICD-10 codes for delirium. The International Classification of Diseases, Ninth and Tenth Revision, Clinical Modification (ICD-9-CM, ICD-10-CM) were used to the remaining complications[27-31]. Date of death was determined using hospital records and the Social Security Death Index database was used to confirm death dates and obtain death dates for subjects who were not in hospital records. Thirty-day and three-year mortality were defined if the death date is thirty days or three year from hospital admission.

# References

1. Booth HP, Prevost AT, Gulliford MC. Validity of smoking prevalence estimates from primary care electronic health records compared with national population survey data for England, 2007 to 2011. Pharmacoepidemiol Drug Saf. 2013;22(12):1357-61. Epub 2013/11/19. doi: 10.1002/pds.3537. PubMed PMID: 24243711.

2. Monti S, Tamayo P, Mesirov J, Golub T. Consensus Clustering: A Resampling-Based Method for Class Discovery and Visualization of Gene Expression Microarray Data. Mach Learn. 2003;52(1–2):91–118. doi: 10.1023/a:1023949509487.

3. Seymour CW, Kennedy JN, Wang S, Chang CH, Elliott CF, Xu Z, et al. Derivation, Validation, and Potential Treatment Implications of Novel Clinical Phenotypes for Sepsis. Jama. 2019;321(20):2003-17. Epub 2019/05/20. doi: 10.1001/jama.2019.5791. PubMed PMID: 31104070; PubMed Central PMCID: PMCPMC6537818.

4. Sweeney TE, Azad TD, Donato M, Haynes WA, Perumal TM, Henao R, et al. Unsupervised Analysis of Transcriptomics in Bacterial Sepsis Across Multiple Datasets Reveals Three Robust Clusters. Crit Care Med. 2018;46(6):915-25. Epub 2018/03/15. doi: 10.1097/ccm.0000000000003084. PubMed PMID: 29537985; PubMed Central PMCID: PMCPMC5953807.

5. Vranas KC, Jopling JK, Sweeney TE, Ramsey MC, Milstein AS, Slatore CG, et al. Identifying Distinct Subgroups of ICU Patients: A Machine Learning Approach. Crit Care Med. 2017;45(10):1607-15. Epub 2017/06/24. doi: 10.1097/ccm.0000000000002548. PubMed PMID: 28640021; PubMed Central PMCID: PMCPMC5600667.

6. Swift S, Tucker A, Vinciotti V, Martin N, Orengo C, Liu X, et al. Consensus clustering and functional interpretation of gene-expression data. Genome Biol. 2004;5(11):R94. Epub 2004/11/13. doi: 10.1186/gb-2004-5-11-r94. PubMed PMID: 15535870; PubMed Central PMCID: PMCPMC545785.

7. Topchy A, Jain A, Punch W. Clustering ensembles: models of consensus and weak partitions. IEEE Transactions on Pattern Analysis and Machine Intelligence. 2005;27(12):1866-81. doi: 10.1109/TPAMI.2005.237.

8. Gionis A, Mannila H, Tsaparas P. Clustering aggregation. ACM Trans Knowl Discov Data. 2007;1(1):4–es. doi: 10.1145/1217299.1217303.

9. Strehl A, Ghosh J. Cluster ensembles --- a knowledge reuse framework for combining multiple partitions. J Mach Learn Res. 2003;3(null):583–617. doi: 10.1162/153244303321897735.

10. Fraley A, Raftery A. How Many Clusters? Which Clustering Method? Answers Via Model-Based Cluster Analysis. The Computer Journal. 1998;41(8):578-88. doi: 10.1093/comjnl/41.8.578.

11. Scrucca L, Fop M, Murphy TB, Raftery AE. mclust 5: Clustering, Classification and Density Estimation Using Gaussian Finite Mixture Models. R j. 2016;8(1):289-317. Epub 2016/11/08. PubMed PMID: 27818791; PubMed Central PMCID: PMCPMC5096736.

12. Gu Z, Gu L, Eils R, Schlesner M, Brors B. circlize Implements and enhances circular visualization in R. Bioinformatics. 2014;30(19):2811-2. Epub 2014/06/16. doi: 10.1093/bioinformatics/btu393. PubMed PMID: 24930139.

13. Alluvial: R Package for Creating Alluvial Diagrams. Version: 0.1-2. Bojanowski M and Edwards R [08/19/2020]. Available from: <https://github.com/mbojan/alluvial>.

14. scikit-learn t-SNE Python package [cited 2020 08/19/2020]. Available from: <https://scikit-learn.org/stable/modules/generated/sklearn.manifold.TSNE.html>.

15. Eichler GS, Huang S, Ingber DE. Gene Expression Dynamics Inspector (GEDI): for integrative analysis of expression profiles. Bioinformatics. 2003;19(17):2321-2. Epub 2003/11/25. doi: 10.1093/bioinformatics/btg307. PubMed PMID: 14630665.

16. Seaborn lineplot Python package [08/19/2020]. Available from: <https://seaborn.pydata.org/generated/seaborn.lineplot.html>.

17. Deyo RA, Cherkin DC, Ciol MA. Adapting a clinical comorbidity index for use with ICD-9-CM administrative databases. J Clin Epidemiol. 1992;45(6):613-9. Epub 1992/06/01. doi: 10.1016/0895-4356(92)90133-8. PubMed PMID: 1607900.

18. Wald R, Waikar SS, Liangos O, Pereira BJ, Chertow GM, Jaber BL. Acute renal failure after endovascular vs open repair of abdominal aortic aneurysm. J Vasc Surg. 2006;43(3):460-6; discussion 6. Epub 2006/03/08. doi: 10.1016/j.jvs.2005.11.053. PubMed PMID: 16520155.

19. Shickel B, Loftus TJ, Adhikari L, Ozrazgat-Baslanti T, Bihorac A, Rashidi P. DeepSOFA: A Continuous Acuity Score for Critically Ill Patients using Clinically Interpretable Deep Learning. Sci Rep. 2019;9(1):1879. Epub 2019/02/14. doi: 10.1038/s41598-019-38491-0. PubMed PMID: 30755689; PubMed Central PMCID: PMCPMC6372608.

20. Rice TW, Wheeler AP, Bernard GR, Hayden DL, Schoenfeld DA, Ware LB. Comparison of the SpO2/FIO2 ratio and the PaO2/FIO2 ratio in patients with acute lung injury or ARDS. Chest. 2007;132(2):410-7. Epub 2007/06/19. doi: 10.1378/chest.07-0617. PubMed PMID: 17573487.

21. Selby NM, Hill R, Fluck RJ. Standardizing the Early Identification of Acute Kidney Injury: The NHS England National Patient Safety Alert. Nephron. 2015;131(2):113-7. Epub 2015/09/10. doi: 10.1159/000439146. PubMed PMID: 26351847.

22. Levey AS, Stevens LA, Schmid CH, Zhang YL, Castro AF, 3rd, Feldman HI, et al. A new equation to estimate glomerular filtration rate. Ann Intern Med. 2009;150(9):604-12. Epub 2009/05/06. doi: 10.7326/0003-4819-150-9-200905050-00006. PubMed PMID: 19414839; PubMed Central PMCID: PMCPMC2763564.

23. Kidney Disease: Improving Global Outcomes (KDIGO) CKD Work Group. KDIGO 2012 clinical practice guideline for the evaluation and management of chronic kidney disease. Kidney Int, Suppl. 2013;3(1):1-150.

24. Stevens P, Levin A. Kidney Disease: Improving Global Outcomes Chronic Kidney Disease Guideline Development Work Group M. Evaluation and management of chronic kidney disease: synopsis of the kidney disease: improving global outcomes 2012 clinical practice guideline. Ann Intern Med. 2013;158(11):825-30.

25. General equivalence mappings to assist with the conversion ICD-10-CM codes to ICD-9-CM codes [08/19/2020]. Available from: <https://www.cms.gov/Medicare/Coding/ICD10/downloads/ICD-10_GEM_fact_sheet.pdf>.

26. Clinical Classification Software (CCS) [08/19/2020]. Available from: <https://www.hcup-us.ahrq.gov/db/vars/dxmccsn/nisnote.jsp>.

27. LaPar DJ, Bhamidipati CM, Mery CM, Stukenborg GJ, Jones DR, Schirmer BD, et al. Primary payer status affects mortality for major surgical operations. Ann Surg. 2010;252(3):544-50; discussion 50-1. Epub 2010/07/22. doi: 10.1097/SLA.0b013e3181e8fd75. PubMed PMID: 20647910; PubMed Central PMCID: PMCPMC3071622.

28. Guller U, Hervey S, Purves H, Muhlbaier LH, Peterson ED, Eubanks S, et al. Laparoscopic versus open appendectomy: outcomes comparison based on a large administrative database. Ann Surg. 2004;239(1):43-52. Epub 2003/12/20. doi: 10.1097/01.sla.0000103071.35986.c1. PubMed PMID: 14685099; PubMed Central PMCID: PMCPMC1356191.

29. Dombrovskiy VY, Martin AA, Sunderram J, Paz HL. Rapid increase in hospitalization and mortality rates for severe sepsis in the United States: a trend analysis from 1993 to 2003. Crit Care Med. 2007;35(5):1244-50. Epub 2007/04/07. doi: 10.1097/01.Ccm.0000261890.41311.E9. PubMed PMID: 17414736.

30. Thottakkara P, Ozrazgat-Baslanti T, Hupf BB, Rashidi P, Pardalos P, Momcilovic P, et al. Application of Machine Learning Techniques to High-Dimensional Clinical Data to Forecast Postoperative Complications. PLoS One. 2016;11(5):e0155705. Epub 2016/05/28. doi: 10.1371/journal.pone.0155705. PubMed PMID: 27232332; PubMed Central PMCID: PMCPMC4883761.

31. Hobson C, Ozrazgat-Baslanti T, Kuxhausen A, Thottakkara P, Efron PA, Moore FA, et al. Cost and Mortality Associated With Postoperative Acute Kidney Injury. Ann Surg. 2015;261(6):1207-14. Epub 2014/06/03. doi: 10.1097/sla.0000000000000732. PubMed PMID: 24887982; PubMed Central PMCID: PMCPMC4247993.
