# Supplementary figures and images for: Physiologic signatures within six hours of hospitalization identify acute illness phenotypes
Source: PLOS Digit Health. 2022 Oct 13;1(10):e0000110. doi: 10.1371/journal.pdig.0000110 (PMC9802629; doi:10.1371/journal.pdig.0000110)

# S1 Fig. Cohort selection and exclusion criteria


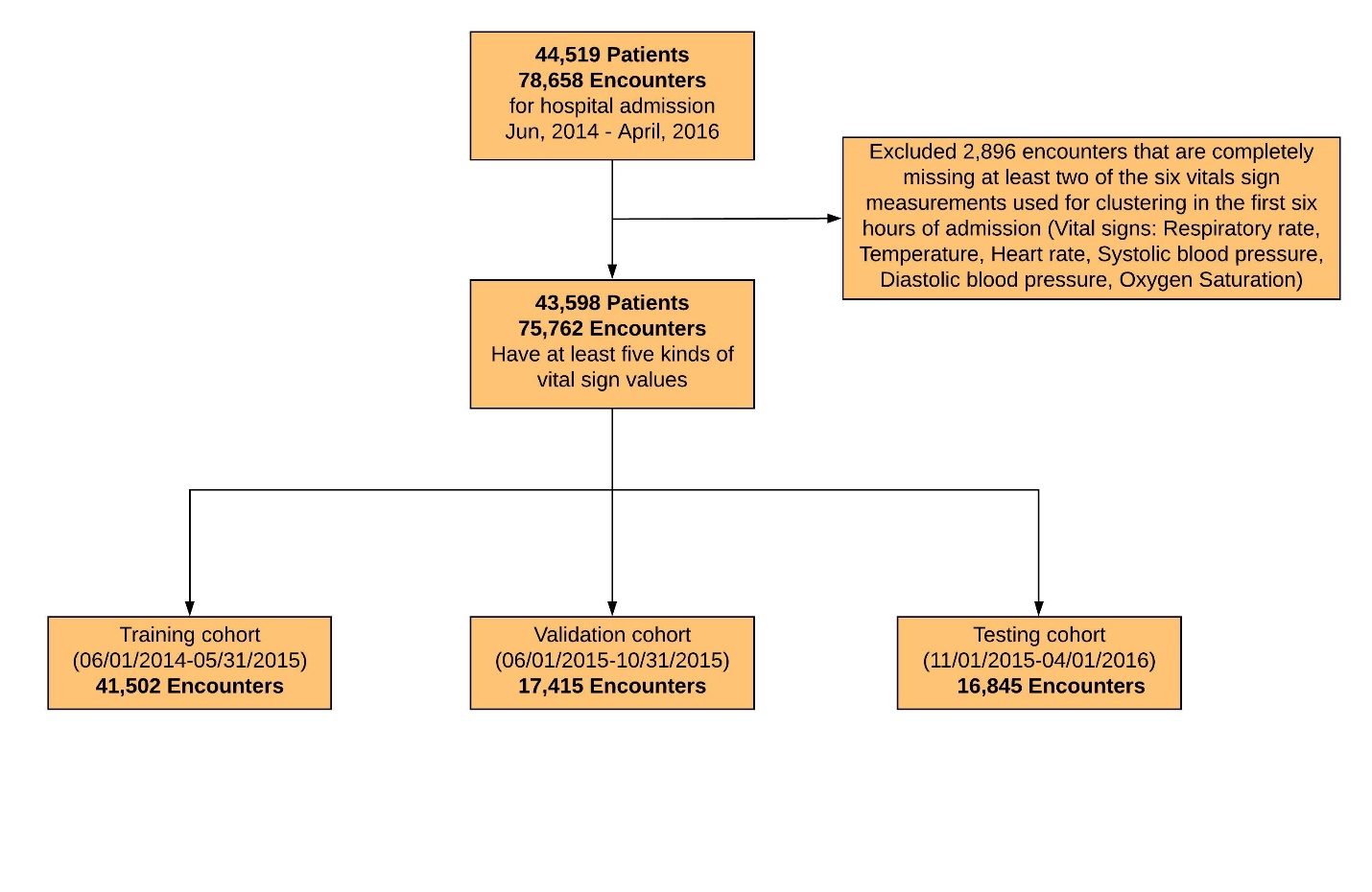

Supplement: S1 Fig — (DOCX) [file pdig.0000110.s002.docx]

# S2 Fig. Purposes of training, validation, and testing cohorts


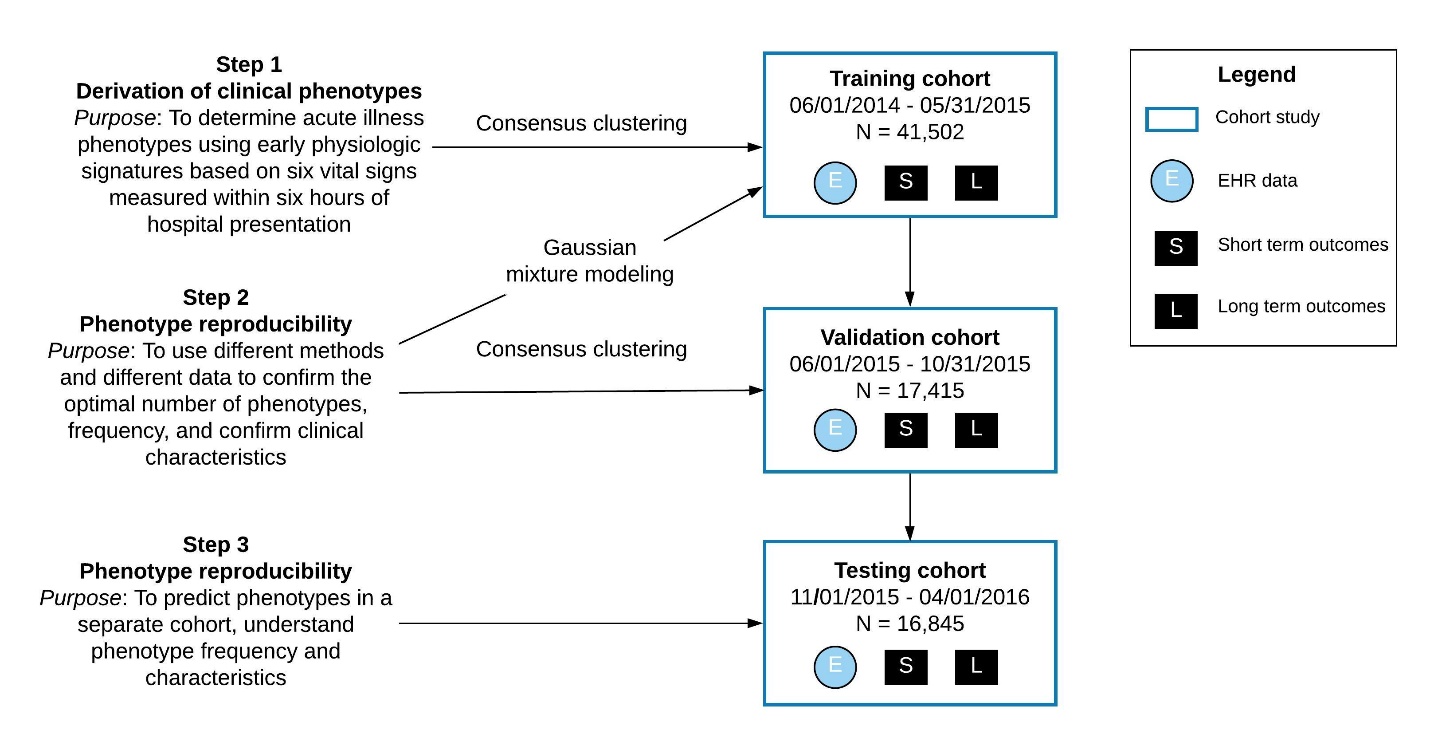

Supplement: S2 Fig — (DOCX) [file pdig.0000110.s003.docx]
