# Supplementary material for: Physiologic signatures within six hours of hospitalization identify acute illness phenotypes
Source: PLOS Digit Health. 2022 Oct 13;1(10):e0000110. doi: 10.1371/journal.pdig.0000110 (PMC9802629; doi:10.1371/journal.pdig.0000110)
Supplement: S4 Fig — Spearman correlation heat map shows the pairwise spearman rank order correlation coefficient among the 6 vital signs studied in our paper. The darker red color, the higher correlation in positive direction. Abbreviations: RR: respiratory rate; SpO2: peripheral capillary oxygen saturation; Temp: temperature; HR: heart rate; SBP: systolic blood pressure; DBP: diastolic blood pressure. (DOCX) [file pdig.0000110.s005.docx]

# S4 Fig. Spearman correlation heat map for the training cohort (N=41,502)


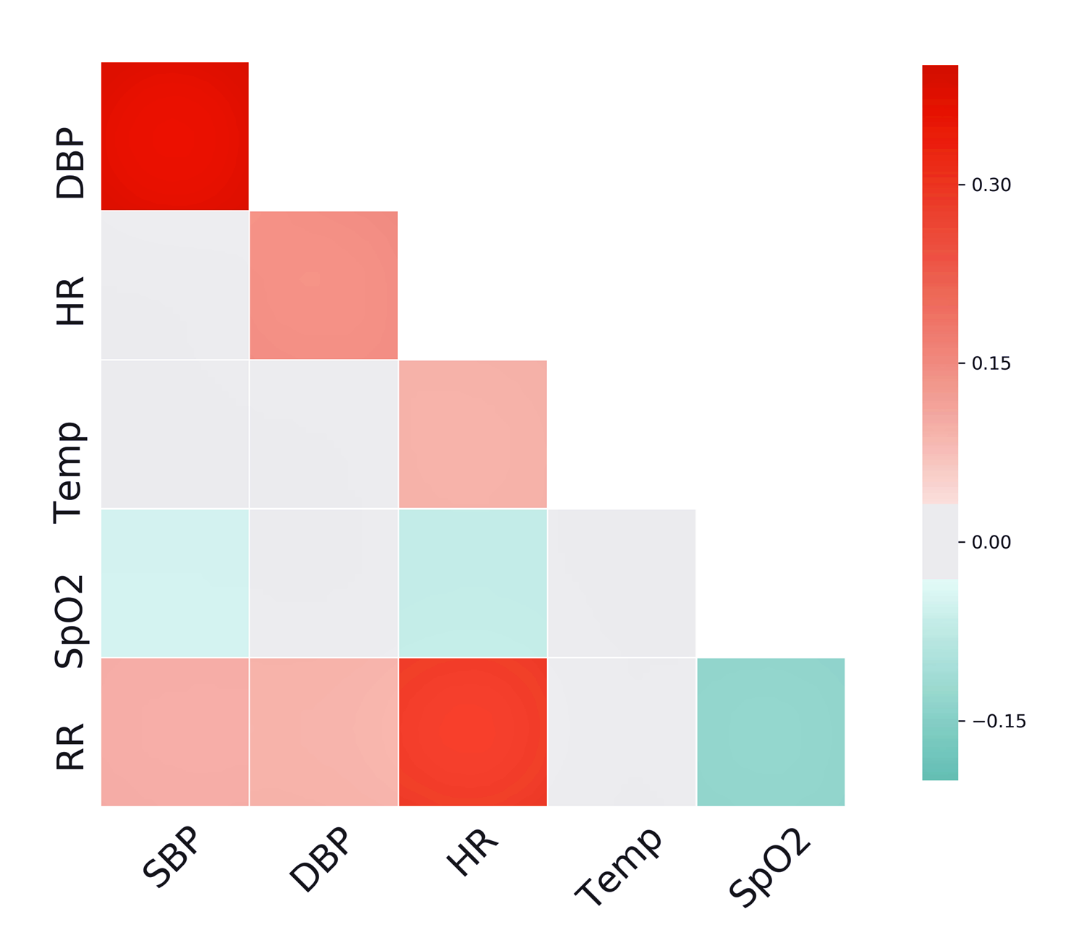


Spearman correlation heat map shows the pairwise spearman rank order correlation coefficient among the 6 vital signs studied in our paper. The darker red color, the higher correlation in positive direction.

Abbreviations: RR: respiratory rate; SpO2: peripheral capillary oxygen saturation; Temp: temperature; HR: heart rate; SBP: systolic blood pressure; DBP: diastolic blood pressure.
