# Supplementary material for: Physiologic signatures within six hours of hospitalization identify acute illness phenotypes
Source: PLOS Digit Health. 2022 Oct 13;1(10):e0000110. doi: 10.1371/journal.pdig.0000110 (PMC9802629; doi:10.1371/journal.pdig.0000110)
Supplement: S5 Fig — (DOCX) [file pdig.0000110.s006.docx]

# S5 Fig. Average vital sign mosaics of phenotypes using a self-organizing map in the training cohort (N = 41,502)

**Individual vital sign mosaic (example 2)**

**Individual vital sign mosaic (example 1)**

**Average vital sign mosaic**


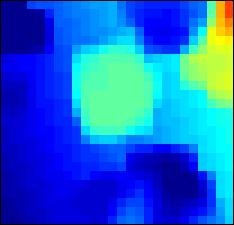

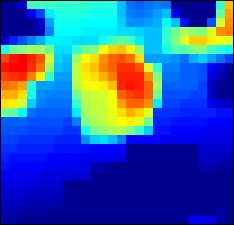

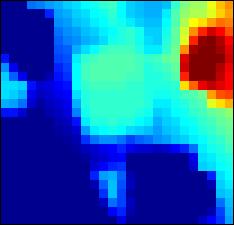


Physiotype A


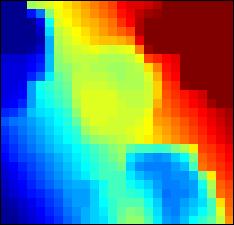

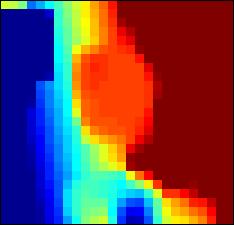

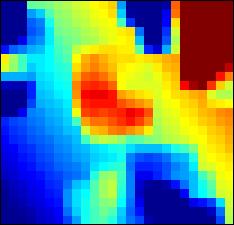


Physiotype B


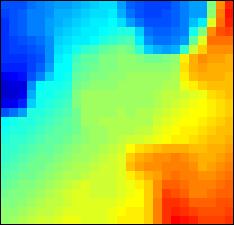

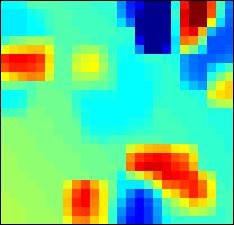

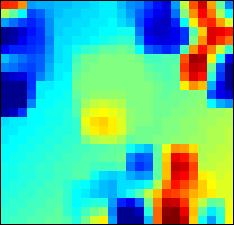


Physiotype C


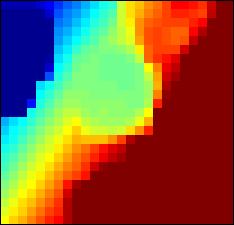

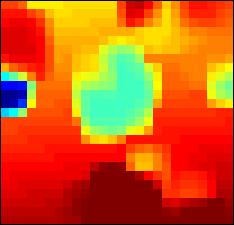

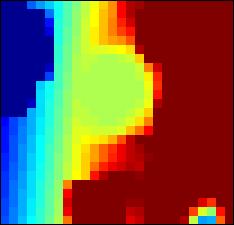


Physiotype D
