# Supplementary material for: Physiologic signatures within six hours of hospitalization identify acute illness phenotypes
Source: PLOS Digit Health. 2022 Oct 13;1(10):e0000110. doi: 10.1371/journal.pdig.0000110 (PMC9802629; doi:10.1371/journal.pdig.0000110)
Supplement: S8 Fig — (A) Physiotype survival curves adjusted using demographic information and comorbidities. (B) Adjusted Cox proportional hazards models using demographic information and comorbidities. (C) Physiotype survival curves adjusted using demographic information, comorbidities, and SOFA scores. (D) Adjusted Cox proportional hazards model using demographic information, comorbidities, and SOFA scores. Abbreviation: CCI: charlson comorbidity index; SOFA: sequential organ failure assessment. (DOCX) [file pdig.0000110.s009.docx]

# S8 Fig. Survival curves and Cox proportional hazards modeling by phenotypes in training cohort


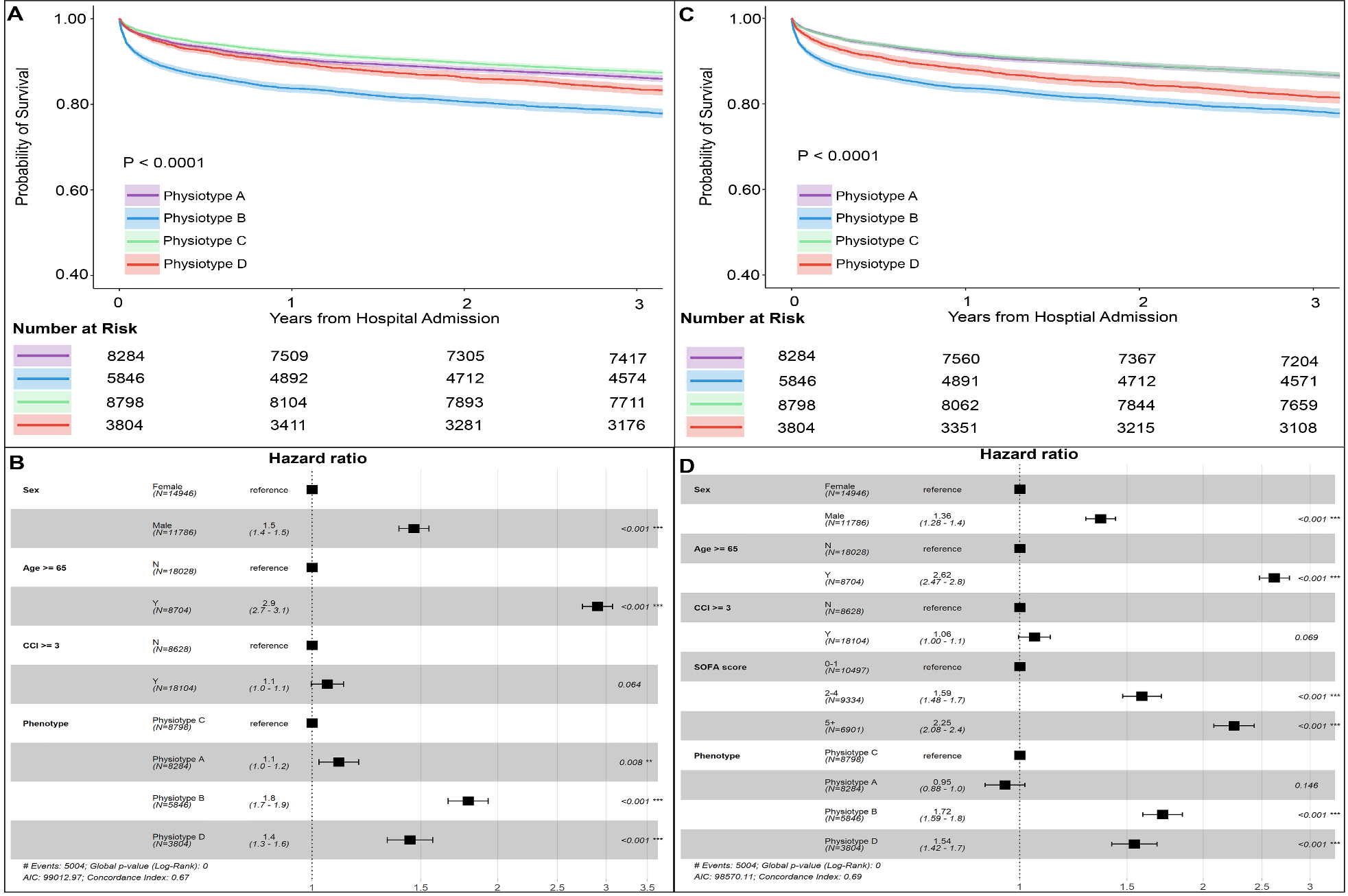


(A) Physiotype survival curves adjusted using demographic information and comorbidities. (B) Adjusted Cox proportional hazards models using demographic information and comorbidities. (C) Physiotype survival curves adjusted using demographic information, comorbidities, and SOFA scores. (D) Adjusted Cox proportional hazards model using demographic information, comorbidities, and SOFA scores. Abbreviation: CCI: charlson comorbidity index; SOFA: sequential organ failure assessment.
