# Supplementary material for: Physiologic signatures within six hours of hospitalization identify acute illness phenotypes
Source: PLOS Digit Health. 2022 Oct 13;1(10):e0000110. doi: 10.1371/journal.pdig.0000110 (PMC9802629; doi:10.1371/journal.pdig.0000110)
Supplement: S9 Fig — (A) Unsupervised consensus k clustering in training cohort showing optimal partitioning in consensus matrix for k = 4. (B) Consensus cumulative distribution function (CDF) across k = 2 to k = 8, where more horizontal curves suggest optimal fit. (C) Relative change in the area under the CDF curve with increasing clusters (k), with little change beyond k = 4. (D) Cluster consensus plot showing the mean of all pairwise consensus values between a cluster members, for k = 2 to k = 8 where greater values for all bars suggest optimal fit. (DOCX) [file pdig.0000110.s010.docx]

# S9 Fig. Consensus k clustering results in validation cohort (N=17,415)


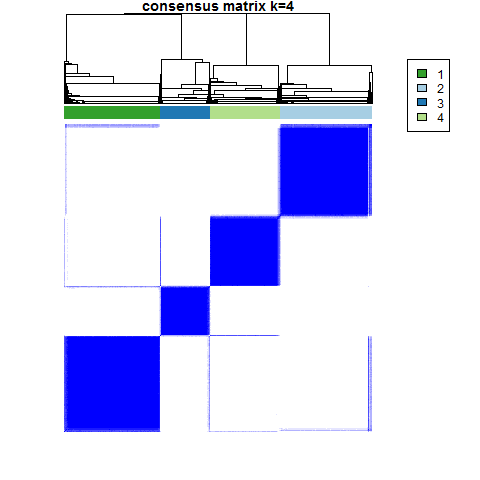

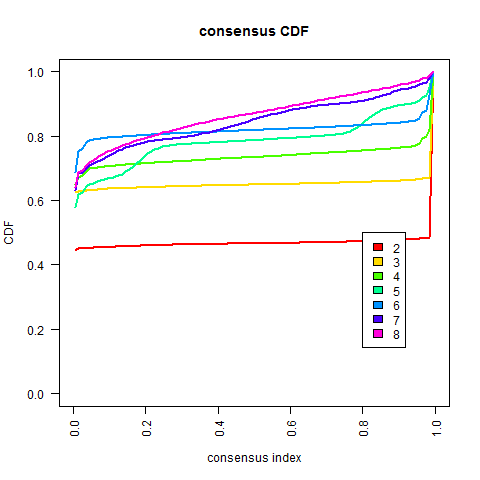


(A) (B)


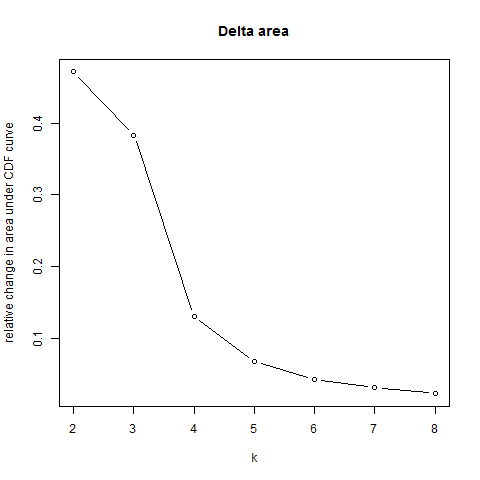

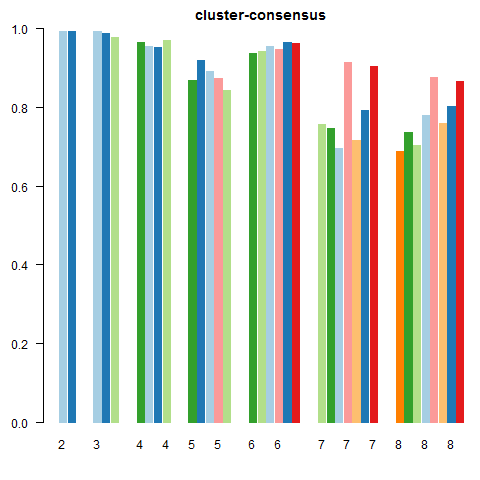

 (C) (D)

(A) Unsupervised consensus k clustering in training cohort showing optimal partitioning in consensus matrix for k = 4. (B) Consensus cumulative distribution function (CDF) across k = 2 to k = 8, where more horizontal curves suggest optimal fit. (C) Relative change in the area under the CDF curve with increasing clusters (k), with little change beyond k = 4. (D) Cluster consensus plot showing the mean of all pairwise consensus values between a cluster members, for k = 2 to k = 8 where greater values for all bars suggest optimal fit.
