# Supplementary material for: Physiologic signatures within six hours of hospitalization identify acute illness phenotypes
Source: PLOS Digit Health. 2022 Oct 13;1(10):e0000110. doi: 10.1371/journal.pdig.0000110 (PMC9802629; doi:10.1371/journal.pdig.0000110)
Supplement: S10 Fig — In all panels, the variables are standardized such that all means are scaled to 0 and SDs to 1. A value of 1 for the standardized variable (x-axis) signifies that the mean value for the phenotype was 1 SD higher than the mean value for both phenotypes shown in the graph as a whole. Abbreviations in order: SpO2: peripheral capillary oxygen saturation; Temp: temperature; SBP: systolic blood pressure; DBP: diastolic blood pressure, RR: respiratory rate; HR: heart rate. (DOCX) [file pdig.0000110.s011.docx]

# S10 Fig. Mean standardized differences between variables across phenotype pairs for training cohort (N = 41,502, dark line) and validation cohort (N = 17,415) using consensus clustering

**
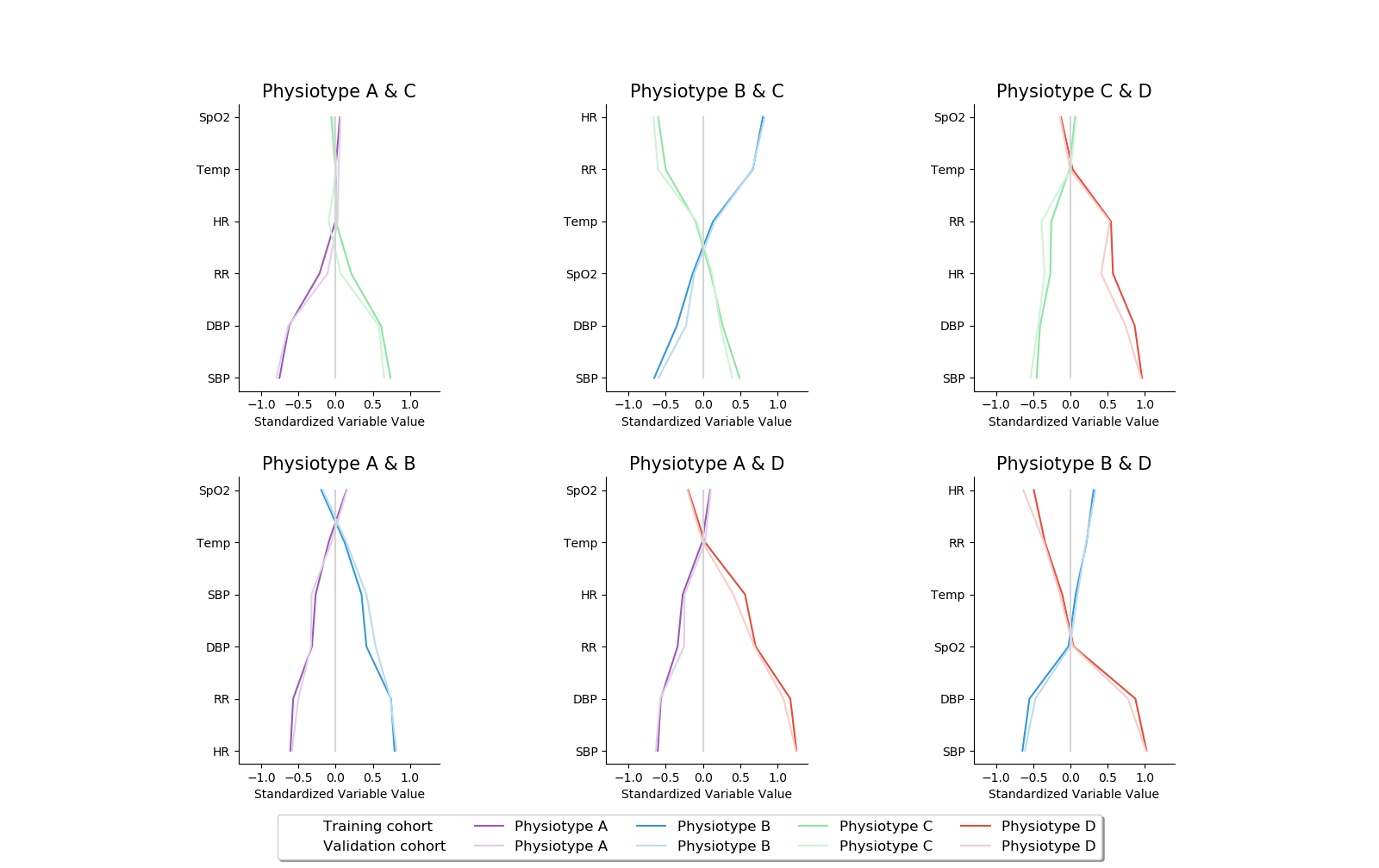
**

In all panels, the variables are standardized such that all means are scaled to 0 and SDs to 1. A value of 1 for the standardized variable (x-axis) signifies that the mean value for the phenotype was 1 SD higher than the mean value for both phenotypes shown in the graph as a whole.

Abbreviations in order: SpO2: peripheral capillary oxygen saturation; Temp: temperature; SBP: systolic blood pressure; DBP: diastolic blood pressure, RR: respiratory rate; HR: heart rate.
