# Supplementary material for: Physiologic signatures within six hours of hospitalization identify acute illness phenotypes
Source: PLOS Digit Health. 2022 Oct 13;1(10):e0000110. doi: 10.1371/journal.pdig.0000110 (PMC9802629; doi:10.1371/journal.pdig.0000110)

# S11 Fig. Validation cohort clusters had unique distributions of vital signs during the first six hours of admission


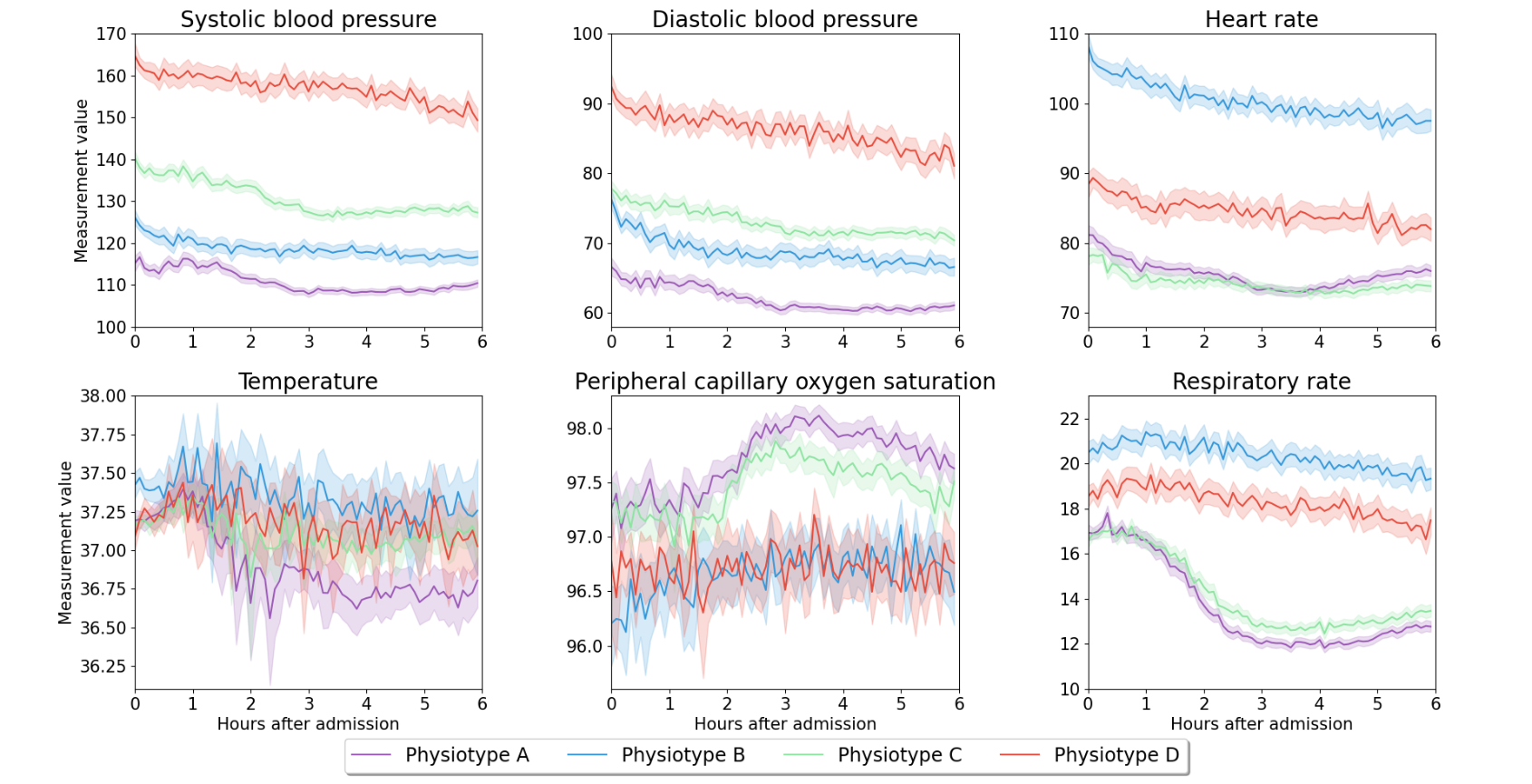

Supplement: S11 Fig — (DOCX) [file pdig.0000110.s012.docx]
