# Supplementary material for: Physiologic signatures within six hours of hospitalization identify acute illness phenotypes
Source: PLOS Digit Health. 2022 Oct 13;1(10):e0000110. doi: 10.1371/journal.pdig.0000110 (PMC9802629; doi:10.1371/journal.pdig.0000110)

# S15 Fig. Distribution of vital signs by phenotypes in sensitivity analysis using a 12-hour window of EHR data in the training cohort (N=41,502)


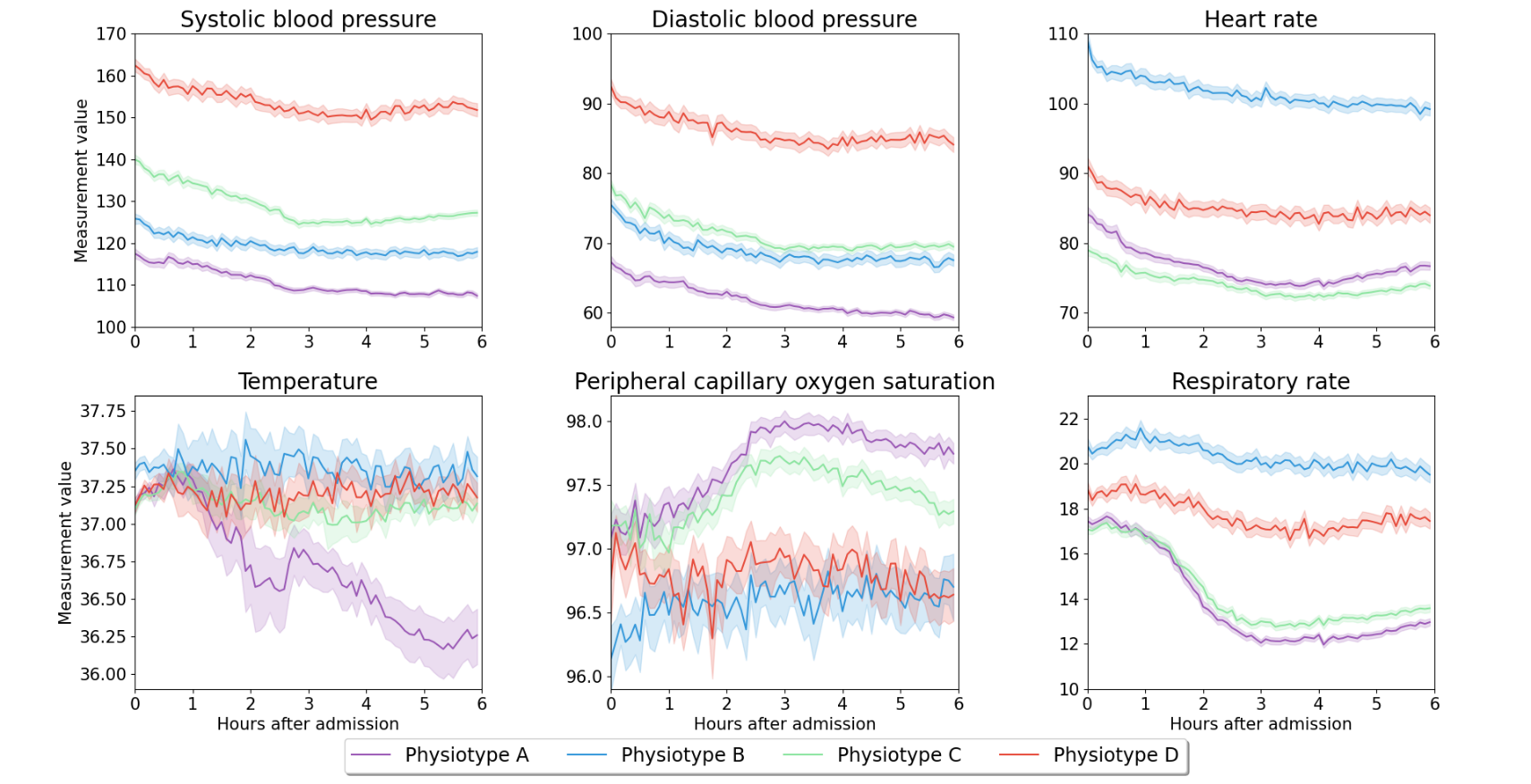

Supplement: S15 Fig — (DOCX) [file pdig.0000110.s016.docx]
