# Supplementary material for: Physiologic signatures within six hours of hospitalization identify acute illness phenotypes
Source: PLOS Digit Health. 2022 Oct 13;1(10):e0000110. doi: 10.1371/journal.pdig.0000110 (PMC9802629; doi:10.1371/journal.pdig.0000110)
Supplement: S17 Fig — Starting from the original 36 dimensional vital signs, we run the t-SNE to reduce to 2 dimensions. Each dot represents a patient. Phenotypes are shown in separate colors. (DOCX) [file pdig.0000110.s018.docx]

# S17 Fig. t-SNE plot of phenotype assignments in testing cohort


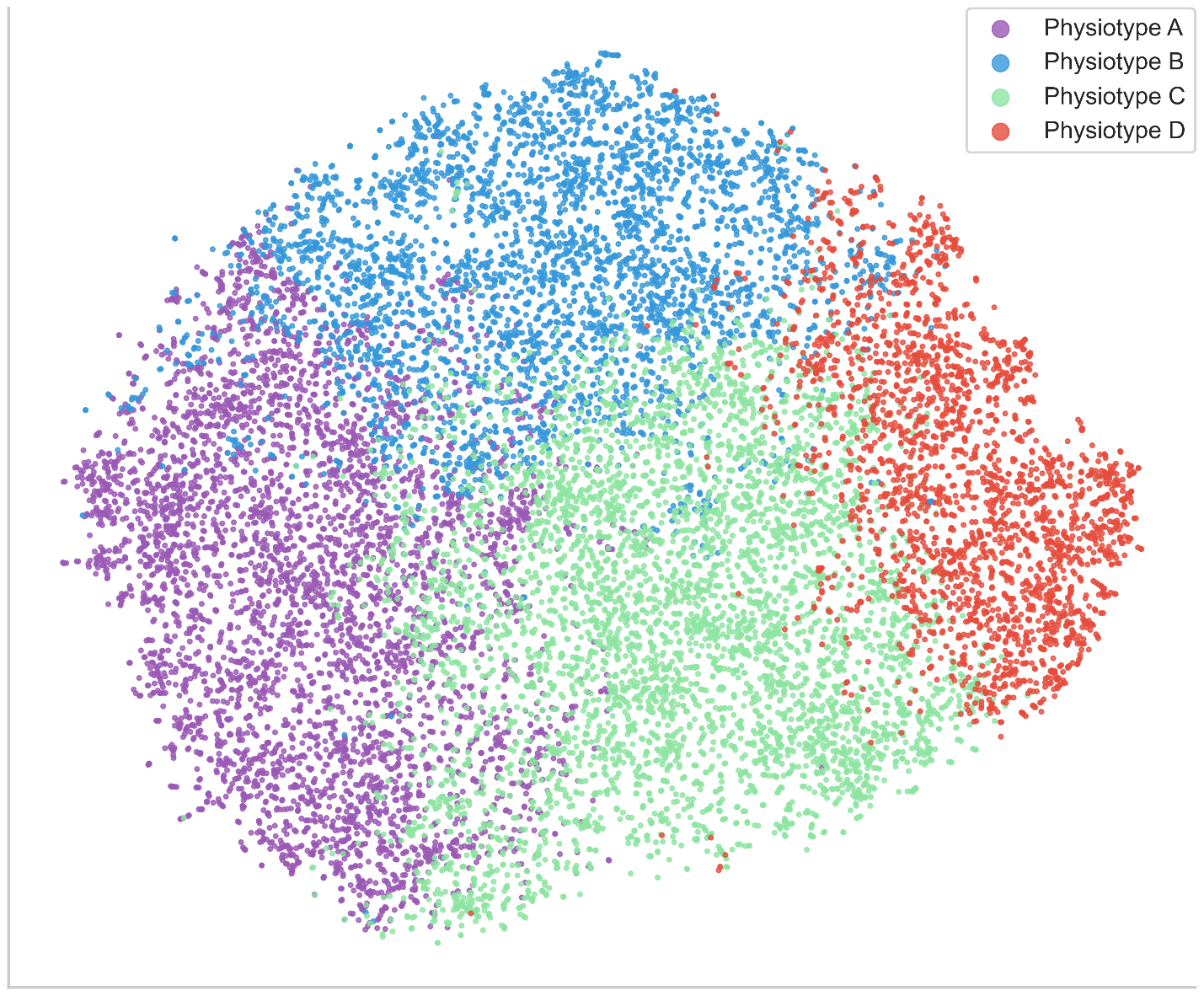


Starting from the original 36 dimensional vital signs, we run the t-SNE to reduce to 2 dimensions. Each dot represents a patient. Phenotypes are shown in separate colors.
