# Supplementary material for: Physiologic signatures within six hours of hospitalization identify acute illness phenotypes
Source: PLOS Digit Health. 2022 Oct 13;1(10):e0000110. doi: 10.1371/journal.pdig.0000110 (PMC9802629; doi:10.1371/journal.pdig.0000110)
Supplement: S19 Fig — For each phenotype, the larger percentage of patients with higher score of that organ system, the border the ribbon. (DOCX) [file pdig.0000110.s020.docx]

# S19 Fig. Chord diagrams showing the distribution of patients with higher SOFA scores (i.e., 2+) within first 24 hours of admission of six organ systems by phenotypes in validation cohort

(i) All physiotypes


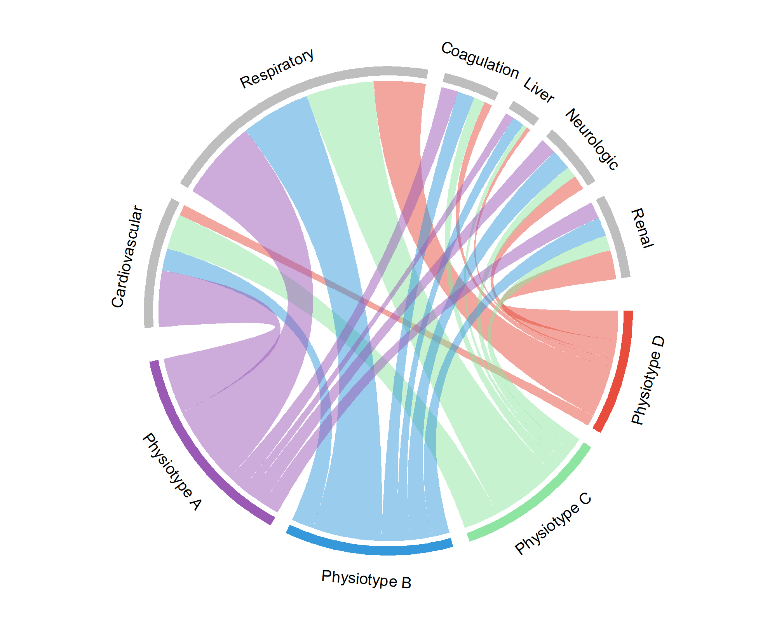


(iii) Physiotype B


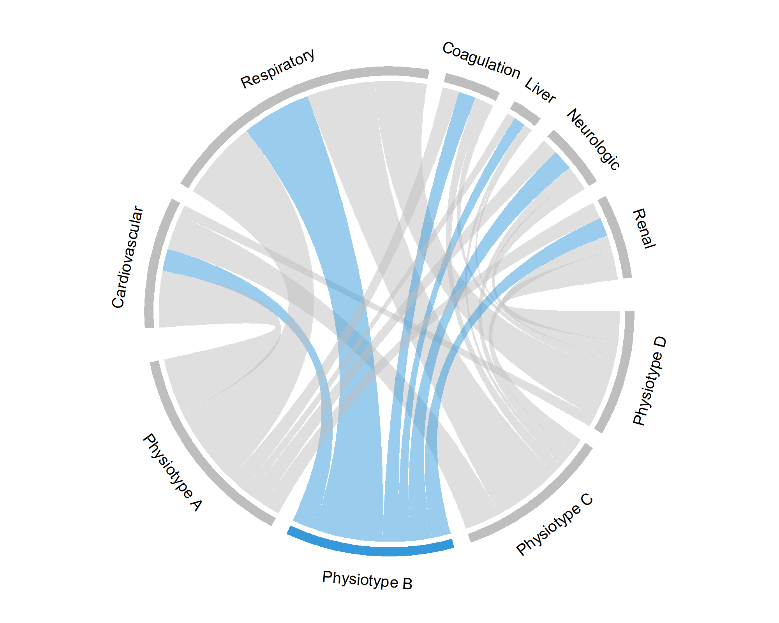


(ii) Physiotype A


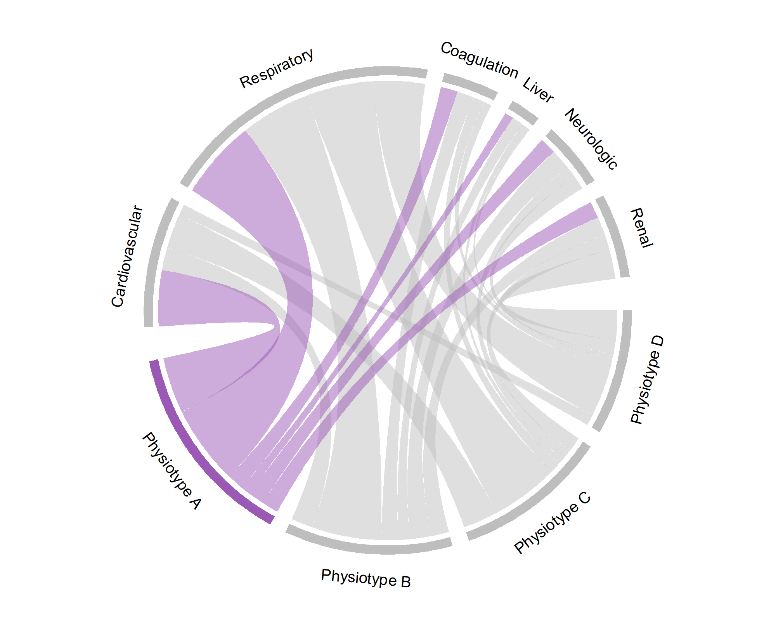


(iv) Physiotype C


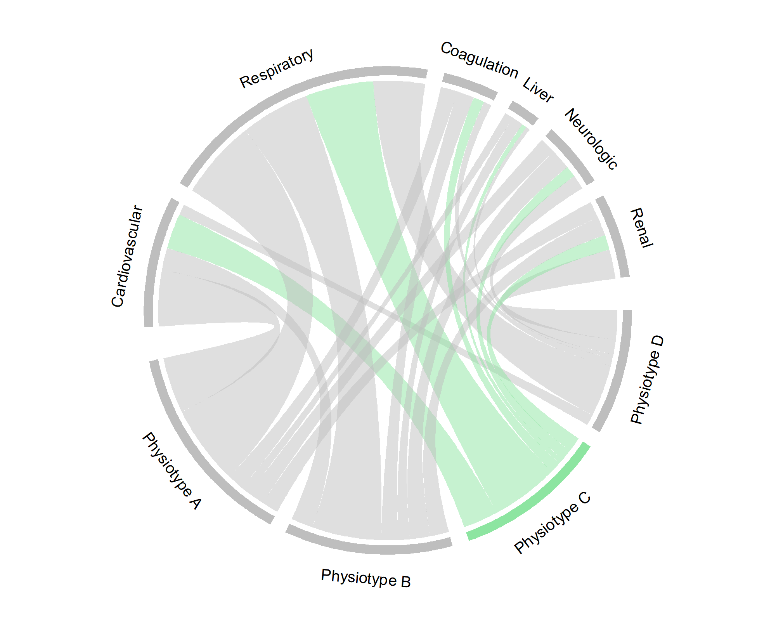


(v) Physiotype D


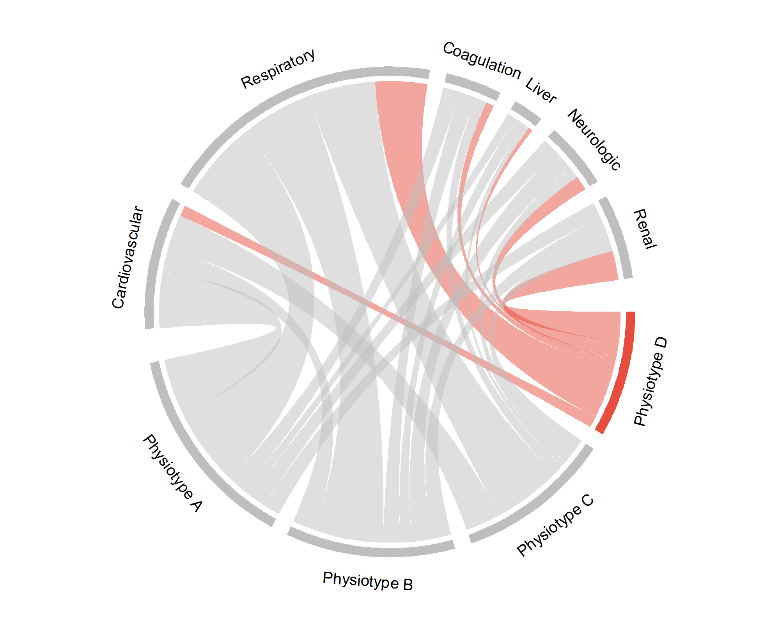


For each phenotype, the larger percentage of patients with higher score of that organ system, the border the ribbon.
