# Supplementary material for: Physiologic signatures within six hours of hospitalization identify acute illness phenotypes
Source: PLOS Digit Health. 2022 Oct 13;1(10):e0000110. doi: 10.1371/journal.pdig.0000110 (PMC9802629; doi:10.1371/journal.pdig.0000110)
Supplement: S20 Fig — For each phenotype, the larger percentage of patients with that score, the broader the ribbon. (DOCX) [file pdig.0000110.s021.docx]

# S20 Fig. Alluvial plot showing distribution of phenotypes across worst SOFA scores of patients within first 24 hours of admission in testing cohort


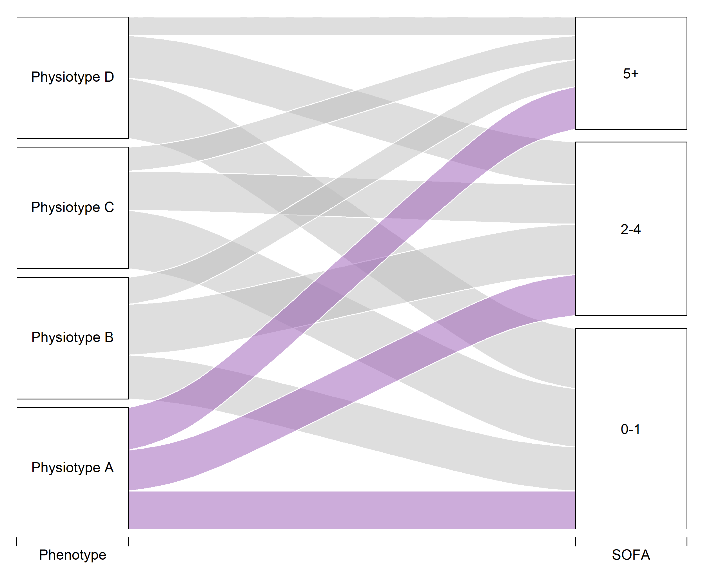

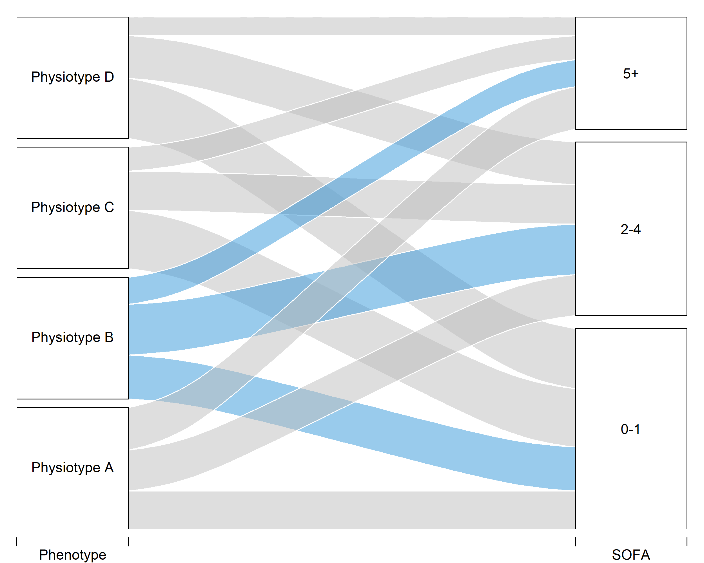


(A) Physiotype A (B) Physiotype B


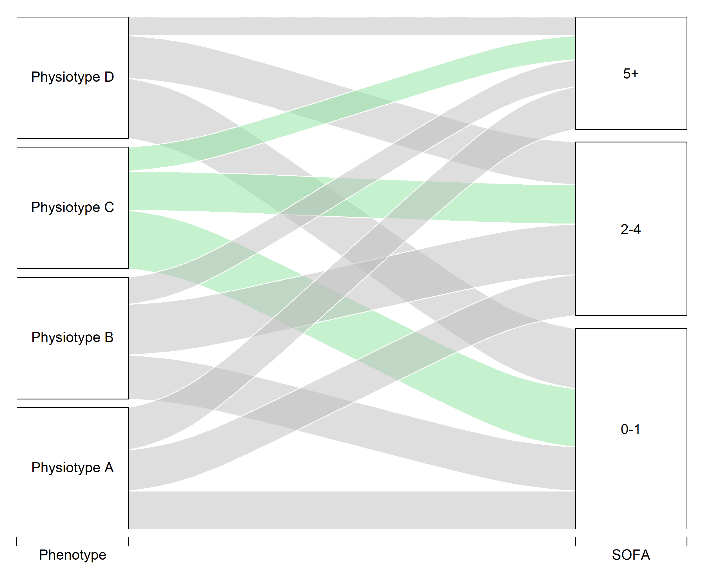

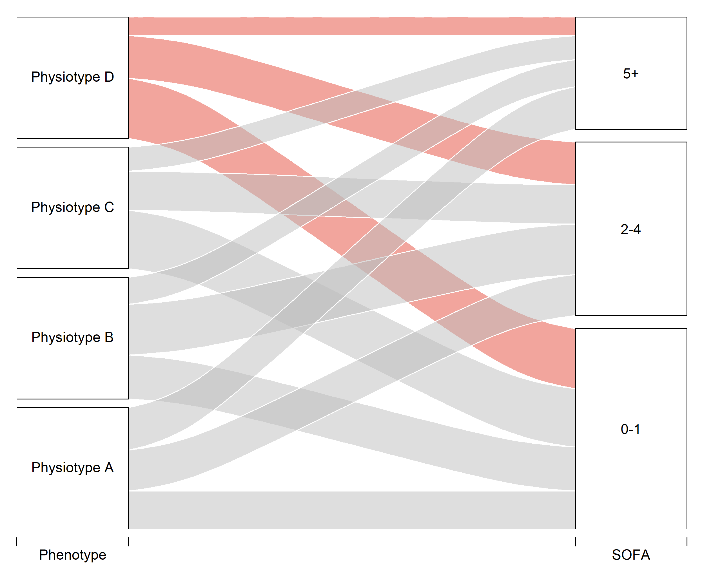


(C) Physiotype C (D) Physiotype D

For each phenotype, the larger percentage of patients with that score, the broader the ribbon.
