# Supplementary material for: Physiologic signatures within six hours of hospitalization identify acute illness phenotypes
Source: PLOS Digit Health. 2022 Oct 13;1(10):e0000110. doi: 10.1371/journal.pdig.0000110 (PMC9802629; doi:10.1371/journal.pdig.0000110)
Supplement: S23 Fig — (A) Physiotype survival curves adjusted using demographic information and comorbidities. (B) Adjusted Cox proportional hazards models using demographic information and comorbidities. (C) Physiotype survival curves adjusted using demographic information, comorbidities, and SOFA scores. (D) Adjusted Cox proportional hazards model using demographic information, comorbidities, and SOFA scores. Abbreviation: CCI: charlson comorbidity index; SOFA: sequential organ failure assessment. (DOCX) [file pdig.0000110.s024.docx]

# S23 Fig. Survival curves and Cox proportional hazards modeling by phenotypes in testing cohort


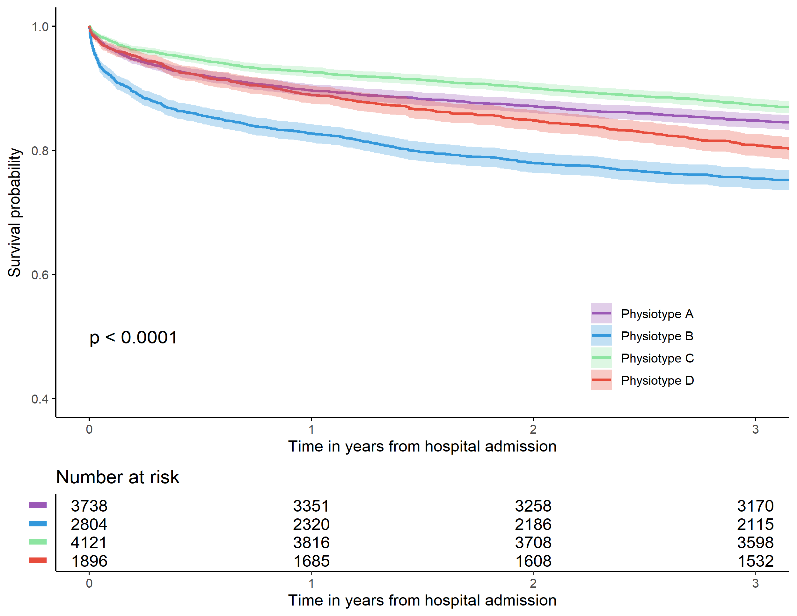


(A)


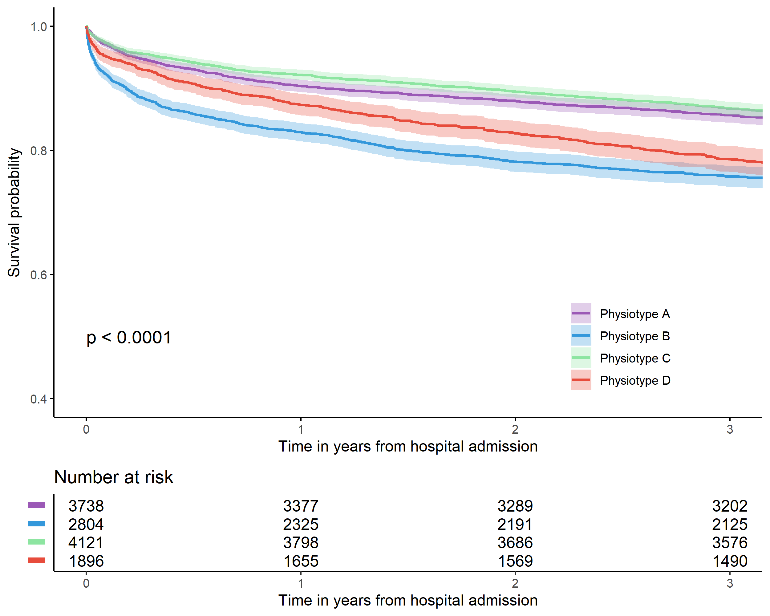


(C)


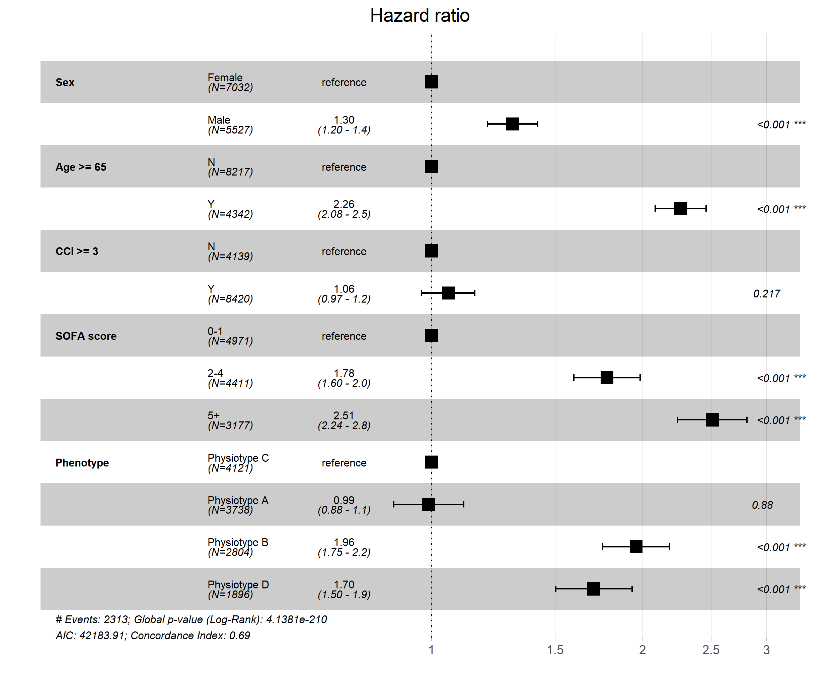


(D)


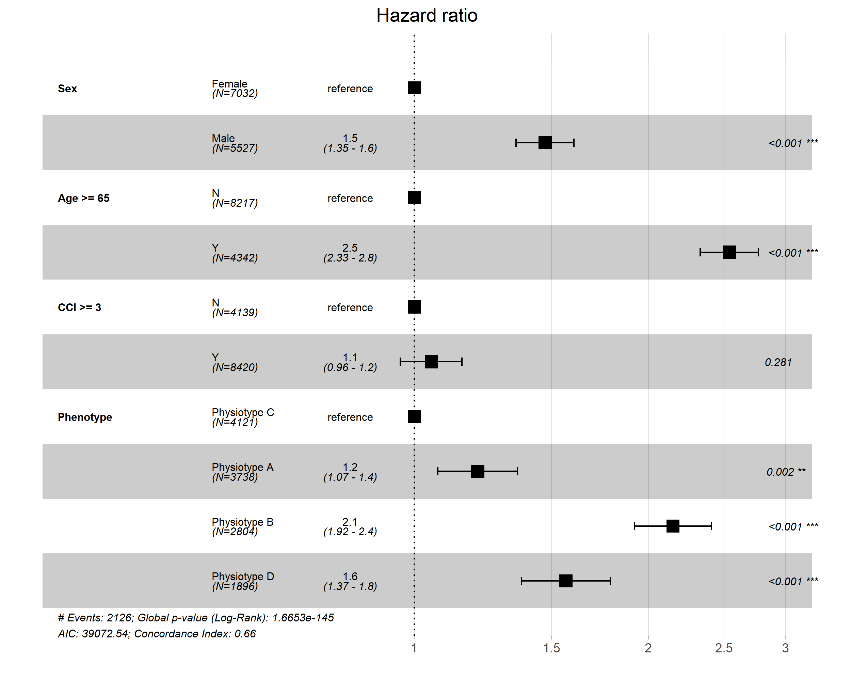


(C)

*(A) Physiotype survival curves adjusted using demographic information and comorbidities. (B) Adjusted Cox proportional hazards models using demographic information and comorbidities. (C) Physiotype survival curves adjusted using demographic information, comorbidities, and SOFA scores. (D) Adjusted Cox proportional hazards model using demographic information, comorbidities, and SOFA scores. Abbreviation: CCI: charlson comorbidity index; SOFA: sequential organ failure assessment.*
