# Supplementary material for: Physiologic signatures within six hours of hospitalization identify acute illness phenotypes
Source: PLOS Digit Health. 2022 Oct 13;1(10):e0000110. doi: 10.1371/journal.pdig.0000110 (PMC9802629; doi:10.1371/journal.pdig.0000110)
Supplement: S26 Fig — Diagnosis groups are shown in order of frequencies of all patients. For each phenotype, the larger percentage of patients with that diagnosis, the border the ribbon. Detailed diagnosis groups from left to right are: Nonspecific chest pain, Other and unspecific lower respiratory disease, Speticemia (except in labor), Abdominal pain, Complication of device; implant or graft, Acute cerebrovascular disease, Cardiac dysrhythmias, Osteoarthritis, and Other complications of pregnancy. (DOCX) [file pdig.0000110.s027.docx]

# S26 Fig. Chord diagrams showing the distribution of nine most common admission diagnosis groups by phenotype in testing cohort

(i) All physiotypes


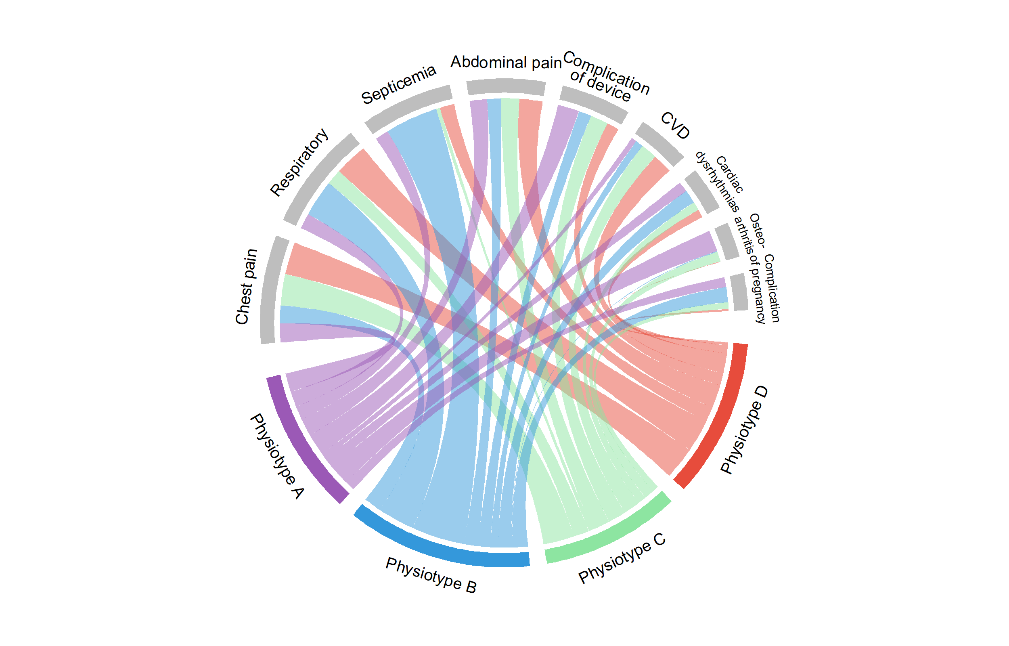


(iii) Physiotype B


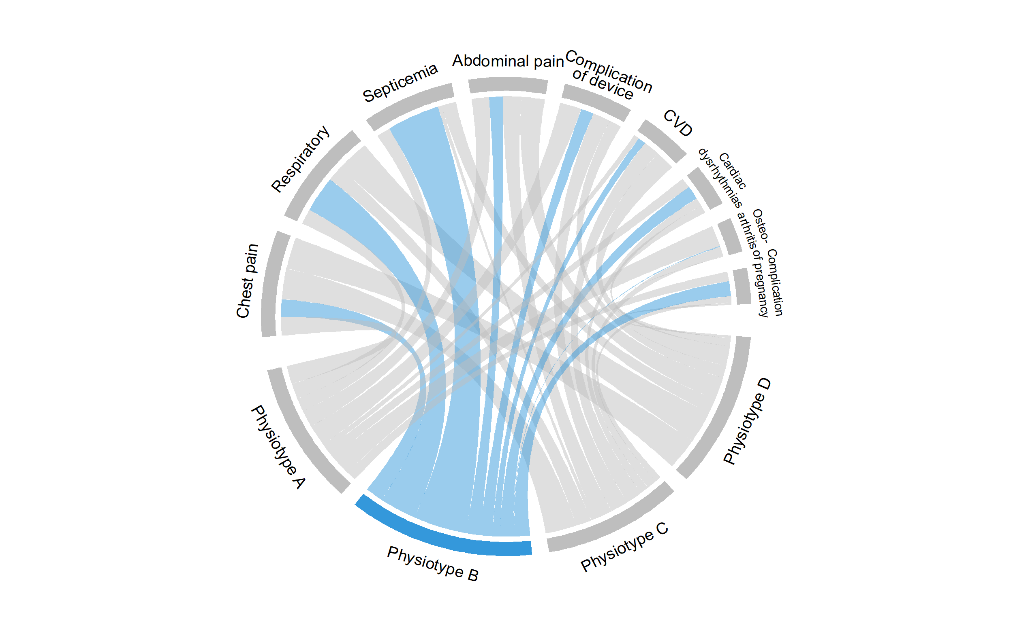


(ii) Physiotype A


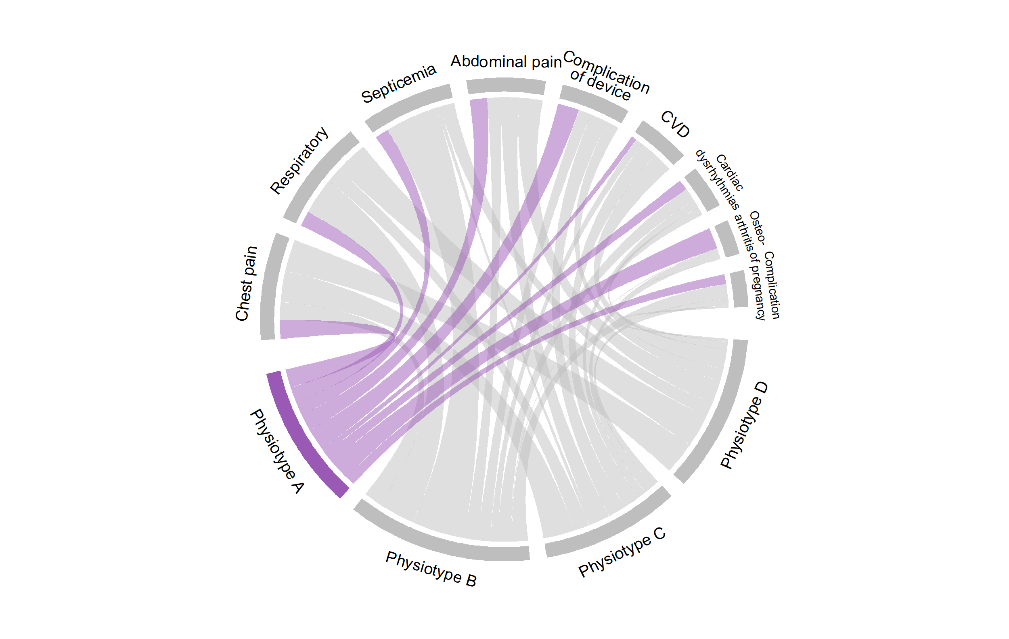


(v) Physiotype D


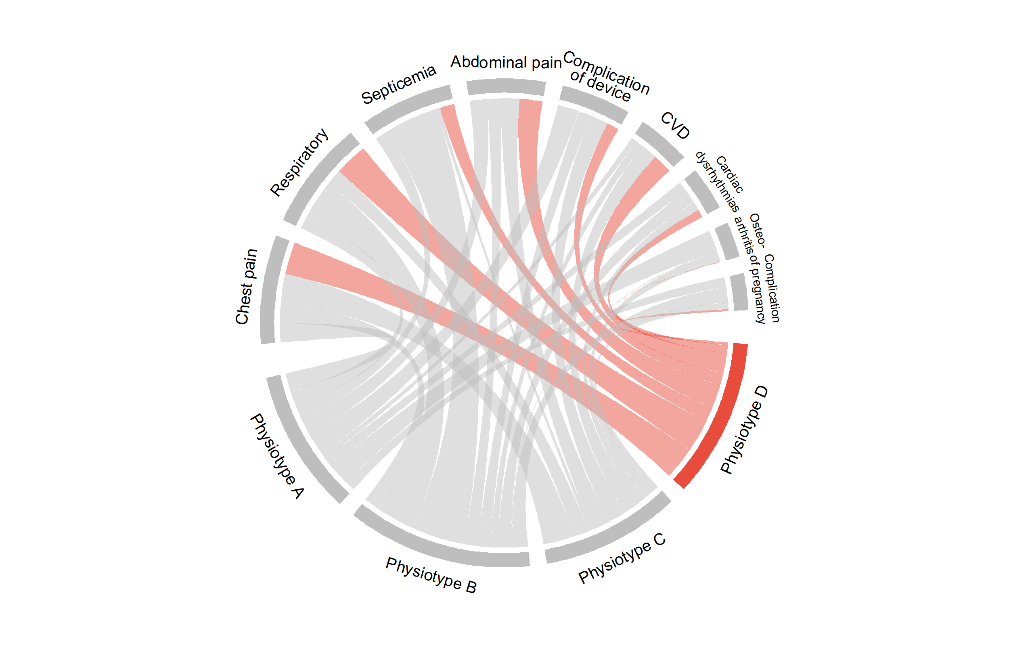


(iv) Physiotype C


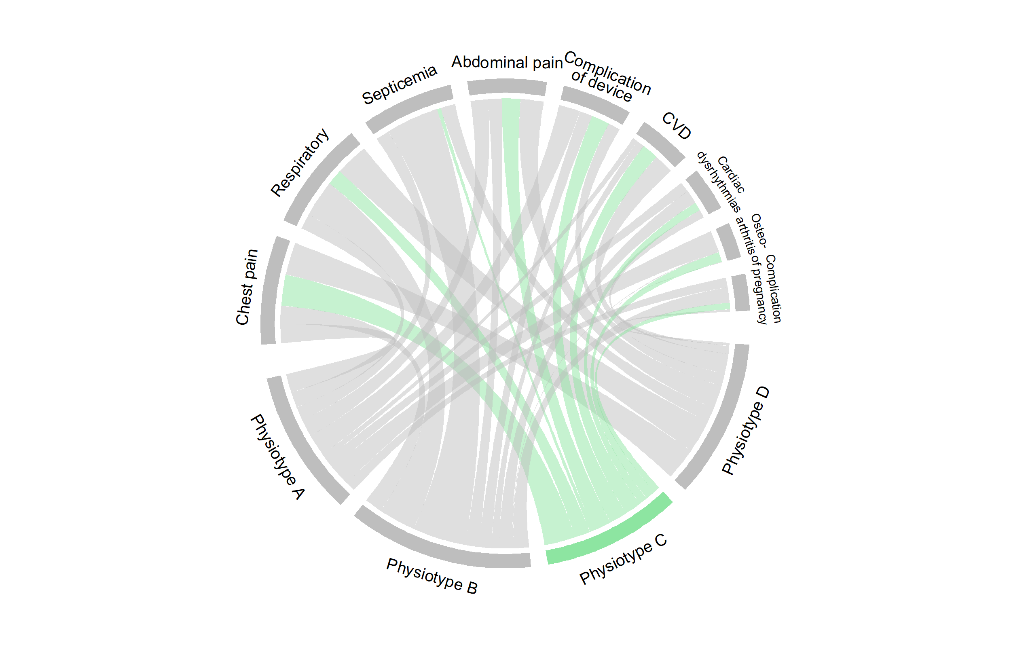


*Diagnosis groups are shown in order of frequencies of all patients. For each phenotype, the larger percentage of patients with that diagnosis, the border the ribbon. Detailed diagnosis groups from left to right are: Nonspecific chest pain, Other and unspecific lower respiratory disease, Speticemia (except in labor), Abdominal pain, Complication of device; implant or graft, Acute cerebrovascular disease, Cardiac dysrhythmias, Osteoarthritis, and Other complications of pregnancy.*
