# Supplementary material for: Physiologic signatures within six hours of hospitalization identify acute illness phenotypes
Source: PLOS Digit Health. 2022 Oct 13;1(10):e0000110. doi: 10.1371/journal.pdig.0000110 (PMC9802629; doi:10.1371/journal.pdig.0000110)
Supplement: S27 Fig — Interpretive example: Using gaussian mixture modeling to derive phenotypes, histograms of within phenotype probability demonstrated that members have high probability of being a phenotype member (>0.9). (DOCX) [file pdig.0000110.s028.docx]

# S27 Fig. Sensitivity analysis using gaussian mixture modeling clustering in training cohort (N=41,502), showing probabilities of phenotype assignment


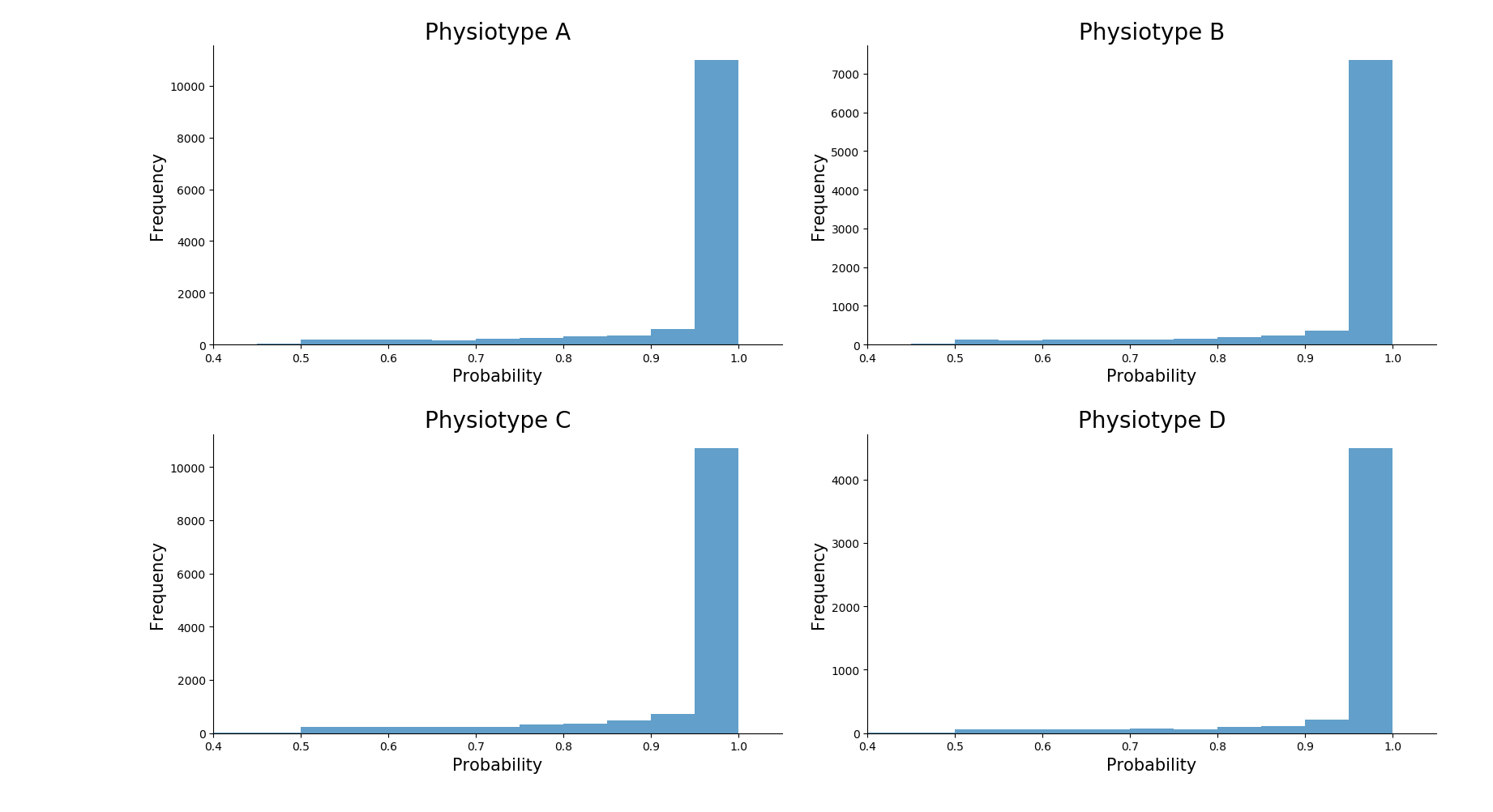


*Interpretive example*: Using gaussian mixture modeling to derive phenotypes, histograms of within phenotype probability demonstrated that members have high probability of being a phenotype member (>0.9).
