# Supplementary material for: Physiologic signatures within six hours of hospitalization identify acute illness phenotypes
Source: PLOS Digit Health. 2022 Oct 13;1(10):e0000110. doi: 10.1371/journal.pdig.0000110 (PMC9802629; doi:10.1371/journal.pdig.0000110)
Supplement: S29 Fig — Visualization of phenotypes using t-distributed stochastic neighbor embedding (t-SNE) technique in the training cohort with (A) physiotypes derived by consensus clustering shown in color, and (B) physiotypes derived by gaussian mixture modeling (GMM) shown in color. (DOCX) [file pdig.0000110.s030.docx]

# S29 Fig. t-SNE plot of phenotype assignments in training cohort


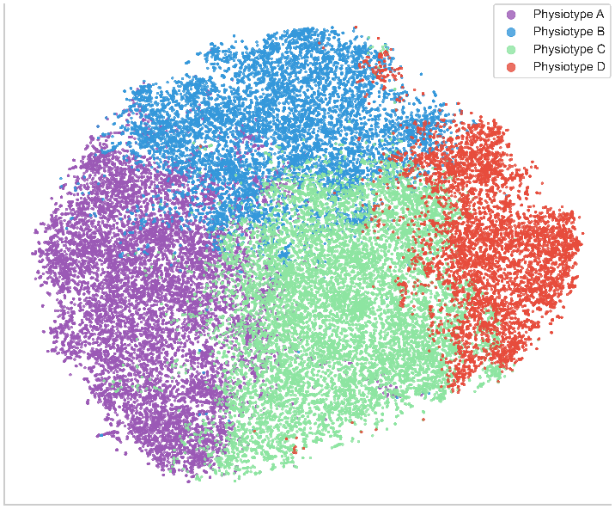

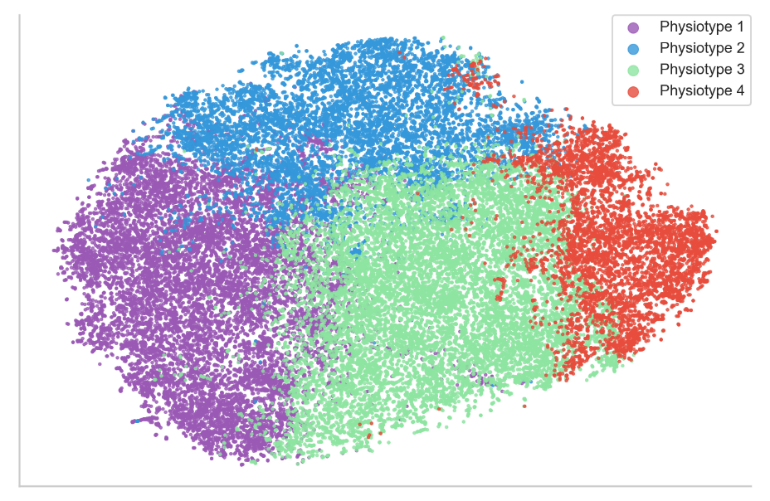


(A) Physiotypes derived by consensus clustering (B) Physiotypes derived by GMM

Visualization of phenotypes using t-distributed stochastic neighbor embedding (t-SNE) technique in the training cohort with (A) physiotypes derived by consensus clustering shown in color, and (B) physiotypes derived by gaussian mixture modeling (GMM) shown in color.
