# Supplementary material for: Physiologic signatures within six hours of hospitalization identify acute illness phenotypes
Source: PLOS Digit Health. 2022 Oct 13;1(10):e0000110. doi: 10.1371/journal.pdig.0000110 (PMC9802629; doi:10.1371/journal.pdig.0000110)
Supplement: S30 Fig — (A) Probabilities of assignment to cluster 1, and purple for those actually assigned to cluster 1, (B) Probabilities for patients assigned to cluster 2, and blue for those actually assigned to cluster 2, (C) Probabilities for patients assigned to cluster 3, and green for those actually assigned to cluster 3, and (D) probabilities for patients assigned to cluster 4, and orange for those actually assigned to cluster 4. Black lines correspond to median [IQR] of probability. Gray shading corresponds to region with a 45–55% (low or marginal) probability of assignment. Inset proportion is the % of 41,502 in the marginal region. (DOCX) [file pdig.0000110.s031.docx]

# S30 Fig. Probabilities of assignment for phenotype members and for those not assigned, using gaussian mixture modeling in the training cohort (N=41,502)


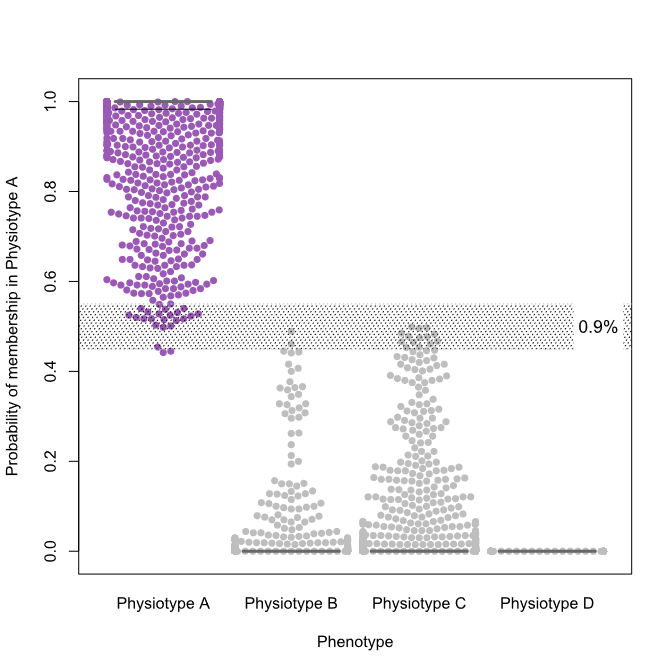

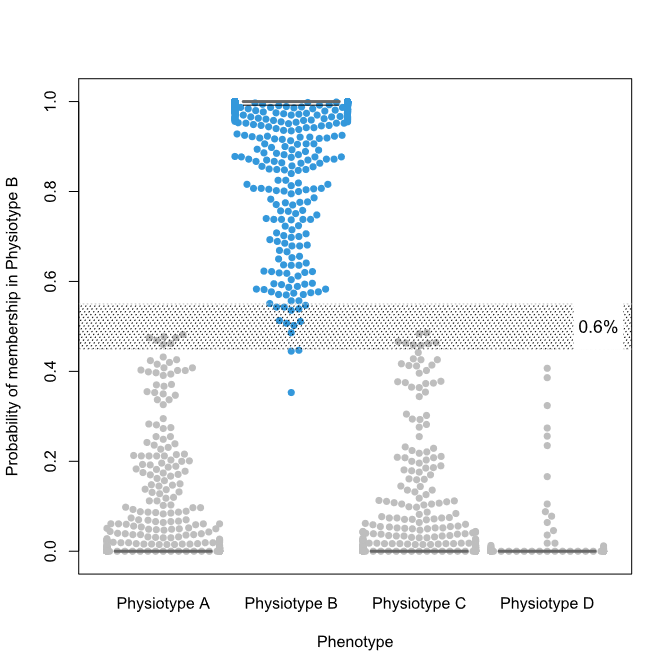


1. (B)


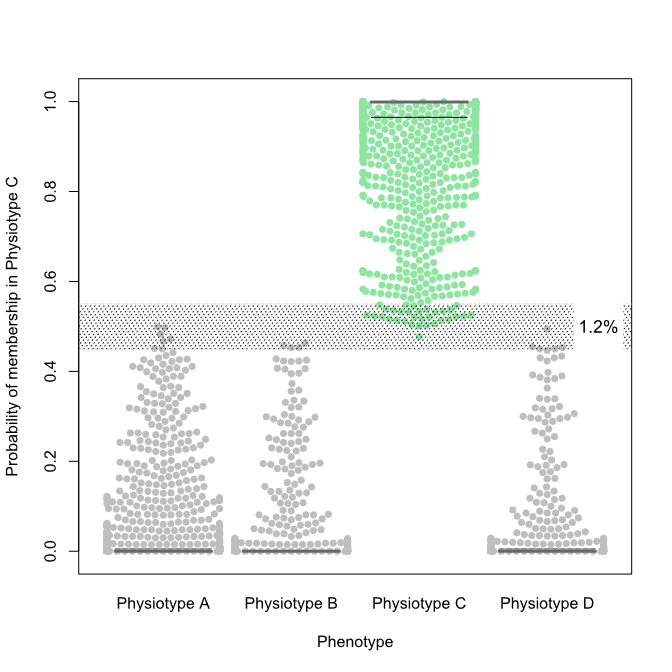

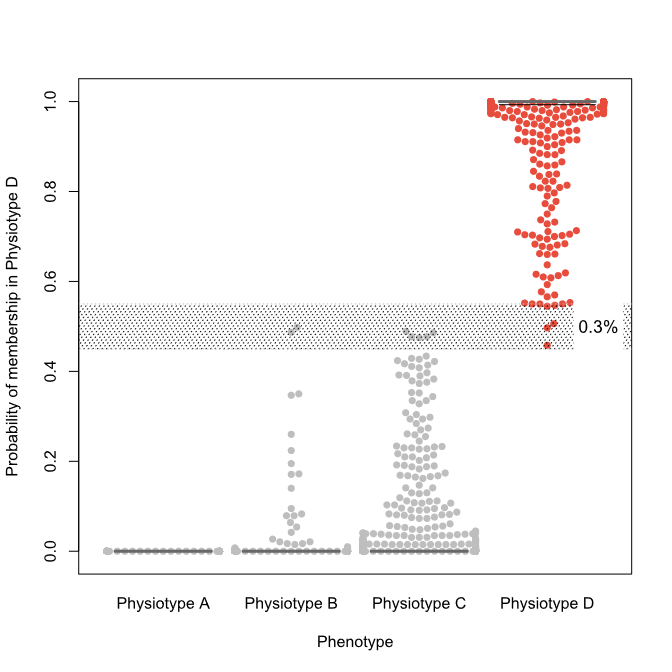


(C) (D)

(A) Probabilities of assignment to cluster 1, and *purple* for those actually assigned to cluster 1, (B) Probabilities for patients assigned to cluster 2, and *blue* for those actually assigned to cluster 2, (C) Probabilities for patients assigned to cluster 3, and *green* for those actually assigned to cluster 3, and (D) probabilities for patients assigned to cluster 4, and *orange* for those actually assigned to cluster 4. Black lines correspond to median [IQR] of probability. Gray shading corresponds to region with a 45-55% (low or marginal) probability of assignment. Inset proportion is the % of 41,502 in the marginal region.
