# Supplementary material for: Physiologic signatures within six hours of hospitalization identify acute illness phenotypes
Source: PLOS Digit Health. 2022 Oct 13;1(10):e0000110. doi: 10.1371/journal.pdig.0000110 (PMC9802629; doi:10.1371/journal.pdig.0000110)
Supplement: S1 Table — (DOCX) [file pdig.0000110.s032.docx]

# S1 Table. Processing of vital sign time series

| Variables | Unit | Non-outlier range [min, max]^a^ | Frequency (per hour) | Missing any measurement (N = 75,762), n (%) | Values used to impute variable completely missing^b^ | Normal distribution |
| --- | --- | --- | --- | --- | --- | --- |
| Systolic blood pressure | mmHg | (20, 300) | 2 | 0 (0) | 116 | Yes |
| Diastolic blood pressure | mmHg | (5, 225) | 2 | 0 (0) | 65 | Yes |
| Heart rate | beats per minute | (0, 300] | 2 | 9 (0) | 74.5 | Yes |
| Temperature | degree Celsius | (24, 45) | 1 | 10,713 (14) | 37.1 | No |
| Peripheral capillary oxygen saturation | % | (1, 100] | 2 | 3,744 (5) | 98.5 | No |
| Respiratory rate | breaths per minute | (0, 60] | 2 | 470 (1) | 12 | Yes |

^a^Derived from expert-defined ranges. Open brackets “)“ indicate value is not included and closed brackets “]” indicate value is included in the interval.

**^b^**Raw time series were resampled to an hourly frequency, taking the mean value when multiple measurements existed during the same one-hour window. Following resampling, gaps in the resulting time series were filled by first forward-propagating previous values and then back-propagating posterior values. For all remaining missing values, including instances in which a variable was missing entirely from an admission, median values of corresponding variables measured values in the training cohort were imputed.
