# Supplementary material for: Physiologic signatures within six hours of hospitalization identify acute illness phenotypes
Source: PLOS Digit Health. 2022 Oct 13;1(10):e0000110. doi: 10.1371/journal.pdig.0000110 (PMC9802629; doi:10.1371/journal.pdig.0000110)
Supplement: S2 Table — (DOCX) [file pdig.0000110.s033.docx]

# S2 Table. Used LOINCS, range of values, direction of abnormal values for lab variables

| Lab variables | LOINCS | LOINC Description | Plausible Range^a^ | Direction of abnormal value | Missingness in all cohort (N = 75,762), n (%) | Missingness in training cohort (N = 41,502), n (%) | Missingness in validation cohort (N = 17,415), n (%) | Missingness in testing cohort (N = 16,845), n (%) | Normal distribution |
| --- | --- | --- | --- | --- | --- | --- | --- | --- | --- |
| Basic metabolic Panel (BMP) | **89044-2/24321-2** | Basic metabolic and albumin panel - Serum or Plasma/Basic metabolic 2000 panel - Serum or Plasma |  |  |  |  |  |  |  |
| Glucose | 2339-0, 2340-8,  2345-7, 41651-1*,  41652-9*, 41653-7*,  74774-1*, | Glucose in serum or plasma/blood | 25 - 1400 | Maximum, Minimum | 9,090 (12) | 4,783 (12) | 2,129 (12) | 2,178 (13) | No |
| Creatinine | 2160-0, 38483-4 | Creatinine in blood | 0 - 30 | Maximum | 10,045 (13) | 5,276 (13) | 2,353 (14) | 2,416 (14) | No |
| Bilirubin | 1975-2 | Bilirubin total in serum or plasma | 0 - 50 | Maximum | 38,086 (50) | 20,319 (49) | 8,853 (51) | 8,914 (53) | No |
| Albumin | 1751-7, 2862-1,  61151-7 | Albumin in serum or plasma | 0.6 - 6.0 | Minimum | 37,735 (50) | 20,134 (49) | 8,779 (50) | 8,822 (52) | Yes |
| Anion Gap | 33037-3, 10366-1 | Anion gap in Serum or Plasma | 1 - 40 | Maximum | 14,876 (20) | 9,531 (23) | 2,645 (15) | 2,700 (16) | Yes |
| CBC Panel | 57021-8 | CBC W Auto Differential panel - Blood |  |  |  |  |  |  |  |
| White Blood Cell Count | 26464-8, 6690-2 | Leukocytes [#/volume] in Blood | 0.1 - 240 | Maximum,  Minimum | 7,118 (9) | 3,676 (9) | 1,665 (10) | 1,777 (11) | No |
| Hemoglobin | 718-7,  14775-1*, 30313-1*,  30352-9*, | Hemoglobin [Mass/volume] in Blood | 3 - 23 | Minimum | 6,108 (8) | 3,112 (7) | 1,443 (8) | 1,553 (9) | Yes |
| Platelets | 26515-7,  777-3, 49497-1* | Platelets [#/volume] in Blood | 2 - 1900 | Minimum | 7,143 (9) | 3,684 (9) | 1,671 (10) | 1,788 (11) | Yes |
| Bands % | 26508-2,  ,35332-6,  764-1* | Band form neutrophils/100 leukocytes in blood | 0.9 - 90 | Maximum | 72,187 (95) | 39,455 (95) | 16,625 (95) | 16,107 (96) | Yes |
| Lymphocytes % | 736-9, 737-7 | Lymphocytes/100 leukocytes in blood | 0 - 100 | Maximum,  Minimum | 22,653 (30) | 12,079 (29) | 5,275 (30) | 5,299 (31) | Yes |
| Gas Panel |  |  |  |  |  |  |  |  |  |
| Gas Panel-Arterial blood | 24336-0 |  |  |  |  |  |  |  |  |
| PH | 2744-1 | pH of arterial blood | 5 - 8 | Maximum,  Minimum | 64,519 (85) | 35,387 (85) | 14,894 (86) | 14,238 (85) | No |
| PO2 | 2703-7 | Oxygen [Partial pressure] in arterial blood | 0 - 800 | Minimum | 64,519 (85) | 35,386 (85) | 14,894 (86) | 14,239 (85) | No |
| Base deficit | 1922-4 | Base deficit in Arterial blood | 0 - 30 | Maximum | 69,565 (92) | 38,135 (92) | 15,986 (92) | 15,444 (92) | No |
| RDW | 788-0, 21000-5 | Erythrocyte distribution width [Ratio] | 2 - 40 | Maximum,  Minimum | 7,113 (9) | 3,672 (9) | 1,664 (10) | 1,777 (11) | Yes |
| Others |  |  |  |  |  |  |  |  |  |
| C-Reactive Protein (all sensitivity levels) | 30522-7, 1988-5 | C reactive protein in serum or plasma | 0 - 280 | Maximum | 65,389 (86) | 35,640 (86) | 15,159 (87) | 14,590 (87) | Yes |
| Lactate | 2518-9, 2524-7,  32693-4, 14118-4,  30242-2 | Lactate in blood | 0.3 - 28 | Maximum | 47,936 (63) | 26,055 (63) | 11,178 (64) | 10,703 (64) | No |
| ESR | 4537-7, 30341-2,  18184-2, 43402-7,  4538-5, 4539-3,  82477-1 | Erythrocyte sedimentation rate | 1 - 140 | Maximum | 69,214 (91) | 37,599 (91) | 16,033 (92) | 15,582 (93) | Yes |
| INR | 34714-6, 6301-6 | International normalized ratio | 0.8 - 18 | Maximum | 40,755 (54) | 21,145 (51) | 9,835 (56) | 9,775 (58) | No |
| Troponin (TnT, TnI) | 6598-7, 48425-3, 6597-9, 67151-1,10839-9, 42757-5,  49563-0 | Troponin T.cardiac in blood and Troponin I.cardiac in blood | 0 - 49 | Maximum | 49,379 (65) | 26,886 (65) | 11,553 (66) | 10,940 (65) | No |

^a^ Values out of the range values were removed.

Abbreviations: BUN: blood urea nitrogen; CO2: carbon dioxide; AST: aspartate transaminase; ALT: alanine transaminase; PO2: partial pressure of oxygen; RDW: red cell distribution width; ESR: erythrocyte sedimentation rate; INR: international normalized ratio.

*Included LOINCs that have same description from other panels.
