# Supplementary material for: Physiologic signatures within six hours of hospitalization identify acute illness phenotypes
Source: PLOS Digit Health. 2022 Oct 13;1(10):e0000110. doi: 10.1371/journal.pdig.0000110 (PMC9802629; doi:10.1371/journal.pdig.0000110)
Supplement: S3 Table — (DOCX) [file pdig.0000110.s034.docx]

# S3 Table. Clinical characteristics and biomarkers of the cohorts

| **Variables** | **Overall cohort** | **Training cohort** | **Validation Cohort** | **Testing Cohort** |
| --- | --- | --- | --- | --- |
| Number of Encounters (%) | 75,762 | 41,502 (55) | 17,415 (23) | 16,845 (22) |
| **Preadmission clinical characteristics** |  |  |  |  |
| Age, mean (SD) | 54 (19) | 54 (19) | 54 (19)^a^ | 55 (19)^a,b^ |
| Female sex, n (%) | 41,449 (55) | 22,745 (55) | 9,499 (55) | 9,205 (55) |
| Race, n (%) |  |  |  |  |
| White | 53,101 (70) | 29,076 (70) | 12,171 (70) | 11,854 (70) |
| African American | 17,432 (23) | 9,634 (23) | 3,953 (23) | 3,845 (23) |
| Primary Insurance, n (%) |  |  |  |  |
| Private | 17,641 (23) | 9,591 (23) | 4,115 (24) | 3,935 (23) |
| Medicare | 33,969 (45) | 18,499 (45) | 7,625 (44) | 7,845 (47)^a,b^ |
| Medicaid | 16,742 (22) | 9,231 (22) | 3,919 (23) | 3,592 (21)^a,b^ |
| Uninsured | 7,410 (10) | 4,181 (10) | 1,756 (10) | 1,473 (9)^a,b^ |
| Residency area characteristics |  |  |  |  |
| Total Proportion of African-American (%), mean (SD) | 18.8 (17.5) | 18.7 (17.5) | 18.9 (17.5) | 18.7 (17.4) |
| Population Proportion Below Poverty (%), mean (SD) | 22.6 (10.1) | 22.7 (10.1) | 22.7 (10.3) | 22.5 (10.2)^a^ |
| distance from Residency to Hospital (mile), median (IQR) | 18 (3, 34) | 18 (3, 34) | 18 (3, 34) | 18 (3, 34) |
| **Comorbidities** |  |  |  |  |
| Hypertension, n (%) | 38,985 (51) | 21,639 (52) | 8,878 (51)^a^ | 8,468 (50)^a^ |
| Cardiovascular disease, n (%)^c^ | 21,743 (29) | 12,058 (29) | 4,983 (29) | 4,702 (28)^a^ |
| Diabetes mellitus, n (%) | 18,127 (24) | 10,111 (24) | 4,071 (23)^a^ | 3,945 (23)^a^ |
| Chronic kidney disease, n (%) | 12,357 (16) | 6,518 (16) | 2,947 (17)^a^ | 2,892 (17)^a^ |
| **Admission characteristics of patients** |  |  |  |  |
| Emergent Admission, n (%) | 55,008 (73) | 30,177 (73) | 12,542 (72) | 12,289 (73) |
| Transfer from another hospital, n (%) | 13,569 (18) | 7,115 (17) | 3,595 (21)^a^ | 2,859 (17)^b^ |
| **Primary admission diagnostic groups** |  |  |  |  |
| Diseases of the circulatory system, n (%) | 13,670 (18) | 7,719 (19) | 2,968 (17)^a^ | 2,983 (18)^a^ |
| Respiratory and infectious diseases, n (%) | 6,016 (8) | 3,306 (8) | 1,185 (7)^a^ | 1,525 (9)^a,b^ |
| Complications of pregnancy and childbirth, n (%) | 5,760 (8) | 3,148 (8) | 1,366 (8) | 1,246 (7) |
| Diseases of the digestive/genitourinary systems, n (%) | 9,532 (13) | 5,184 (12) | 2,201 (13) | 2,147 (13) |
| Diseases of the musculoskeletal/connective tissue and skin, n (%) | 6,591 (9) | 3,651 (9) | 1,522 (9) | 1,418 (8) |
| Neoplasms, n (%) | 4,953 (7) | 2,743 (7) | 1,136 (7) | 1,074 (6) |
| **Clinical biomarkers and interventions within 24 hours of admission** |  |  |  |  |
| Surgery on admission day, n (%) | 15,996 (21) | 8,644 (21) | 3,801 (22)^a^ | 3,551 (21) |
| ICU/IMC admission within first 24 hours, n (%) | 17,163 (23) | 9,426 (23) | 3,899 (22) | 3,838 (23) |
| **Cardiovascular system** |  |  |  |  |
| Hypotension (MAP < 60 mmHg) at any time, n (%) | 26,400 (35) | 14,470 (35) | 6,014 (35) | 5,916 (35) |
| Duration, median (IQR), minutes | 57 (15, 165) | 57 (15, 168) | 53 (14, 157) | 60 (15, 167)^b^ |
| Vasopressors used, n (%) | 13,991 (18) | 7,531 (18) | 3,294 (19) | 3,166 (19) |
| Out of operating room | 2,596 (3) | 1,403 (3) | 625 (4) | 568 (3) |
| Hypertension (SBP > 160 mmHg) at any time, n (%) | 2,7267 (36) | 14,838 (36) | 6,222 (36) | 6,207 (37)^a^ |
| Troponin, tested, n (%) | 26,383 (35) | 14,616 (35) | 5,862 (34)^a^ | 5,905 (35)^b^ |
| Abnormal result among tested, n (%) | 5,842 (22) | 3,398 (23) | 1,239 (21)^a^ | 1,205 (20)^a^ |
| **Respiratory system** |  |  |  |  |
| Highest administered FiO2, median (IQR) | 0.21 (0.21, 0.40) | 0.21 (0.21, 0.40) | 0.21 (0.21, 0.40) | 0.21 (0.21, 0.40) |
| Room air only, n (%) | 43,887 (58) | 23,963 (58) | 10,242 (59)^a^ | 9,682 (57)^b^ |
| 0.22 - 0.40, n (%) | 26,997 (36) | 14,790 (36) | 6,125 (35) | 6,082 (36) |
| > 0.4, n (%) | 4,878 (6) | 2,749 (7) | 1,048 (6)^a^ | 1,081 (6) |
| PaO2/FiO2, tested with arterial blood gas, n (%) | 11,235 (15) | 6,113 (15) | 2,519 (14) | 2,603 (15)^b^ |
| <200 among tested, n (%) | 4,137 (37) | 2,265 (37) | 908 (36) | 964 (37) |
| Mechanical ventilation, n (%) | 3,924 (5) | 2,123 (5) | 883 (5) | 918 (5) |
| **Kidney and acid-base status** |  |  |  |  |
| Preadmission estimated glomerular filtration rate^d^ (mL/min per 1.73 m2), median (IQR) | 95 (77, 111) | 95 (78, 111) | 95 (76, 111) | 94 (76, 110)^a^ |
| Highest / reference creatinine^d^, mean (SD) | 1.24 (0.75) | 1.24 (0.66) | 1.24 (0.74)^a^ | 1.23 (0.95)^a^ |
| Renal replacement therapy, n (%) | 1,166 (1.5) | 641 (1.5) | 257 (1.5) | 268 (1.6) |
| Highest Anion Gap, median (IQR), mmol/L | 14 (12, 17) | 14 (12, 17) | 14 (13, 17)^a^ | 14 (12, 17)^a,b^ |
| Arterial Blood Gas tested, n (%) | 11,242 (15) | 6,115 (15) | 2,521 (14) | 2,606 (15)^b^ |
| pH < 7.3 among tested, n (%) | 2,580 (23) | 1,437 (23) | 557 (22) | 586 (22) |
| Highest Base deficit, mean (SD), mmol/L | 4.8 (4.7) | 4.8 (4.7) | 4.9 (4.6) | 4.6 (4.8)^a,b^ |
| Lactate, tested, n (%) | 27,826 (37) | 15,447 (37) | 6,237 (36)^a^ | 6,142 (36) |
| 2 - 4 mmol/L among tested, n (%) | 6,717 (24) | 3,739 (24) | 1,498 (24) | 1,480 (24) |
| > 4 mmol/L among tested, n (%) | 2,532 (9) | 1,374 (9) | 578 (9) | 580 (9) |
| **Inflammation** |  |  |  |  |
| Highest White blood cell count, median (IQR), x10^9/L | 9 (7, 13) | 9 (7, 13) | 9 (7, 13)^a^ | 9 (7, 12)^a^ |
| Highest Premature neutrophils (bands)), median (IQR), % | 10 (4, 20) | 10 (4, 20) | 9 (3, 19) | 9 (4, 18) |
| Lowest Lymphocytes, median (IQR), % | 16 (9, 24) | 16 (9, 24) | 16 (9, 24) | 16 (9, 24) |
| C-reactive protein, tested, n (%) | 10373 (14) | 5862 (14) | 2256 (13)^a^ | 2255 (13) |
| Highest C-reactive protein, median (IQR), mg/L | 18 (5, 81) | 18 (5, 77) | 17 (4, 80) | 28 (5, 93)^a,b^ |
| Erythrocyte sedimentation rate, tested, n (%) | 6548 (9) | 3903 (9) | 1382 (8)^a^ | 1263 (7)^a^ |
| Highest Erythrocyte sedimentation rate, median (IQR), mm/h | 40 (19, 74) | 40 (19, 73) | 42 (19, 77) | 41 (20, 73) |
| Highest Temperature, mean (SD), celsius | 37.7 (0.6) | 37.7 (0.6) | 37.7 (0.6) | 37.7 (0.6)^a,b^ |
| 38 - 39, n (%) | 15,774 (21) | 8,633 (21) | 3,563 (20) | 3,578 (21) |
| > 39, n (%) | 2,779 (4) | 1,548 (4) | 604 (3) | 627 (4) |
| Lowest Temperature, mean (SD), celsius | 36.7 (0.9) | 36.7 (1.0) | 36.7 (0.9)^a^ | 36.8 (0.9)^a,b^ |
| **Hematologic** |  |  |  |  |
| Lowest Hemoglobin, mean (SD), g/dL | 11.4 (2.3) | 11.5 (2.3) | 11.4 (2.3)^a^ | 11.2 (2.3)^a,b^ |
| Highest RDW, mean (SD), % | 15.4 (2.1) | 15.5 (2.1) | 15.2 (2.1)^a^ | 15.3 (2.1)^a,b^ |
| Lowest Platelets, median (IQR), x10^9/L | 208 (160, 266) | 210 (161, 269) | 204 (157, 260)^a^ | 207 (160, 266)^a,b^ |
| Platelets < 200, n (%) | 31,078 (41) | 16,707 (40) | 7,489 (43)^a^ | 6,882 (41)^a^ |
| < 100 | 4,779 (15) | 2,643 (16) | 1,128 (15) | 1,008 (15) |
| 100 - 200 | 26,299 (85) | 14,064 (84) | 6,361 (85) | 5,874 (85) |
| International normalized ratio, tested, n (%) | 35,007 (46) | 20,357 (49) | 7,580 (44)^a^ | 7,070 (42)^a,b^ |
| >= 2 | 3,291 (9) | 1,836 (9) | 757 (10)^a^ | 698 (10) |
| **Neurologic** |  |  |  |  |
| Glasgow Coma Scale score, n (%) |  |  |  |  |
| Moderate (9 - 12) | 3,125 (4) | 1,708 (4) | 687 (4) | 730 (4) |
| Severe (<= 8) | 2,662 (4) | 1,482 (4) | 587 (3) | 593 (4) |
| **Liver and metabolic** |  |  |  |  |
| Bilirubin, tested, n (%) | 37,676 (50) | 21,183 (51) | 8,562 (49)^a^ | 7931 (47)^a,b^ |
| >= 2 mg/dL, n (%) | 2,530 (7) | 1,427 (7) | 607 (7) | 496 (6) |
| Highest Glucose, median (IQR), mg/dL | 127 (104, 170) | 126 (104, 170) | 126 (104, 169) | 128 (104, 172)^a,b^ |
| Albumin, tested, n (%) | 38,027 (50) | 21,368 (51) | 8,636 (50)^a^ | 8,023 (48)^a,b^ |
| < 2.5 | 2,159 (6) | 1,243 (6) | 471 (5) | 445 (6) |
| 2.5 - 3.5 | 12,118 (32) | 6,904 (32) | 2,665 (31)^a^ | 2,549 (32) |

Abbreviation: ICU: intensive care unit; IMC: intermediate care unit; MAP: mean aterial pressure; RDW: red cell distribution width; SD: standard deviation; IQR: interquartile range.

All p-values were adjusted for multiple comparisons using Bonferroni method.

^a^ p < 0.05 compared to training cohort.

^b^ p < 0.05 compared to validation cohort.

^c^ Cardiovascular disease was considered if there was a history of congestive heart failure, coronary artery disease of peripheral vascular disease.

^d^ Reference glomerular filtration rate and reference creatinine were derived without use of race correction (see S1 Text for details).
