# Supplementary material for: Physiologic signatures within six hours of hospitalization identify acute illness phenotypes
Source: PLOS Digit Health. 2022 Oct 13;1(10):e0000110. doi: 10.1371/journal.pdig.0000110 (PMC9802629; doi:10.1371/journal.pdig.0000110)
Supplement: S4 Table — (DOCX) [file pdig.0000110.s035.docx]

# S4 Table. Illness severity, clinical outcomes, and resource use of the cohorts

| **Variables** | **Overall cohort** | **Training cohort** | **Validation Cohort** | **Testing Cohort** |
| --- | --- | --- | --- | --- |
| Number of Encounters (%) | 75,762 | 41,502 (55) | 17,415 (23) | 16,845 (22) |
| **Acuity scores within 24h of admission** |  |  |  |  |
| SOFA score > 6, n (%) | 6,463 (9) | 3,506 (8) | 1,503 (9) | 1,454 (9) |
| Patients in ICU/IMC, SOFA score <= 6, n (%) | 12,477 (16) | 6,882 (17) | 2,795 (16) | 2,800 (17) |
| Patients in ICU/IMC, SOFA score > 6, n (%) | 4,686 (6) | 2,544 (6) | 1,104 (6) | 1,038 (6) |
| Patients in ward, SOFA score <= 6, n (%) | 56,822 (75) | 31,114 (75) | 13,117 (75) | 12,591 (75) |
| Patients in ward, SOFA score > 6, n (%) | 1,777 (2) | 962 (2) | 399 (2) | 416 (2) |
| MEWS score > 4, n (%) | 5,033 (7) | 2,828 (7) | 1,115 (6) | 1,090 (6) |
| Patients in ICU/IMC, MEWS score <= 4, n (%) | 13,316 (18) | 7,235 (17) | 3,041 (17) | 3,040 (18) |
| Patients in ICU/IMC, MEWS score > 4, n (%) | 3,847 (5.1) | 2,191 (5.3) | 858 (4.9) | 798 (4.7)^a^ |
| Patients in ward, MEWS score <= 4, n (%) | 57,413 (76) | 31,439 (76) | 13,259 (76) | 12,715 (75) |
| Patients in ward, MEWS score > 4, n (%) | 1,186 (2) | 637 (2) | 257 (1) | 292 (2) |
| **Resource use during hospitalization** |  |  |  |  |
| Hospital days, median (IQR) | 4 (2, 7) | 4 (2, 7) | 4 (2, 7) | 4 (2, 7) |
| Surgery at any time, n (%) | 21,436 (28) | 11,634 (28) | 5,084 (29)^a^ | 4,718 (28)^b^ |
| Admitted to ICU/IMC^c^, n (%) | 20,380 (27) | 11,121 (27) | 4,643 (27) | 4,616 (27) |
| Days in ICU/IMC^d^, median (IQR) | 4 (2, 7) | 4 (2, 7) | 4 (2, 7) | 4 (2, 7)^a,b^ |
| Days in ICU/IMC greater than 48 hrs, n (%) | 15,201 (75) | 8,332 (75) | 3,468 (75) | 3,401 (74) |
| Mechanical Ventilation, n (%) | 5,970 (8) | 3,218 (8) | 1,403 (8) | 1,349 (8) |
| Mechanical Ventilation hours, median (IQR)^e^ | 32 (12, 108) | 35 (14, 113) | 31 (11, 105)^a^ | 28 (10, 92)^a^ |
| Mechanical Ventilation greater than 2 calendar days, n (%) | 2,993 (50) | 1,661 (52) | 699 (50) | 633 (47)^a^ |
| Renal replacement therapy, n (%) | 2,316 (3) | 1,262 (3) | 524 (3) | 530 (3) |
| **Complications** |  |  |  |  |
| Acute kidney injury overall, n (%) | 12,547 (17) | 6,905 (17) | 2,901 (17) | 2,741 (16) |
| Community-acquired AKI, n (%) | 7,007 (56) | 3,839 (56) | 1,603 (55) | 1,565 (57) |
| Hospital-acquired AKI, n (%) | 5,540 (44) | 3,066 (44) | 1,298 (45) | 1,176 (43) |
| Worst AKI staging, n (%) |  |  |  |  |
| Stage 1 | 8,036 (64) | 4,360 (63) | 1,878 (65) | 1,798 (66) |
| Stage 2 | 2,407 (19) | 1,362 (20) | 533 (18) | 512 (19) |
| Stage 3 | 1,496 (12) | 848 (12) | 348 (12) | 300 (11) |
| Stage 3 with RRT | 608 (5) | 335 (5) | 142 (5) | 131 (5) |
| Venous Thromboembolism, n (%) | 2,902 (4) | 1,257 (3) | 708 (4)^a^ | 937 (6)^a,b^ |
| Sepsis, n (%) | 7,322 (10) | 3,750 (9) | 1,659 (10) | 1,913 (11)^a,b^ |
| Hospital disposition, n (%) |  |  |  |  |
| Hospital mortality | 2,134 (2.8) | 1,141 (2.7) | 480 (2.8) | 513 (3.0) |
| Another hospital, LTAC, SNF, Hospice | 8,423 (11.1) | 4,475 (10.8) | 2,002 (11.5)^a^ | 1,946 (11.6)^a^ |
| Home or short-term rehabilitation | 65,205 (86.1) | 35,886 (86.5) | 14,933 (85.7) | 14,386 (85.4)^a^ |
| Thirty-day mortality, n (%) | 2,984 (4) | 1,633 (4) | 646 (4) | 705 (4) |
| Three-year mortality, n (%) | 14,634 (19) | 8,013 (19) | 3,297 (19) | 3,324 (20) |

Abbreviation: SOFA: sequential organ failure assessment; MEWS: modified early warning score; ICU: intensive care unit; IMC: intermediate care unit; SD: standard deviation; IQR: interquartile range.

All p-values were adjusted for multiple comparisons using Bonferroni method.

^a^ p < 0.05 compared to training cohort.

^b^ p < 0.05 compared to validation cohort.

^c^ At any time during hospitalization.

^d^ Values were calculated among patients admitted to ICU/IMC.

^e^ Values were calculated among patients requiring MV.
