# Supplementary material for: Physiologic signatures within six hours of hospitalization identify acute illness phenotypes
Source: PLOS Digit Health. 2022 Oct 13;1(10):e0000110. doi: 10.1371/journal.pdig.0000110 (PMC9802629; doi:10.1371/journal.pdig.0000110)
Supplement: S5 Table — (DOCX) [file pdig.0000110.s036.docx]

# S5 Table. Physiotype clinical characteristics and biomarkers in the training cohort

| **Variables** | **Total** | **Acute Illness Physiotypes** | | | |
| --- | --- | --- | --- | --- | --- |
|  |  | Physiotype A | Physiotype B | Physiotype C | Physiotype D |
| Number of Encounters (%) | 41,502 | 12,695 (31) | 9,710 (23) | 12,962 (31) | 6,135 (15) |
| **Preadmission clinical characteristics** |  |  |  |  |  |
| Age, mean (SD) | 54 (19) | 53 (18)^a,b,c^ | 50 (20)^a,b^ | 56 (18) | 56 (17) |
| Female sex, n (%) | 22,745 (55) | 7,291 (57)^a,b^ | 5,585 (58)^a,b^ | 6,641 (51) | 3,228 (53) |
| Race, n (%) |  |  |  |  |  |
| White | 29,076 (70) | 9,577 (75)^a,b,c^ | 6,723 (69)^a,b^ | 9,195 (71) | 3,581 (58)^a^ |
| African American | 9,634 (23) | 2,090 (16)^a,b,c^ | 2,342 (24)^b^ | 2,947 (23) | 2,255 (37)^a^ |
| Primary Insurance, n (%) |  |  |  |  |  |
| Private | 9,591 (23) | 3,158 (25)^a,b^ | 2,278 (23)^b^ | 2,991 (23) | 1,164 (19)^a^ |
| Medicare | 18,499 (45) | 5,604 (44)^a,b,c^ | 3,852 (40)^a,b^ | 6,124 (47) | 2,919 (48) |
| Medicaid | 9,231 (22) | 2,767 (22)^a,c^ | 2,588 (27)^a,b^ | 2,566 (20) | 1,310 (21) |
| Uninsured | 4,181 (10) | 1,166 (9)^b^ | 9,92 (10)^b^ | 1,281 (10) | 742 (12)^a^ |
| Residency area characteristics |  |  |  |  |  |
| Total Proportion of African-American (%), mean (SD) | 18.7 (17.5) | 17.3 (16.1)^a,b,c^ | 19.3 (17.8)^a,b^ | 18.5 (17.3) | 21.3 (19.4)^a^ |
| Population Proportion Below Poverty (%), mean (SD) | 22.7 (10.1) | 21.8 (10.0)^a,b,c^ | 23.2 (9.9)^a,b^ | 22.6 (10.0) | 24.0 (10.4)^a^ |
| distance from Residency to Hospital (mile), median (IQR) | 18 (3, 34) | 22 (3, 37)^a,b,c^ | 14 (3, 32)^a,b^ | 18 (3, 34) | 14 (3, 27)^a^ |
| **Comorbidities** |  |  |  |  |  |
| Hypertension, n (%) | 21,639 (52) | 6,498 (51)^b^ | 5,000 (51)^b^ | 6,723 (52) | 3,418 (56)^a^ |
| Cardiovascular disease, n (%)^d^ | 12,058 (29) | 3,477 (27)^a,b,c^ | 2,833 (29)^b^ | 3,783 (29) | 1,965 (32)^a^ |
| Diabetes mellitus, n (%) | 10,111 (24) | 2,934 (23)^b,c^ | 2,400 (25)^b^ | 3,125 (24) | 1,652 (27)^a^ |
| Chronic kidney disease, n (%) | 6,518 (16) | 1,757 (14)^a,b^ | 1,454 (15)^b^ | 2,056 (16) | 1,251 (20)^a^ |
| **Admission characteristics of patients** |  |  |  |  |  |
| Emergent Admission, n (%) | 30,177 (73) | 7,367 (58)^a,b,c^ | 8,106 (83)^a,b^ | 9,244 (71) | 5,460 (89)^a^ |
| Transfer from another hospital, n (%) | 7,115 (17) | 1,943 (15)^b,c^ | 1,957 (20)^a,b^ | 2,100 (16) | 1,115 (18)^a^ |
| **Primary admission diagnostic groups** |  |  |  |  |  |
| Diseases of the circulatory system, n (%) | 7,719 (19) | 2,142 (17)^a,b,c^ | 1,503 (15)^a,b^ | 2,533 (20) | 1,541 (25)^a^ |
| Respiratory and infectious diseases, n (%) | 3,306 (8) | 571 (4)^a,b,c^ | 1,403 (14)^a,b^ | 692 (5) | 640 (10)^a^ |
| Complications of pregnancy and childbirth, n (%) | 3,148 (8) | 857 (7)^b,c^ | 1,100 (11)^a,b^ | 862 (7) | 329 (5)^a^ |
| Diseases of the digestive/genitourinary systems, n (%) | 5,184 (12) | 1,857 (15)^a,b,c^ | 1,028 (11)^a^ | 1,661 (13) | 638 (10)^a^ |
| Diseases of the musculoskeletal/connective tissue and skin, n (%) | 3,651 (9) | 1,489 (12)^a,b,c^ | 479 (5)^a,b^ | 1,216 (9) | 467 (8)^a^ |
| Neoplasms, n (%) | 2,743 (7) | 1,244 (10)^a,b,c^ | 377 (4)^a,b^ | 950 (7) | 172 (3)^a^ |
| **Clinical biomarkers and interventions within 24 hours of admission** |  |  |  |  |  |
| Surgery on admission day, n (%) | 8,644 (21) | 4,441 (35)^a,b,c^ | 796 (8)^a^ | 2,933 (23) | 474 (8)^a^ |
| ICU/IMC admission within first 24 hours, n (%) | 9,426 (23) | 2,893 (23)^a,c^ | 3,022 (31)^a,b^ | 2,151 (17) | 1,360 (22)^a^ |
| **Cardiovascular system** |  |  |  |  |  |
| Hypotension (MAP < 60 mmHg) at any time, n (%) | 14,470 (35) | 7,420 (58)^a,b,c^ | 3,393 (35)^a,b^ | 3,051 (24) | 606 (10)^a^ |
| Duration, median (IQR), minutes | 57 (15, 168) | 60 (18, 197)^a,b,c^ | 75 (30, 212)^a,b^ | 18 (6, 62) | 24 (8, 68) |
| Vasopressors used, n (%) | 7,531 (18) | 4,079 (32)^a,b,c^ | 995 (10)^a,b^ | 2,113 (16) | 344 (6)^a^ |
| Out of operating room | 1,403 (3) | 646 (5)^a,b^ | 494 (5)^a,b^ | 198 (2) | 65 (1) |
| Hypertension (SBP > 160 mmHg) at any time, n (%) | 14,838 (36) | 2,742 (22)^a,b,c^ | 1,611 (17)^a,b^ | 5,629 (43) | 4,856 (79)^a^ |
| Troponin, tested, n (%) | 14,616 (35) | 3,223 (25)^a,b,c^ | 4,090 (42)^a,b^ | 4,214 (33) | 3,089 (50)^a^ |
| Abnormal result among tested, n (%) | 3,398 (23) | 791 (25)^a^ | 987 (24)^a^ | 816 (19) | 804 (26)^a^ |
| **Respiratory system** |  |  |  |  |  |
| Highest administered FiO2, median (IQR) | 0.21 (0.21, 0.40) | 0.28 (0.21, 0.40)^a,b,c^ | 0.21 (0.21, 0.33)^a,b^ | 0.21 (0.21, 0.40) | 0.21 (0.21, 0.29)^a^ |
| Room air only, n (%) | 23,963 (58) | 6,273 (49)^a,b,c^ | 5,580 (57)^a,b^ | 8,040 (62) | 4,070 (66)^a^ |
| 0.22 - 0.40, n (%) | 14,790 (36) | 5,419 (43)^a,b,c^ | 3,285 (34)^b^ | 4,320 (33) | 1,766 (29)^a^ |
| > 0.4, n (%) | 2,749 (7) | 1,003 (8)^a,b^ | 845 (9)^a,b^ | 602 (5) | 299 (5) |
| PaO2/FiO2, tested with arterial blood gas, n (%) | 6,113 (15) | 2,015 (16)^a,b,c^ | 1,965 (20)^a,b^ | 1,345 (10) | 788 (13)^a^ |
| <200 among tested, n (%) | 2,265 (37) | 747 (37)^a,c^ | 837 (43)^a,b^ | 427 (32) | 254 (32) |
| Mechanical ventilation, n (%) | 2,123 (5) | 808 (6)^a,b^ | 656 (7)^a,b^ | 449 (3) | 210 (3) |
| **Kidney and acid-base status** |  |  |  |  |  |
| Preadmission estimated glomerular filtration rate^e^ (mL/min per 1.73 m2), median (IQR) | 95 (78, 111) | 96 (80, 112)^a,b,c^ | 100 (83, 117)^a,b^ | 93 (77, 107) | 90 (59, 105)^a^ |
| Highest / reference creatinine^e^ ratio, mean (SD) | 1.24(0.66) | 1.25 (0.71)^a,b,c^ | 1.31 (0.73)^a,b^ | 1.18 (0.54) | 1.24 (0.67)^a^ |
| Renal replacement therapy, n (%) | 641 (2) | 170 (1)^b^ | 119 (1)^b^ | 128 (1) | 224 (4)^a^ |
| Highest Anion Gap, median (IQR), mmol/L | 14 (12, 17) | 13 (11, 16)^b,c^ | 15 (12, 18)^a,b^ | 14 (11, 16) | 15 (12, 17)^a^ |
| Arterial Blood Gas tested, n (%) | 6,115 (15) | 2,016 (16)^a,b,c^ | 1,966 (20)^a,b^ | 1,345 (10) | 788 (13)^a^ |
| pH < 7.3 among tested, n (%) | 1,437 (23) | 532 (26)^a,b^ | 558 (28)^a,b^ | 216 (16) | 131 (17) |
| Highest Base deficit, mean (SD), mmol/L | 4.8 (4.7) | 4.4 (4.2)^a,c^ | 6.4 (5.8)^a,b^ | 3.6 (3.2) | 4.3 (3.7)^a^ |
| Lactate, tested, n (%) | 15,447 (37) | 4,360 (34)^a,b,c^ | 4,660 (48)^a,b^ | 4,006 (31) | 2,421 (39)^a^ |
| 2 - 4 mmol/L among tested, n (%) | 3,739 (24) | 1,012 (23)^c^ | 1,305 (28)^a,b^ | 854 (21) | 568 (23) |
| > 4 mmol/L among tested, n (%) | 1,374 (9) | 379 (9)^a,c^ | 607 (13)^a,b^ | 204 (5) | 184 (8)^a^ |
| **Inflammation** |  |  |  |  |  |
| Highest White blood cell count, median (IQR), x10^9/L | 9 (7, 13) | 9 (7, 13)^a,b,c^ | 10 (8, 14)^a,b^ | 9 (7, 12) | 9 (7, 12) |
| Highest Premature neutrophils (bands)), median (IQR), % | 10 (4, 20) | 10 (4, 17)^a,c^ | 12 (5, 24)^a,b^ | 5 (2, 14) | 8 (3, 15) |
| Lowest Lymphocytes, median (IQR), % | 16 (9, 24) | 16 (9, 26)^a,b,c^ | 12 (6, 20)^a,b^ | 18 (11, 26) | 17 (10, 24)^a^ |
| C-reactive protein, tested, n (%) | 5,862 (14) | 1,479 (12)^a,b,c^ | 1,694 (17)^a,b^ | 1,730 (13) | 959 (16)^a^ |
| Highest C-reactive protein, median (IQR), mg/L | 18 (5, 77) | 18 (5, 71)^a,b,c^ | 53 (11, 122)^a,b^ | 11 (3, 54) | 12 (4, 52) |
| Erythrocyte sedimentation rate, tested, n (%) | 3,903 (9) | 962 (8)^a,b,c^ | 1,021 (11) | 1,234 (10) | 686 (11)^a^ |
| Highest Erythrocyte sedimentation rate, median (IQR), mm/h | 40 (19, 73) | 37 (18, 66)^c^ | 51 (23, 88)^a,b^ | 34 (17, 65) | 40 (20, 72)^a^ |
| Highest Temperature, mean (SD), celsius | 37.7 (0.6) | 37.7 (0.6)^a,b,c^ | 37.9 (0.8)^a,b^ | 37.6 (0.5) | 37.7 (0.6)^a^ |
| 38 - 39, n (%) | 8,633 (21) | 2,869 (23)^a,b,c^ | 2,349 (24)^a,b^ | 2,259 (17) | 1,156 (19) |
| > 39, n (%) | 1,548 (4) | 349 (3)^a,c^ | 826 (9)^a,b^ | 211 (2) | 162 (3)^a^ |
| Lowest Temperature, mean (SD), celsius | 36.7 (1.0) | 36.5 (1.4)^a,b,c^ | 36.7 (0.8)^a^ | 36.7 (0.8) | 36.8 (0.7)^a^ |
| **Hematologic** |  |  |  |  |  |
| Lowest Hemoglobin, mean (SD), g/dL | 11.5(2.3) | 11.1 (2.3)^a,b,c^ | 11.2 (2.4)^a,b^ | 12.0 (2.2) | 12.0 (2.3) |
| Highest RDW, mean (SD), % | 15.5 (2.1) | 15.5 (2.2)^a,b,c^ | 15.9 (2.3)^a,b^ | 15.2 (1.9) | 15.5 (2.0)^a^ |
| Lowest Platelets, median (IQR), x10^9/L | 210 (161, 269) | 200 (152, 258)^a,b,c^ | 218 (161, 285)^a^ | 211 (166, 265) | 219 (169, 274)^a^ |
| Platelets < 200, n (%) | 16,707 (40) | 5,535 (44)^a,b,c^ | 3,874 (40)^a,b^ | 4,971 (38) | 2,327 (38)^a^ |
| < 100 | 2,643 (16) | 976 (18)^a,b,c^ | 785 (20)^a,b^ | 628 (13) | 254 (11) |
| 100 - 200 | 14,064 (84) | 4,559 (82)^a,b,c^ | 3,089 (80)^a,b^ | 4,343 (87) | 2,073 (89) |
| International normalized ratio, tested, n (%) | 20,357 (49) | 5,607 (44)^a,b,c^ | 5,193 (53)^a^ | 6,150 (47) | 3,407 (56)^a^ |
| >= 2 | 1,836 (9) | 586 (10)^a,b^ | 583 (11)^a,b^ | 465 (8) | 202 (6)^a^ |
| **Neurologic** |  |  |  |  |  |
| Glasgow Coma Scale score, n (%) |  |  |  |  |  |
| Moderate (9 - 12) | 1,708 (4) | 631 (5)^a,b^ | 479 (5)^a,b^ | 401 (3) | 197 (3) |
| Severe (<= 8) | 1,482 (4) | 477 (4)^a,c^ | 479 (5)^a,b^ | 336 (3) | 190 (3) |
| **Liver and metabolic** |  |  |  |  |  |
| Bilirubin, tested, n (%) | 21,183 (51) | 5,431 (43)^a,b,c^ | 5,902 (61)^a^ | 6,110 (47) | 3,740 (61)^a^ |
| >= 2 mg/dL, n (%) | 1,427 (7) | 527 (10)^a,b,c^ | 481 (8)^a,b^ | 306 (5) | 113 (3)^a^ |
| Highest Glucose, median (IQR), mg/dL | 126 (104, 170) | 125 (102, 165)^b,c^ | 129 (105, 175)^a,b^ | 124 (102, 167) | 132 (106, 186)^a^ |
| Albumin, tested, n (%) | 21,368 (51) | 5,508 (43)^a,b,c^ | 5,929 (61)^a^ | 6,172 (48) | 3,759 (61)^a^ |
| < 2.5 | 1,243 (6) | 403 (7)^a,b,c^ | 555 (9)^a,b^ | 180 (3) | 105 (3) |
| 2.5 - 3.5 | 6,904 (32) | 1,912 (35)^a,b,c^ | 2,292 (39)^a,b^ | 1,621 (26) | 1,079 (29) |

Abbreviation: ICU: intensive care unit; IMC: intermediate care unit; MAP: mean aterial pressure; RDW: red cell distribution width; SD: standard deviation; IQR: interquartile range.

All p-values were adjusted for multiple comparisons using Bonferroni method.

^a^ p < 0.05 compared to Physiotype C .

^b^ p < 0.05 compared to Physiotype D.

^c^ p < 0.05 compared to Physiotype B.

^d^ Cardiovascular disease was considered if there was a history of congestive heart failure, coronary artery disease of peripheral vascular disease.

^e^ Reference glomerular filtration rate and reference creatinine were derived without use of race correction (see S1 Text for details).
