# Supplementary material for: Physiologic signatures within six hours of hospitalization identify acute illness phenotypes
Source: PLOS Digit Health. 2022 Oct 13;1(10):e0000110. doi: 10.1371/journal.pdig.0000110 (PMC9802629; doi:10.1371/journal.pdig.0000110)
Supplement: S6 Table — (DOCX) [file pdig.0000110.s037.docx]

# S6 Table. Physiotype illness severity, clinical outcomes, and resource use in the training cohort

| **Variables** | **Total** | **Acute Illness Physiotypes** | | | |
| --- | --- | --- | --- | --- | --- |
|  |  | Physiotype A | Physiotype B | Physiotype C | Physiotype D |
| Number of Encounters (%) | 41,502 | 12,695 (31) | 9,710 (23) | 12,962 (31) | 6,135 (15) |
| **Acuity scores within 24h of admission** |  |  |  |  |  |
| SOFA score > 6, n (%) | 3,506 (8) | 1,494 (12)^a,b,c^ | 974 (10)^a,b^ | 720 (6) | 318 (5) |
| Patients in ICU/IMC, SOFA score <= 6, n (%) | 6,882 (17) | 1,868 (15)^a,b,c^ | 2,195 (23)^a,b^ | 1,693 (13) | 1,126 (18)^a^ |
| Patients in ICU/IMC, SOFA score > 6, n (%) | 2,544 (6) | 1,025 (8)^a,b^ | 827 (9)^a,b^ | 458 (4) | 234 (4) |
| Patients in ward, SOFA score <= 6, n (%) | 31,114 (75) | 9,333 (74)^a,b,c^ | 6,541 (67)^a,b^ | 10,549 (81) | 4,691 (76)^a^ |
| Patients in ward, SOFA score > 6, n (%) | 962 (2) | 469 (4)^a,b,c^ | 147 (2)^a^ | 262 (2) | 84 (1)^a^ |
| MEWS score > 4, n (%) | 2,828 (7) | 472 (4)^a,b,c^ | 1,549 (16)^a,b^ | 264 (2) | 543 (9)^a^ |
| Patients in ICU/IMC, MEWS score <= 4, n (%) | 7,235 (17) | 2,507 (20)^a,b^ | 1,785 (18)^a,b^ | 1,941 (15) | 1,002 (16) |
| Patients in ICU/IMC, MEWS score > 4, n (%) | 2,191 (5) | 386 (3)^a,b,c^ | 1,237 (13)^a,b^ | 210 (2) | 358 (6)^a^ |
| Patients in ward, MEWS score <= 4, n (%) | 31,439 (76) | 9,716 (77)^a,c^ | 6,376 (66)^a,b^ | 10,757 (83) | 4,590 (75)^a^ |
| Patients in ward, MEWS score > 4, n (%) | 637 (2) | 86 (1)^a,b,c^ | 312 (3)^a^ | 54 (0) | 185 (3)^a^ |
| **Resource use during hospitalization** |  |  |  |  |  |
| Hospital days, median (IQR) | 4 (2, 7) | 4 (2, 6)^a,c^ | 4 (3, 8)^a,b^ | 3 (2, 6) | 4 (2, 7)^a^ |
| Surgery at any time, n (%) | 11,634 (28) | 5,225 (41)^a,b,c^ | 1,502 (15)^a^ | 3,957 (31) | 950 (15)^a^ |
| Admitted to ICU/IMC^d^, n (%) | 11,121 (27) | 3,330 (26)^a,c^ | 3,504 (36)^a,b^ | 2,640 (20) | 1,647 (27)^a^ |
| Days in ICU/IMC^e^, median (IQR) | 4 (2, 7) | 4 (3, 7)^a,b^ | 4 (3, 8)^a,b^ | 4 (2, 7) | 4 (2, 6) |
| Days in ICU/IMC greater than 48 hrs, n (%) | 8,332 (75) | 2,517 (76)^a^ | 2,722 (78)^a,b^ | 1,872 (71) | 1,221 (74) |
| Mechanical Ventilation, n (%) | 3,218 (8) | 1,120 (9)^a,b,c^ | 1,036 (11)^a,b^ | 736 (6) | 326 (5) |
| Mechanical Ventilation hours, median (IQR)^f^ | 35 (14, 113) | 24 (11, 81)^b,c^ | 46 (17, 142)^a^ | 26 (12, 105) | 54 (21, 145)^a^ |
| Mechanical Ventilation greater than 2 calendar days, n (%) | 1,661 (52) | 492 (44)^b,c^ | 613 (59)^a^ | 349 (47) | 207 (63)^a^ |
| Renal replacement therapy, n (%) | 1,262 (3) | 335 (3)^a,b^ | 299 (3)^a,b^ | 265 (2) | 363 (6)^a^ |
| **Complications** |  |  |  |  |  |
| Acute kidney injury overall, n (%) | 6,905 (17) | 1,971 (16)^a,b,c^ | 2,119 (22)^a,b^ | 1,682 (13) | 1,133 (18)^a^ |
| Community-acquired AKI, n (%) | 3,839 (56) | 1,234 (63)^a,b,c^ | 1,221 (58)^a,b^ | 873 (52) | 511 (45)^a^ |
| Hospital-acquired AKI, n (%) | 3,066 (44) | 737 (37)^a,b,c^ | 898 (42)^a,b^ | 809 (48) | 622 (55)^a^ |
| Worst AKI staging, n (%) |  |  |  |  |  |
| Stage 1 | 4,360 (63) | 1,194 (61)^a,b^ | 1,241 (59)^a,b^ | 1,174 (70) | 751 (66) |
| Stage 2 | 1,362 (20) | 404 (21)^a^ | 484 (23)^a,b^ | 280 (17) | 194 (17) |
| Stage 3 | 848 (12) | 269 (14)^a^ | 276 (13)^a^ | 171 (10) | 132 (12) |
| Stage 3 with RRT | 335 (5) | 104 (5)^a^ | 118 (6)^a^ | 57 (3) | 56 (5) |
| Venous Thromboembolism, n (%) | 1,257 (3) | 341 (3)^c^ | 393 (4)^a,b^ | 350 (3) | 173 (3) |
| Sepsis, n (%) | 3,750 (9) | 902 (7)^a,c^ | 1,933 (20)^a,b^ | 500 (4) | 415 (7)^a^ |
| Hospital disposition, n (%) |  |  |  |  |  |
| Hospital mortality | 1,141 (3) | 291 (2)^a,c^ | 502 (5)^a,b^ | 227 (2) | 121 (2) |
| Another hospital, LTAC, SNF, Hospice | 4,475 (11) | 1,286 (10)^b,c^ | 1,231 (13)^a^ | 1,233 (10) | 725 (12)^a^ |
| Home or short-term rehabilitation | 35,886 (87) | 11,118 (88)^a,c^ | 7,977 (82)^a,b^ | 11,502 (89) | 5,289 (86)^a^ |
| Thirty-day mortality, n (%) | 1,633 (4) | 429 (3)^a,c^ | 684 (7)^a,b^ | 332 (3) | 188 (3) |
| Three-year mortality, n (%) | 8,013 (19) | 2,205 (17)^b,c^ | 2,466 (25)^a,b^ | 2,109 (16) | 1,233 (20)^a^ |

Abbreviation: SOFA: sequential organ failure assessment; MEWS: modified early warning score; ICU: intensive care unit; IMC: intermediate care unit; IQR: interquartile range.

All p-values were adjusted for multiple comparisons using Bonferroni method.

^a^ p < 0.05 compared to Physiotype C .

^b^ p < 0.05 compared to Physiotype D.

^c^ p < 0.05 compared to Physiotype B.

^d^ At any time during hospitalization.

^e^ Values were calculated among patients admitted to ICU/IMC.

^f^ Values were calculated among patients requiring MV.
