# Supplementary material for: Physiologic signatures within six hours of hospitalization identify acute illness phenotypes
Source: PLOS Digit Health. 2022 Oct 13;1(10):e0000110. doi: 10.1371/journal.pdig.0000110 (PMC9802629; doi:10.1371/journal.pdig.0000110)
Supplement: S7 Table — (DOCX) [file pdig.0000110.s038.docx]

# S7 Table. Physiotype clinical characteristics and biomarkers in the validation cohort

| **Variables** | **Total** | **Acute Illness Physiotypes** | | | |
| --- | --- | --- | --- | --- | --- |
|  |  | Physiotype A | Physiotype B | Physiotype C | Physiotype D |
| Number of Encounters (%) | 17,415 | 5,225 (30) | 3,923 (23) | 5,450 (31) | 2,817 (16) |
| **Preadmission clinical characteristics** |  |  |  |  |  |
| Age, mean (SD) | 54 (19) | 52 (19)^a,b,c^ | 50 (20) ^a,b^ | 56 (18) | 57 (17) |
| Female sex, n (%) | 9,499 (55) | 3,076 (59)^a,b^ | 2,251 (57)^a,b^ | 2,744 (50) | 1,428 (51) |
| Race, n (%) |  |  |  |  |  |
| White | 12,171 (70) | 3,901 (75)^a,b,c^ | 2,753 (70)^b^ | 3,859 (71) | 1,658 (59)^a^ |
| African American | 3,953 (23) | 851 (16)^a,b,c^ | 872 (22)^b^ | 1,205 (22) | 1,025 (36)^a^ |
| Primary Insurance, n (%) |  |  |  |  |  |
| Private | 4,115 (24) | 1,382 (26)^a,b,c^ | 918 (23)^b^ | 1317 (24) | 498 (18)^a^ |
| Medicare | 7,625 (44) | 2,147 (41)^a,b^ | 1,549 (39)^a,b^ | 2,539 (47) | 1,390 (49) |
| Medicaid | 3,919 (23) | 1,243 (24)^a,b,c^ | 1,075 (27)^a,b^ | 1,038 (19) | 563 (20) |
| Uninsured | 1,756 (10) | 453 (9)^a,b^ | 381 (10)^b^ | 556 (10) | 366 (13)^a^ |
| Residency area characteristics |  |  |  |  |  |
| Total Proportion of African-American (%), mean (SD) | 18.9 (17.5) | 17.4 (15.9)^b,c^ | 19.4 (17.3)^a,b^ | 18.6 (17.3) | 22.0 (20.1)^a^ |
| Population Proportion Below Poverty (%), mean (SD) | 22.7 (10.3) | 22.1 (10.3)^b,c^ | 22.8 (10.0)^a,b^ | 22.3 (10.2) | 24.2 (10.7)^a^ |
| distance from Residency to Hospital (mile), median (IQR) | 18 (3, 34) | 20 (3, 36)^a,b,c^ | 16 (3, 30)^a,b^ | 18 (3, 35) | 14 (3, 27)^a^ |
| **Comorbidities** |  |  |  |  |  |
| Hypertension, n (%) | 8,878 (51) | 2,589 (50) | 2,020 (51) | 2,813 (52) | 1,456 (52) |
| Cardiovascular disease, n (%)^d^ | 4,983 (29) | 1,485 (28) | 1,077 (27) | 1,597 (29) | 824 (29) |
| Diabetes mellitus, n (%) | 4,071 (23) | 1,198 (23) | 914 (23) | 1,290 (24) | 669 (24) |
| Chronic kidney disease, n (%) | 2,947 (17) | 769 (15)^b,c^ | 665 (17)^b^ | 901 (17) | 612 (22)^a^ |
| **Admission characteristics of patients** |  |  |  |  |  |
| Emergent Admission, n (%) | 12,542 (72) | 3,104 (59)^a,b,c^ | 3,270 (83)^a,b^ | 3,672 (67) | 2,496 (89)^a^ |
| Transfer from another hospital, n (%) | 3,595 (21) | 908 (17)^b,c^ | 983 (25)^b^ | 1,052 (19) | 652 (23)^a^ |
| **Primary admission diagnostic groups** |  |  |  |  |  |
| Diseases of the circulatory system, n (%) | 2,968 (17) | 761 (15)^a,b^ | 549 (14)^a,b^ | 1001 (18) | 657 (23)^a^ |
| Respiratory and infectious diseases, n (%) | 1,185 (7) | 229 (4)^b,c^ | 491 (13)^a,b^ | 224 (4) | 241 (9)^a^ |
| Complications of pregnancy and childbirth, n (%) | 1,366 (8) | 434 (8)^a,b,c^ | 420 (11)^a,b^ | 367 (7) | 145 (5)^a^ |
| Diseases of the digestive/genitourinary systems, n (%) | 2,201 (13) | 738 (14)^b,c^ | 428 (11)^a^ | 725 (13) | 310 (11)^a^ |
| Diseases of the musculoskeletal/connective tissue and skin, n (%) | 1,522 (9) | 567 (11)^b,c^ | 186 (5)^a,b^ | 550 (10) | 219 (8)^a^ |
| Neoplasms, n (%) | 1,136 (7) | 483 (9)^a,b,c^ | 159 (4)^a^ | 405 (7) | 89 (3)^a^ |
| **Clinical biomarkers and interventions within 24 hours of admission** |  |  |  |  |  |
| Surgery on admission day, n (%) | 3,801 (22) | 1,719 (33)^a,b,c^ | 349 (9)^a^ | 1,474 (27) | 259 (9)^a^ |
| ICU/IMC admission within first 24 hours, n (%) | 3,899 (22) | 1,129 (22)^a,c^ | 1,288 (33)^a,b^ | 881 (16) | 601 (21)^a^ |
| **Cardiovascular system** |  |  |  |  |  |
| Hypotension (MAP < 60 mmHg) at any time, n (%) | 6,014 (35) | 2,962 (57)^a,b,c^ | 1,323 (34)^a,b^ | 1,435 (26) | 294 (10)^a^ |
| Duration, median (IQR), minutes | 53 (14, 157) | 62 (19, 193)^a,b,c^ | 75 (30, 201)^a,b^ | 17 (6, 60) | 24 (6, 68) |
| Vasopressors used, n (%) | 3,294 (19) | 1,596 (31)^a,b,c^ | 424 (11)^a,b^ | 1,099 (20) | 175 (6)^a^ |
| Out of operating room | 625 (4) | 270 (5)^a,b^ | 213 (5)^a,b^ | 104 (2) | 38 (1) |
| Hypertension (SBP > 160 mmHg) at any time, n (%) | 6,222 (36) | 1,029 (20)^a,b,c^ | 655 (17)^a,b^ | 2,290 (42) | 2,248 (80)^a^ |
| Troponin, tested, n (%) | 5,862 (34) | 1,264 (24)^a,b,c^ | 1,638 (42)^a,b^ | 1,605 (29) | 1,355 (48)^a^ |
| Abnormal result among tested, n (%) | 1,239 (21) | 278 (22)^a^ | 384 (23)^a^ | 263 (16) | 314 (23)^a^ |
| **Respiratory system** |  |  |  |  |  |
| Highest administered FiO2, median (IQR) | 0.21 (0.21, 0.40) | 0.21 (0.21, 0.40)^a,b,c^ | 0.21 (0.21, 0.33)^b^ | 0.21 (0.21, 0.40) | 0.21 (0.21, 0.29)^a^ |
| Room air only, n (%) | 10,242 (59) | 2,784 (53)^a,b,c^ | 2,292 (58)^b^ | 3,278 (60) | 1,888 (67)^a^ |
| 0.22 - 0.40, n (%) | 6,125 (35) | 2,112 (40)^a,b,c^ | 1,287 (33)^b^ | 1,915 (35) | 811 (29)^a^ |
| > 0.4, n (%) | 1,048 (6) | 329 (6)^a,b,c^ | 344 (9)^a,b^ | 257 (5) | 118 (4) |
| PaO2/FiO2, tested with arterial blood gas, n (%) | 2,519 (14) | 771 (15)^a,c^ | 824 (21)^a,b^ | 554 (10) | 370 (13)^a^ |
| <200 among tested, n (%) | 908 (36) | 266 (35)^c^ | 363 (44)^a,b^ | 169 (31) | 110 (30) |
| Mechanical ventilation, n (%) | 883 (5) | 311 (6)^a,b^ | 286 (7)^a,b^ | 175 (3) | 111 (4) |
| **Kidney and acid-base status** |  |  |  |  |  |
| Preadmission estimated glomerular filtration rate^e^ (mL/min per 1.73 m2), median (IQR) | 95 (76, 111) | 97 (80, 113)^a,b,c^ | 100 (83, 117)^a,b^ | 93 (76, 107) | 88 (56, 104)^a^ |
| Highest / reference creatinine^e^ ratio, mean (SD) | 1.24 (0.74) | 1.26 (0.86)^a,c^ | 1.32 (0.86)^a,b^ | 1.15 (0.43) | 1.24 (0.78)^a^ |
| Renal replacement therapy, n (%) | 257 (1) | 53 (1)^b^ | 48 (1)^b^ | 58 (1) | 98 (3)^a^ |
| Highest Anion Gap, median (IQR), mmol/L | 14 (13, 17) | 14 (12, 16)^b,c^ | 15 (13, 18)^a,b^ | 14 (12, 16) | 15 (13, 18)^a^ |
| Arterial Blood Gas tested, n (%) | 2,521 (14) | 771 (15)^a,c^ | 825 (21)^a,b^ | 555 (10) | 370 (13)^a^ |
| pH < 7.3 among tested, n (%) | 557 (22) | 217 (28)^a,b^ | 210 (25)^a,b^ | 77 (14) | 53 (14) |
| Highest Base deficit, mean (SD), mmol/L | 4.9 (4.6) | 4.7 (4.3)^a,c^ | 6.0 (5.5)^a,b^ | 3.6 (3.1) | 4.0 (3.4) |
| Lactate, tested, n (%) | 6,237 (36) | 1,755 (34)^a,b,c^ | 1,860 (47)^a,b^ | 1,542 (28) | 1,080 (38)^a^ |
| 2 - 4 mmol/L among tested, n (%) | 1,498 (24) | 393 (22)^c^ | 533 (29)^a,b^ | 328 (21) | 244 (23) |
| > 4 mmol/L among tested, n (%) | 578 (9) | 168 (10)^a,b,c^ | 267 (14)^a,b^ | 77 (5) | 66 (6) |
| **Inflammation** |  |  |  |  |  |
| Highest White blood cell count, median (IQR), x10^9/L | 9 (7, 13) | 9 (7, 12)^a,c^ | 10 (8, 15)^a,b^ | 9 (7, 12) | 9 (7, 12) |
| Highest Premature neutrophils (bands)), median (IQR), % | 9 (3, 19) | 7 (3, 16)^c^ | 12 (4, 23)^a,b^ | 6 (2, 13) | 5 (2, 11) |
| Lowest Lymphocytes, median (IQR), % | 16 (9, 24) | 16 (9, 26)^a,c^ | 12 (6, 20)^a,b^ | 18 (11, 26) | 17 (10, 24)^a^ |
| C-reactive protein, tested, n (%) | 2,256 (13) | 597 (11)^b,c^ | 623 (16)^a^ | 619 (11) | 417 (15)^a^ |
| Highest C-reactive protein, median (IQR), mg/L | 17 (4, 80) | 16 (4, 64)^a,c^ | 53 (11, 145)^a,b^ | 10 (3, 50) | 11 (3, 50) |
| Erythrocyte sedimentation rate, tested, n (%) | 1,382 (8) | 344 (7)^b,c^ | 379 (10)^a^ | 381 (7) | 278 (10)^a^ |
| Highest Erythrocyte sedimentation rate, median (IQR), mm/h | 42 (19, 77) | 36 (16, 65)^c^ | 62 (27, 96)^a,b^ | 35 (18, 70) | 41 (21, 73) |
| Highest Temperature, mean (SD), celsius | 37.7 (0.6) | 37.7 (0.6)^a,b,c^ | 37.9 (0.7)^a,b^ | 37.6 (0.5) | 37.6 (0.6) |
| 38 - 39, n (%) | 3,563 (20) | 1,149 (22)^a,b^ | 943 (24)^a,b^ | 998 (18) | 473 (17) |
| > 39, n (%) | 604 (3) | 117 (2)^c^ | 319 (8)^a,b^ | 99 (2) | 69 (2) |
| Lowest Temperature, mean (SD), celsius | 36.7 (0.9) | 36.6 (1.1)^a,b,c^ | 36.8 (0.8)^a^ | 36.7 (0.8) | 36.8 (0.6) |
| **Hematologic** |  |  |  |  |  |
| Lowest Hemoglobin, mean (SD), g/dL | 11.4 (2.3) | 10.9 (2.3)^a,b^ | 11.0 (2.4)^a,b^ | 11.8 (2.1) | 11.9 (2.3)^a^ |
| Highest RDW, mean (SD), % | 15.2 (2.1) | 15.2 (2.1)^a,c^ | 15.6 (2.3)^a,b^ | 15.0 (1.9) | 15.2 (2.0)^a^ |
| Lowest Platelets, median (IQR), x10^9/L | 204 (157, 260) | 196 (149, 252)^a,b,c^ | 213 (159, 278)^a^ | 202 (160, 252) | 209 (165, 262)^a^ |
| Platelets < 200, n (%) | 7,489 (43) | 2,402 (46)^b,c^ | 1,632 (42)^a,b^ | 2,310 (42) | 1,145 (41)^a^ |
| < 100 | 1,128 (15) | 417 (17)^a,b^ | 319 (20)^a,b^ | 285 (12) | 107 (9) |
| 100 - 200 | 6,361 (85) | 1,985 (83)^a,b^ | 1,313 (80)^a,b^ | 2,025 (88) | 1,038 (91) |
| International normalized ratio, tested, n (%) | 7,580 (44) | 2,052 (39)^b,c^ | 1,974 (50)^a^ | 2,207 (40) | 1,347 (48)^a^ |
| >= 2 | 757 (10) | 232 (11)^a,b^ | 258 (13)^a,b^ | 171 (8) | 96 (7) |
| **Neurologic** |  |  |  |  |  |
| Glasgow Coma Scale score, n (%) |  |  |  |  |  |
| Moderate (9 - 12) | 687 (4) | 218 (4)^a^ | 190 (5)^a^ | 167 (3) | 112 (4) |
| Severe (<= 8) | 587 (3) | 189 (4)^a,c^ | 195 (5)^a,b^ | 121 (2) | 82 (3) |
| **Liver and metabolic** |  |  |  |  |  |
| Bilirubin, tested, n (%) | 8,562 (49) | 2,269 (43)^b,c^ | 2,400 (61)^a,b^ | 2,328 (43) | 1,565 (56)^a^ |
| >= 2 mg/dL, n (%) | 607 (7) | 216 (10)^a,b^ | 213 (9)^a,b^ | 126 (5) | 52 (3)^a^ |
| Highest Glucose, median (IQR), mg/dL | 126 (104, 169) | 123 (101, 160)^b,c^ | 130 (106, 178)^a^ | 123 (103, 165) | 132 (107, 185)^a^ |
| Albumin, tested, n (%) | 8,636 (50) | 2,295 (44)^b,c^ | 2,409 (61)^a,b^ | 2,355 (43) | 1,577 (56)^a^ |
| < 2.5 | 471 (5) | 158 (7)^a,b^ | 209 (9)^a,b^ | 61 (3) | 43 (3) |
| 2.5 - 3.5 | 2,665 (31) | 758 (33)^a,b,c^ | 925 (38)^a,b^ | 599 (25) | 383 (24) |

Abbreviation: ICU: intensive care unit; IMC: intermediate care unit; MAP: mean aterial pressure; RDW: red cell distribution width; SD: standard deviation; IQR: interquartile range.

All p-values were adjusted for multiple comparisons using Bonferroni method.

^a^ p < 0.05 compared to Physiotype C .

^b^ p < 0.05 compared to Physiotype D.

^c^ p < 0.05 compared to Physiotype B.

^d^ Cardiovascular disease was considered if there was a history of congestive heart failure, coronary artery disease of peripheral vascular disease.

^e^ Reference glomerular filtration rate and reference creatinine were derived without use of race correction (see S1 Text for details).
