# Supplementary material for: Physiologic signatures within six hours of hospitalization identify acute illness phenotypes
Source: PLOS Digit Health. 2022 Oct 13;1(10):e0000110. doi: 10.1371/journal.pdig.0000110 (PMC9802629; doi:10.1371/journal.pdig.0000110)
Supplement: S8 Table — (DOCX) [file pdig.0000110.s039.docx]

# S8 Table. Physiotype illness severity, clinical outcomes, and resource use in the validation cohort

| **Variables** | **Total** | **Acute Illness Physiotypes** | | | |
| --- | --- | --- | --- | --- | --- |
|  |  | Physiotype A | Physiotype B | Physiotype C | Physiotype D |
| Number of Encounters (%) | 17,415 | 5,225 (30) | 3,923 (23) | 5,450 (31) | 2,817 (16) |
| **Acuity scores within 24h of admission** |  |  |  |  |  |
| SOFA score > 6, n (%) | 1,503 (9) | 576 (11)^a,b^ | 428 (11)^a,b^ | 328 (6) | 171 (6) |
| Patients in ICU/IMC, SOFA score <= 6, n (%) | 2,795 (16) | 719 (14)^b,c^ | 921 (23)^a,b^ | 671 (12) | 484 (17)^a^ |
| Patients in ICU/IMC, SOFA score > 6, n (%) | 1,104 (6) | 410 (8)^a,b^ | 367 (9)^a,b^ | 210 (4) | 117 (4) |
| Patients in ward, SOFA score <= 6, n (%) | 13,117 (75) | 3,930 (75)^a,c^ | 2,574 (66)^a,b^ | 4,451 (82) | 2,162 (77)^a^ |
| Patients in ward, SOFA score > 6, n (%) | 399 (2) | 166 (3)^a,b,c^ | 61 (2) | 118 (2) | 54 (2) |
| MEWS score > 4, n (%) | 1,115 (6) | 156 (3)^a,b,c^ | 634 (16)^a,b^ | 103 (2) | 222 (8)^a^ |
| Patients in ICU/IMC, MEWS score <= 4, n (%) | 3,041 (17) | 999 (19)^a,b^ | 786 (20)^a,b^ | 794 (15) | 462 (16) |
| Patients in ICU/IMC, MEWS score > 4, n (%) | 858 (5) | 130 (2)^a,b,c^ | 502 (13)^a,b^ | 87 (2) | 139 (5)^a^ |
| Patients in ward, MEWS score <= 4, n (%) | 13,259 (76) | 4,070 (78)^a,c^ | 2,503 (64)^a,b^ | 4,553 (84) | 2,133 (76)^a^ |
| Patients in ward, MEWS score > 4, n (%) | 257 (1) | 26 (0)^b,c^ | 132 (3)^a^ | 16 (0) | 83 (3)^a^ |
| **Resource use during hospitalization** |  |  |  |  |  |
| Hospital days, median (IQR) | 4 (2, 7) | 4 (2, 6)^a,c^ | 4 (3, 8)^a,b^ | 3 (2, 6) | 4 (2, 7)^a^ |
| Surgery at any time, n (%) | 5,084 (29) | 2,032 (39)^a,b,c^ | 668 (17)^a^ | 1,860 (34) | 524 (19)^a^ |
| Admitted to ICU/IMC^d^, n (%) | 4,643 (27) | 1,332 (25)^a,c^ | 1,488 (38)^a,b^ | 1,074 (20) | 749 (27)^a^ |
| Days in ICU/IMC^e^, median (IQR) | 4 (2, 7) | 4 (2, 7)^c^ | 4 (3, 8)^a,b^ | 4 (2, 6) | 4 (2, 6) |
| Days in ICU/IMC greater than 48 hrs, n (%) | 3,468 (75) | 976 (73) | 1,146 (77) | 787 (73) | 559 (75) |
| Mechanical Ventilation, n (%) | 1,403 (8) | 446 (9)^a,b,c^ | 459 (12)^a,b^ | 313 (6) | 185 (7) |
| Mechanical Ventilation hours, median (IQR)^f^ | 31 (11, 105) | 24 (9, 79)^c^ | 46 (18, 130)^a^ | 19 (8, 85) | 42 (12, 141)^a^ |
| Mechanical Ventilation greater than 2 calendar days, n (%) | 699 (50) | 200 (45)^c^ | 274 (60)^a,b^ | 124 (40) | 101 (55) |
| Renal replacement therapy, n (%) | 524 (3) | 128 (2)^b^ | 129 (3)^a,b^ | 106 (2) | 161 (6)^a^ |
| **Complications** |  |  |  |  |  |
| Acute kidney injury overall, n (%) | 2,901 (17) | 846 (16)^a,c^ | 868 (22)^a,b^ | 667 (12) | 520 (18)^a^ |
| Community-acquired AKI, n (%) | 1,603 (55) | 498 (59)^a^ | 506 (58)^a^ | 328 (49) | 271 (52) |
| Hospital-acquired AKI, n (%) | 1,298 (45) | 348 (41)^a^ | 362 (42)^a^ | 339 (51) | 249 (48) |
| Worst AKI staging, n (%) |  |  |  |  |  |
| Stage 1 | 1,878 (65) | 522 (62)^a^ | 521 (60)^a,b^ | 477 (72) | 358 (69) |
| Stage 2 | 533 (18) | 171 (20) | 169 (19) | 112 (17) | 81 (16) |
| Stage 3 | 348 (12) | 114 (13)^a^ | 128 (15)^a^ | 54 (8) | 52 (10) |
| Stage 3 with RRT | 142 (5) | 39 (5) | 50 (6) | 24 (4) | 29 (6) |
| Venous Thromboembolism, n (%) | 708 (4) | 166 (3)^b,c^ | 224 (6)^a^ | 177 (3) | 141 (5)^a^ |
| Sepsis, n (%) | 1,659 (10) | 421 (8)^a,c^ | 861 (22)^a,b^ | 190 (3) | 187 (7)^a^ |
| Hospital disposition, n (%) |  |  |  |  |  |
| Hospital mortality | 480 (3) | 127 (2)^a,c^ | 198 (5)^a,b^ | 84 (2) | 71 (3)^a^ |
| Another hospital, LTAC, SNF, Hospice | 2,002 (11) | 560 (11)^c^ | 533 (14)^a^ | 552 (10) | 357 (13)^a^ |
| Home or short-term rehabilitation | 14,933 (86) | 4,538 (87)^c^ | 3,192 (81)^a,b^ | 4,814 (88) | 2,389 (85)^a^ |
| Thirty-day mortality, n (%) | 646 (4) | 166 (3)^a,c^ | 263 (7)^a,b^ | 120 (2) | 97 (3)^a^ |
| Three-year mortality, n (%) | 3,297 (19) | 913 (17)^a,c^ | 1,030 (26)^a,b^ | 831 (15) | 523 (19)^a^ |

Abbreviation: SOFA: sequential organ failure assessment; MEWS: modified early warning score; ICU: intensive care unit; IMC: intermediate care unit; IQR: interquartile range.

All p-values were adjusted for multiple comparisons using Bonferroni method.

^a^ p < 0.05 compared to Physiotype C .

^b^ p < 0.05 compared to Physiotype D.

^c^ p < 0.05 compared to Physiotype B.

^d^ At any time during hospitalization.

^e^ Values were calculated among patients admitted to ICU/IMC.

^f^ Values were calculated among patients requiring MV.
