# Supplementary material for: Physiologic signatures within six hours of hospitalization identify acute illness phenotypes
Source: PLOS Digit Health. 2022 Oct 13;1(10):e0000110. doi: 10.1371/journal.pdig.0000110 (PMC9802629; doi:10.1371/journal.pdig.0000110)
Supplement: S9 Table — (DOCX) [file pdig.0000110.s040.docx]

# S9 Table. Physiotype clinical characteristics and biomarkers in sensitivity analysis by excluding highly missing variable (Temperature) in the training cohort

| **Variables** | **Total** | **Acute Illness Physiotypes** | | | |
| --- | --- | --- | --- | --- | --- |
|  |  | Physiotype A | Physiotype B | Physiotype C | Physiotype D |
| Number of Encounters (%) | 41,502 | 12,748 (31) | 8,240 (20) | 14,011 (34) | 6,503 (16) |
| **Preadmission clinical characteristics** |  |  |  |  |  |
| Age, mean (SD) | 54 (19) | 52 (18)^a,b,c^ | 50 (19)^a,b^ | 56 (18) | 59 (17)^a^ |
| Female sex, n (%) | 22,745 (55) | 7,334 (58)^a,b^ | 4,686 (57)^a,b^ | 7,264 (52) | 3,461 (53) |
| Race, n (%) |  |  |  |  |  |
| White | 29,076 (70) | 9,513 (75)^a,b,c^ | 5,667 (69)^a,b^ | 9,973 (71) | 3923 (60)^a^ |
| African American | 9,634 (23) | 2,192 (17)^a,b,c^ | 2,042 (25)^a,b^ | 3,141 (22) | 2259 (35)^a^ |
| Primary Insurance, n (%) |  |  |  |  |  |
| Private | 9591 (23) | 3240 (25)^a,b,c^ | 1932 (23)^b^ | 3302 (24) | 1117 (17)^a^ |
| Medicare | 18499 (45) | 5288 (41)^a,b,c^ | 3201 (39)^a,b^ | 6545 (47) | 3465 (53)^a^ |
| Medicaid | 9231 (22) | 2989 (23)^a,b,c^ | 2227 (27)^a,b^ | 2780 (20) | 1235 (19) |
| Uninsured | 4181 (10) | 1231 (10) | 880 (11) | 1384 (10) | 686 (11) |
| Residency area characteristics |  |  |  |  |  |
| Total Proportion of African-American (%), mean (SD) | 18.7 (17.5) | 17.4 (16.2)^a,b,c^ | 19.5 (17.8)^a,b^ | 18.6 (17.4) | 20.7 (19.2)^a^ |
| Population Proportion Below Poverty (%), mean (SD) | 22.7 (10.1) | 22.0 (9.9)^a,b,c^ | 23.4 (10.0)^a^ | 22.5 (10.0) | 23.7 (10.4)^a^ |
| distance from Residency to Hospital (mile), median (IQR) | 18 (3, 34) | 22 (3, 36)^a,b,c^ | 14 (3, 31)^a,b^ | 18 (3, 36) | 14 (3, 27)^a^ |
| **Comorbidities** |  |  |  |  |  |
| Hypertension, n (%) | 21639 (52) | 6526 (51)^b^ | 4240 (51)^b^ | 7250 (52) | 3623 (56)^a^ |
| Cardiovascular disease, n (%)^d^ | 12058 (29) | 3517 (28)^b^ | 2383 (29)^b^ | 4032 (29) | 2126 (33)^a^ |
| Diabetes mellitus, n (%) | 10111 (24) | 2946 (23)^b^ | 2001 (24)^b^ | 3356 (24) | 1808 (28)^a^ |
| Chronic kidney disease, n (%) | 6518 (16) | 1728 (14)^a,b^ | 1224 (15)^b^ | 2141 (15) | 1425 (22)^a^ |
| **Admission characteristics of patients** |  |  |  |  |  |
| Emergent Admission, n (%) | 30177 (73) | 7824 (61)^a,b,c^ | 6915 (84)^a,b^ | 9640 (69) | 5798 (89)^a^ |
| Transfer from another hospital, n (%) | 7115 (17) | 2047 (16)^b,c^ | 1649 (20)^a^ | 2221 (16) | 1198 (18)^a^ |
| **Primary admission diagnostic groups** |  |  |  |  |  |
| Diseases of the circulatory system, n (%) | 7719 (19) | 2122 (17)^a,b^ | 1270 (15)^a,b^ | 2621 (19) | 1706 (26)^a^ |
| Respiratory and infectious diseases, n (%) | 3306 (8) | 633 (5)^b,c^ | 1239 (15)^a,b^ | 785 (6) | 649 (10)^a^ |
| Complications of pregnancy and childbirth, n (%) | 3148 (8) | 1008 (8)^a,b,c^ | 935 (11)^a,b^ | 938 (7) | 267 (4)^a^ |
| Diseases of the digestive/genitourinary systems, n (%) | 5184 (12) | 1831 (14)^a,b,c^ | 841 (10)^a^ | 1830 (13) | 682 (10)^a^ |
| Diseases of the musculoskeletal/connective tissue and skin, n (%) | 3651 (9) | 1361 (11)^b,c^ | 400 (5)^a,b^ | 1391 (10) | 499 (8)^a^ |
| Neoplasms, n (%) | 2743 (7) | 1123 (9)^b,c^ | 308 (4)^a^ | 1117 (8) | 195 (3)^a^ |
| **Clinical biomarkers and interventions within 24 hours of admission** |  |  |  |  |  |
| Surgery on admission day, n (%) | 8644 (21) | 3927 (31)^a,b,c^ | 655 (8)^a^ | 3517 (25) | 545 (8)^a^ |
| ICU/IMC admission within first 24 hours, n (%) | 9426 (23) | 2914 (23)^a,c^ | 2582 (31)^a,b^ | 2487 (18) | 1443 (22)^a^ |
| **Cardiovascular system** |  |  |  |  |  |
| Hypotension (MAP < 60 mmHg) at any time, n (%) | 14470 (35) | 7267 (57)^a,b,c^ | 2682 (33)^a,b^ | 3801 (27) | 720 (11)^a^ |
| Duration, median (IQR), minutes | 57 (15, 168) | 70 (21, 219)^a,b^ | 68 (28, 190)^a,b^ | 21 (6, 70) | 30 (8, 78) |
| Vasopressors used, n (%) | 7531 (18) | 3700 (29)^a,b,c^ | 789 (10)^a,b^ | 2633 (19) | 409 (6)^a^ |
| Out of operating room | 1403 (3) | 696 (5)^a,b,c^ | 376 (5)^a,b^ | 253 (2) | 78 (1)^a^ |
| Hypertension (SBP > 160 mmHg) at any time, n (%) | 14838 (36) | 2167 (17)^a,b^ | 1346 (16)^a,b^ | 5764 (41) | 5561 (86)^a^ |
| Troponin, tested, n (%) | 14616 (35) | 3362 (26)^a,b,c^ | 3533 (43)^a,b^ | 4327 (31) | 3394 (52)^a^ |
| Abnormal result among tested, n (%) | 3398 (23) | 862 (26)^a^ | 825 (23)^a^ | 848 (20) | 863 (25)^a^ |
| **Respiratory system** |  |  |  |  |  |
| Highest administered FiO2, median (IQR) | 0.21 (0.21, 0.40) | 0.21 (0.21, 0.40)^a,b,c^ | 0.21 (0.21, 0.33)^b^ | 0.21 (0.21, 0.40) | 0.21 (0.21, 0.29)^a^ |
| Room air only, n (%) | 23963 (58) | 6668 (52)^a,b,c^ | 4713 (57)^a,b^ | 8365 (60) | 4217 (65)^a^ |
| 0.22 - 0.40, n (%) | 14790 (36) | 5146 (40)^a,b,c^ | 2788 (34)^b^ | 4890 (35) | 1966 (30)^a^ |
| > 0.4, n (%) | 2749 (7) | 934 (7)^a,b,c^ | 739 (9)^a,b^ | 756 (5) | 320 (5) |
| PaO2/FiO2, tested with arterial blood gas, n (%) | 6113 (15) | 1952 (15)^a,b,c^ | 1709 (21)^a,b^ | 1612 (12) | 840 (13)^a^ |
| <200 among tested, n (%) | 2265 (37) | 746 (38)^a^ | 719 (42)^a,b^ | 522 (32) | 278 (33) |
| Mechanical ventilation, n (%) | 2123 (5) | 799 (6)^a,b^ | 548 (7)^a,b^ | 547 (4) | 229 (4) |
| **Kidney and acid-base status** |  |  |  |  |  |
| Preadmission estimated glomerular filtration rate^e^ (mL/min per 1.73 m2), median (IQR) | 95 (78, 111) | 98 (81, 113)^a,b,c^ | 100 (83, 117)^a,b^ | 93 (78, 108) | 87 (53, 103)^a^ |
| Highest / reference creatinine^e^ ratio, mean (SD) | 1.24 (0.66) | 1.27 (0.74)^a,b,c^ | 1.29 (0.68)^a,b^ | 1.18 (0.56) | 1.24 (0.66)^a^ |
| Renal replacement therapy, n (%) | 641 (2) | 163 (1)^b^ | 87 (1)b | 149 (1) | 242 (4)^a^ |
| Highest Anion Gap, median (IQR), mmol/L | 14 (12, 17) | 14 (11, 16)^b,c^ | 15 (12, 18)^a,b^ | 14 (12, 16) | 15 (12, 17)^a^ |
| Arterial Blood Gas tested, n (%) | 6115 (15) | 1954 (15)^a,b,c^ | 1709 (21)^a,b^ | 1612 (12) | 840 (13)^a^ |
| pH < 7.3 among tested, n (%) | 1437 (23) | 554 (28)^a,b^ | 467 (27)^a,b^ | 287 (18) | 129 (15) |
| Highest Base deficit, mean (SD), mmol/L | 4.8 (4.7) | 4.8 (4.5)^a,c^ | 6.3 (5.8)^a,b^ | 3.7 (3.3) | 4.1 (3.6) |
| Lactate, tested, n (%) | 15447 (37) | 4418 (35)^a,b,c^ | 4007 (49)^a,b^ | 4459 (32) | 2563 (39)^a^ |
| 2 - 4 mmol/L among tested, n (%) | 3739 (24) | 1031 (23)^c^ | 1155 (29)^a,b^ | 978 (22) | 575 (22) |
| > 4 mmol/L among tested, n (%) | 1374 (9) | 413 (9)^a,b,c^ | 525 (13)^a,b^ | 258 (6) | 178 (7) |
| **Inflammation** |  |  |  |  |  |
| Highest White blood cell count, median (IQR), x10^9/L | 9 (7, 13) | 9 (7, 13)^a,b,c^ | 10 (8, 15)^a,b^ | 9 (7, 12) | 9 (7, 12)^a^ |
| Highest Premature neutrophils (bands)), median (IQR), % | 10 (4, 20) | 10 (4, 19)^a,b,c^ | 12 (5, 24)^a,b^ | 6 (3, 15) | 7 (3, 15) |
| Lowest Lymphocytes, median (IQR), % | 16 (9, 24) | 16 (8, 25)^a,b,c^ | 12 (6, 20)^a,b^ | 17 (10, 26) | 17 (10, 24)^a^ |
| C-reactive protein, tested, n (%) | 5862 (14) | 1613 (13)^b,c^ | 1454 (18)^a,b^ | 1773 (13) | 1022 (16)^a^ |
| Highest C-reactive protein, median (IQR), mg/L | 18 (5, 77) | 19 (5, 76)^a,b,c^ | 51 (11, 122)^a,b^ | 12 (4, 56) | 13 (4, 57) |
| Erythrocyte sedimentation rate, tested, n (%) | 3903 (9) | 1047 (8)^b,c^ | 866 (11)^a^ | 1270 (9) | 720 (11)^a^ |
| Highest Erythrocyte sedimentation rate, median (IQR), mm/h | 40 (19, 73) | 37 (17, 68)^b,c^ | 49 (24, 87)^a^ | 34 (17, 66) | 41 (20, 75)^a^ |
| Highest Temperature, mean (SD), celsius | 37.7 (0.6) | 37.7 (0.6)^a,b,c^ | 37.9 (0.8)^a,b^ | 37.6 (0.5) | 37.7 (0.6) |
| 38 - 39, n (%) | 8633 (21) | 2827 (22)^a,b,c^ | 1995 (24)^a,b^ | 2611 (19) | 1200 (18) |
| > 39, n (%) | 1548 (4) | 398 (3)^a,c^ | 716 (9)^a,b^ | 262 (2) | 172 (3)^a^ |
| Lowest Temperature, mean (SD), celsius | 36.7 (1.0) | 36.5 (1.3)^a,b,c^ | 36.8 (0.7)^a^ | 36.7 (0.9) | 36.8 (0.7)^a^ |
| **Hematologic** |  |  |  |  |  |
| Lowest Hemoglobin, mean (SD), g/dL | 11.5 (2.3) | 11.1 (2.3)^a,b,c^ | 11.3 (2.4)^a,b^ | 11.9 (2.2) | 12.0 (2.3) |
| Highest RDW, mean (SD), % | 15.5 (2.1) | 15.6 (2.3)^a,b,c^ | 15.8 (2.3)^a,b^ | 15.3 (2.0) | 15.5 (1.938)^a^ |
| Lowest Platelets, median (IQR), x10^9/L | 210 (161, 269) | 201 (152, 260)^a,b,c^ | 219 (164, 286)^a^ | 211 (166, 266) | 216 (168, 271)^a^ |
| Platelets < 200, n (%) | 16707 (40) | 5563 (44)^a,b,c^ | 3227 (39)^a,b^ | 5396 (39) | 2521 (39)^a^ |
| < 100 | 2643 (16) | 1028 (18)^a,b^ | 642 (20)^a,b^ | 695 (13) | 278 (11) |
| 100 - 200 | 14064 (84) | 4535 (82)^a,b^ | 2585 (80)^a,b^ | 4701 (87) | 2243 (89) |
| International normalized ratio, tested, n (%) | 20357 (49) | 5776 (45)^b,c^ | 4389 (53)^a,b^ | 6462 (46) | 3730 (57)^a^ |
| >= 2 | 1836 (9) | 651 (11)^a,b^ | 483 (11)^a,b^ | 475 (7) | 227 (6) |
| **Neurologic** |  |  |  |  |  |
| Glasgow Coma Scale score, n (%) |  |  |  |  |  |
| Moderate (9 - 12) | 1708 (4) | 609 (5)^a,b^ | 408 (5)^a,b^ | 476 (3) | 215 (3) |
| Severe (<= 8) | 1482 (4) | 479 (4)^a,c^ | 398 (5)^a,b^ | 404 (3) | 201 (3) |
| **Liver and metabolic** |  |  |  |  |  |
| Bilirubin, tested, n (%) | 21183 (51) | 5798 (45)^b,c^ | 5009 (61)^a^ | 6466 (46) | 3910 (60)^a^ |
| >= 2 mg/dL, n (%) | 1427 (7) | 589 (10)^a,b,c^ | 387 (8)^a,b^ | 328 (5) | 123 (3)^a^ |
| Highest Glucose, median (IQR), mg/dL | 126 (104, 170) | 122 (101, 161)^a,b,c^ | 129 (106, 175)^a,b^ | 125 (103, 168) | 134 (107, 190)^a^ |
| Albumin, tested, n (%) | 21368 (51) | 5871 (46)^b,c^ | 5032 (61)^a^ | 6532 (47) | 3933 (60)^a^ |
| < 2.5 | 1243 (6) | 484 (8)^a,b^ | 434 (9)^a,b^ | 216 (3) | 109 (3) |
| 2.5 - 3.5 | 6904 (32) | 2053 (35)^a,b,c^ | 1935 (38)^a,b^ | 1784 (27) | 1132 (29) |

Abbreviation: ICU: intensive care unit; IMC: intermediate care unit; MAP: mean aterial pressure; RDW: red cell distribution width; SD: standard deviation; IQR: interquartile range.

All p-values were adjusted for multiple comparisons using Bonferroni method.

^a^ p < 0.05 compared to Physiotype C .

^b^ p < 0.05 compared to Physiotype D.

^c^ p < 0.05 compared to Physiotype B.

^d^ Cardiovascular disease was considered if there was a history of congestive heart failure, coronary artery disease of peripheral vascular disease.

^e^ Reference glomerular filtration rate and reference creatinine were derived without use of race correction (see S1 Text for details).
