# Supplementary material for: Physiologic signatures within six hours of hospitalization identify acute illness phenotypes
Source: PLOS Digit Health. 2022 Oct 13;1(10):e0000110. doi: 10.1371/journal.pdig.0000110 (PMC9802629; doi:10.1371/journal.pdig.0000110)
Supplement: S10 Table — (DOCX) [file pdig.0000110.s041.docx]

# S10 Table. Physiotype illness severity, clinical outcomes, and resource use in sensitivity analysis by excluding highly missing variable (Temperature) in the training cohort

| **Variables** | **Total** | **Acute Illness Physiotypes** | | | |
| --- | --- | --- | --- | --- | --- |
|  |  | Physiotype A | Physiotype B | Physiotype C | Physiotype D |
| Number of Encounters (%) | 41502 | 12748 (31) | 8240 (20) | 14011 (34) | 6503 (16) |
| **Acuity scores within 24h of admission** |  |  |  |  |  |
| SOFA score > 6, n (%) | 3506 (8) | 1520 (12)^a,b,c^ | 767 (9)^a,b^ | 866 (6) | 353 (5) |
| Patients in ICU/IMC, SOFA score <= 6, n (%) | 6882 (17) | 1844 (14)^b,c^ | 1924 (23)^a,b^ | 1927 (14) | 1187 (18)^a^ |
| Patients in ICU/IMC, SOFA score > 6, n (%) | 2544 (6) | 1070 (8)^a,b^ | 658 (8)^a,b^ | 560 (4) | 256 (4) |
| Patients in ward, SOFA score <= 6, n (%) | 31114 (75) | 9384 (74)^a,b,c^ | 5549 (67)^a,b^ | 11218 (80) | 4963 (76)^a^ |
| Patients in ward, SOFA score > 6, n (%) | 962 (2) | 450 (4)^a,b,c^ | 109 (1)^a^ | 306 (2) | 97 (1)^a^ |
| MEWS score > 4, n (%) | 2828 (7) | 594 (5)^a,b,c^ | 1368 (17)^a,b^ | 313 (2) | 553 (9)^a^ |
| Patients in ICU/IMC, MEWS score <= 4, n (%) | 7235 (17) | 2418 (19)^a,b^ | 1501 (18)^a^ | 2240 (16) | 1076 (17) |
| Patients in ICU/IMC, MEWS score > 4, n (%) | 2191 (5) | 496 (4)^a,b,c^ | 1081 (13)^a,b^ | 247 (2) | 367 (6)^a^ |
| Patients in ward, MEWS score <= 4, n (%) | 31439 (76) | 9736 (76)^a,c^ | 5371 (65)^a,b^ | 11458 (82) | 4874 (75)^a^ |
| Patients in ward, MEWS score > 4, n (%) | 637 (2) | 98 (1)^a,b,c^ | 287 (3)^a^ | 66 (0) | 186 (3)^a^ |
| **Resource use during hospitalization** |  |  |  |  |  |
| Hospital days, median (IQR) | 4 (2, 7) | 4 (2, 7)^a,c^ | 4 (3, 8)^a,b^ | 3 (2, 6) | 4 (2, 7)^a^ |
| Surgery at any time, n (%) | 11634 (28) | 4757 (37)^a,b,c^ | 1224 (15)^a^ | 4596 (33) | 1057 (16)^a^ |
| Admitted to ICU/IMC^d^, n (%) | 11121 (27) | 3374 (26)^a,c^ | 2986 (36)^a,b^ | 3006 (21) | 1755 (27)^a^ |
| Days in ICU/IMC^e^, median (IQR) | 4 (2, 7) | 4 (3, 7)^a,b^ | 4 (3, 8)^a,b^ | 4 (2, 7) | 4 (2, 6) |
| Days in ICU/IMC greater than 48 hrs, n (%) | 8332 (75) | 2579 (76)^a^ | 2319 (78)^a^ | 2129 (71) | 1305 (74) |
| Mechanical Ventilation, n (%) | 3218 (8) | 1145 (9)^a,b,c^ | 849 (10)^a,b^ | 868 (6) | 356 (5) |
| Mechanical Ventilation hours, median (IQR)^f^ | 35 (14, 113) | 26 (12, 86)^b,c^ | 47 (17, 141)^a^ | 28 (13, 105) | 52 (21, 146)^a^ |
| Mechanical Ventilation greater than 2 calendar days, n (%) | 1661 (52) | 523 (46)^b,c^ | 501 (59)^a^ | 418 (48) | 219 (62)^a^ |
| Renal replacement therapy, n (%) | 1262 (3) | 343 (3)^a,b^ | 231 (3)^a,b^ | 303 (2) | 385 (6)^a^ |
| **Complications** |  |  |  |  |  |
| Acute kidney injury overall, n (%) | 6905 (17) | 2062 (16)^a,b,c^ | 1765 (21)^a,b^ | 1853 (13) | 1225 (19)^a^ |
| Community-acquired AKI, n (%) | 3839 (56) | 1302 (63)^a,b,c^ | 990 (56)^b^ | 977 (53) | 570 (47)^a^ |
| Hospital-acquired AKI, n (%) | 3066 (44) | 760 (37)^a,b,c^ | 775 (44)^b^ | 876 (47) | 655 (53)^a^ |
| Worst AKI staging, n (%) |  |  |  |  |  |
| Stage 1 | 4360 (63) | 1219 (59)^a,b^ | 1058 (60)^a,b^ | 1273 (69) | 810 (66) |
| Stage 2 | 1362 (20) | 434 (21)^a,b^ | 396 (22)^a,b^ | 322 (17) | 210 (17) |
| Stage 3 | 848 (12) | 297 (14)^a^ | 214 (12) | 194 (10) | 143 (12) |
| Stage 3 with RRT | 335 (5) | 112 (5)^a^ | 97 (5)^a^ | 64 (3) | 62 (5) |
| Venous Thromboembolism, n (%) | 1257 (3) | 366 (3)^c^ | 341 (4)^a,b^ | 380 (3) | 170 (3) |
| Sepsis, n (%) | 3750 (9) | 1102 (9)^a,b,c^ | 1631 (20)^a,b^ | 581 (4) | 436 (7)^a^ |
| Hospital disposition, n (%) |  |  |  |  |  |
| Hospital mortality | 1141 (3) | 348 (3)^a,c^ | 406 (5)^a,b^ | 250 (2) | 137 (2) |
| Another hospital, LTAC, SNF, Hospice | 4475 (11) | 1270 (10)^b,c^ | 1046 (13)^a^ | 1403 (10) | 756 (12)^a^ |
| Home or short-term rehabilitation | 35886 (86) | 11130 (87)^c^ | 6788 (82)^a,b^ | 12358 (88) | 5610 (86)^a^ |
| Thirty-day mortality, n (%) | 1633 (4) | 493 (4)^a,c^ | 562 (7)^a,b^ | 370 (3) | 208 (3) |
| Three-year mortality, n (%) | 8013 (19) | 2316 (18)^a,b,c^ | 2042 (25)^a,b^ | 2279 (16) | 1376 (21)^a^ |

Abbreviation: SOFA: sequential organ failure assessment; MEWS: modified early warning score; ICU: intensive care unit; IMC: intermediate care unit; IQR: interquartile range.

All p-values were adjusted for multiple comparisons using Bonferroni method.

^a^ p < 0.05 compared to Physiotype C .

^b^ p < 0.05 compared to Physiotype D.

^c^ p < 0.05 compared to Physiotype B.

^d^ At any time during hospitalization.

^e^ Values were calculated among patients admitted to ICU/IMC.

^f^ Values were calculated among patients requiring MV.
