# Supplementary material for: Physiologic signatures within six hours of hospitalization identify acute illness phenotypes
Source: PLOS Digit Health. 2022 Oct 13;1(10):e0000110. doi: 10.1371/journal.pdig.0000110 (PMC9802629; doi:10.1371/journal.pdig.0000110)
Supplement: S11 Table — (DOCX) [file pdig.0000110.s042.docx]

# S11 Table. Physiotype clinical characteristics and biomarkers in sensitivity analysis by excluding variables with high missingness (temperature) and correlation (diastolic blood pressure and respiratory rate) in the training cohort

| **Variables** | **Total** | **Acute Illness Physiotypes** | | | |
| --- | --- | --- | --- | --- | --- |
|  |  | Physiotype A | Physiotype B | Physiotype C | Physiotype D |
| Number of Encounters (%) | 41,502 | 12,544 (30) | 10,013 (24) | 12,874 (31) | 6,071 (15) |
| **Preadmission clinical characteristics** |  |  |  |  |  |
| Age, mean (SD) | 54 (19) | 51 (18)^a,b,c^ | 49 (19)^a,b^ | 57 (18) | 61 (17)^a^ |
| Female sex, n (%) | 22,745 (55) | 7,119 (57)^a^ | 5,715 (57)^a^ | 6,552 (51) | 3,359 (55)^a^ |
| Race, n (%) |  |  |  |  |  |
| White | 29,076 (70) | 9,216 (73)^a,b,c^ | 6,927 (69)^a,b^ | 9,183 (71) | 3,750 (62)^a^ |
| African American | 9,634 (23) | 2,298 (18)^a,b,c^ | 2,461 (25)^a,b^ | 2,854 (22) | 2,021 (33)^a^ |
| Primary Insurance, n (%) |  |  |  |  |  |
| Private | 9,591 (23) | 3,259 (26)^a,b^ | 2,544 (25)^a,b^ | 2,822 (22) | 966 (16)^a^ |
| Medicare | 18,499 (45) | 4,938 (39)^a,b,c^ | 3,697 (37)^a,b^ | 6,402 (50) | 3,462 (57)^a^ |
| Medicaid | 9,231 (22) | 3,084 (25)^a,b,c^ | 2,668 (27)^a,b^ | 2,437 (19) | 1,042 (17)^a^ |
| Uninsured | 4,181 (10) | 1,263 (10) | 1,104 (11)^a^ | 1,213 (9) | 601 (10) |
| Residency area characteristics |  |  |  |  |  |
| Total Proportion of African-American (%), mean (SD) | 18.7 (17.5) | 17.8 (16.4)^b,c^ | 19.4 (17.8)^a^ | 18.4 (17.3) | 20.3 (19.1)^a^ |
| Population Proportion Below Poverty (%), mean (SD) | 22.7 (10.1) | 22.3 (9.9)^b,c^ | 23.2 (10.0)^a^ | 22.4 (10.0) | 23.5 (10.3)^a^ |
| distance from Residency to Hospital (mile), median (IQR) | 18 (3, 34) | 20 (3, 36)^a,b,c^ | 14 (3, 32)^a,b^ | 18 (3, 36) | 14 (3, 27)^a^ |
| **Comorbidities** |  |  |  |  |  |
| Hypertension, n (%) | 21,639 (52) | 6,405 (51)^b^ | 5,170 (52)^b^ | 6,705 (52) | 3,359 (55)^a^ |
| Cardiovascular disease, n (%)^d^ | 12,058 (29) | 3,468 (28)^b^ | 2,842 (28)^b^ | 3,739 (29) | 2,009 (33)^a^ |
| Diabetes mellitus, n (%) | 10,111 (24) | 2,846 (23)^b,c^ | 2,477 (25)^b^ | 3,099 (24) | 1,689 (28)^a^ |
| Chronic kidney disease, n (%) | 6,518 (16) | 1,680 (13)^a,b^ | 1,419 (14)^a,b^ | 2,045 (16) | 1,374 (23)^a^ |
| **Admission characteristics of patients** |  |  |  |  |  |
| Emergent Admission, n (%) | 30,177 (73) | 7,941 (63)^a,b,c^ | 8,067 (81)^a,b^ | 8,776 (68) | 5,393 (89)^a^ |
| Transfer from another hospital, n (%) | 7,115 (17) | 2,004 (16)^b,c^ | 1,933 (19)^a^ | 2,041 (16) | 1,137 (19)^a^ |
| **Primary admission diagnostic groups** |  |  |  |  |  |
| Diseases of the circulatory system, n (%) | 7,719 (19) | 2,074 (17)^a,b,c^ | 1,411 (14)^a,b^ | 2,632 (20) | 1,602 (26)^a^ |
| Respiratory and infectious diseases, n (%) | 3,306 (8) | 688 (5)^b,c^ | 1,259 (13)^a,b^ | 790 (6) | 569 (9)^a^ |
| Complications of pregnancy and childbirth, n (%) | 3,148 (8) | 1,085 (9)^a,b,c^ | 1,066 (11)^a,b^ | 805 (6) | 192 (3)^a^ |
| Diseases of the digestive/genitourinary systems, n (%) | 5,184 (12) | 1,785 (14)^a,b,c^ | 1,094 (11)^a^ | 1,623 (13) | 682 (11)^a^ |
| Diseases of the musculoskeletal/connective tissue and skin, n (%) | 3,651 (9) | 1,284 (10)^b,c^ | 602 (6)^a,b^ | 1,285 (10) | 480 (8)^a^ |
| Neoplasms, n (%) | 2,743 (7) | 1,030 (8)^b,c^ | 492 (5)^a,b^ | 1,018 (8) | 203 (3)^a^ |
| **Clinical biomarkers and interventions within 24 hours of admission** |  |  |  |  |  |
| Surgery on admission day, n (%) | 8,644 (21) | 3,480 (28)^a,b,c^ | 1,260 (13)^a,b^ | 3,310 (26) | 594 (10)^a^ |
| ICU/IMC admission within first 24 hours, n (%) | 9,426 (23) | 2,644 (21)^a,c^ | 3,006 (30)^a,b^ | 2,415 (19) | 1,361 (22)^a^ |
| **Cardiovascular system** |  |  |  |  |  |
| Hypotension (MAP < 60 mmHg) at any time, n (%) | 14,470 (35) | 6,588 (53)^a,b,c^ | 3,273 (33)^a,b^ | 3,826 (30) | 783 (13)^a^ |
| Duration, median (IQR), minutes | 57 (15, 168) | 69 (21, 220)^a,b,c^ | 60 (19, 180)^a,b^ | 27 (7, 85) | 30 (8, 88) |
| Vasopressors used, n (%) | 7,531 (18) | 3,251 (26)^a,b,c^ | 1,267 (13)^a,b^ | 2,570 (20) | 443 (7)^a^ |
| Out of operating room | 1,403 (3) | 614 (5)^a,b^ | 458 (5)^a,b^ | 251 (2) | 80 (1)^a^ |
| Hypertension (SBP > 160 mmHg) at any time, n (%) | 14,838 (36) | 1,835 (15)^a,b,c^ | 2,126 (21)^a,b^ | 5,456 (42) | 5,421 (89)^a^ |
| Troponin, tested, n (%) | 14,616 (35) | 3,423 (27)^a,b,c^ | 3,818 (38)^a,b^ | 4,206 (33) | 3,169 (52)^a^ |
| Abnormal result among tested, n (%) | 3,398 (23) | 847 (25)^a^ | 877 (23)^a,b^ | 856 (20) | 818 (26)^a^ |
| **Respiratory system** |  |  |  |  |  |
| Highest administered FiO2, median (IQR) | 0.21 (0.21, 0.40) | 0.21 (0.21, 0.40)^a,b,c^ | 0.21 (0.21, 0.37)^b^ | 0.21 (0.21, 0.40) | 0.21 (0.21, 0.29)^a^ |
| Room air only, n (%) | 23,963 (58) | 6,913 (55)^a,b,c^ | 5,771 (58)^b^ | 7,417 (58) | 3,862 (64)^a^ |
| 0.22 - 0.40, n (%) | 14,790 (36) | 4,810 (38)^a,b,c^ | 3,385 (34)^a,b^ | 4,685 (36) | 1,910 (31)^a^ |
| > 0.4, n (%) | 2,749 (7) | 821 (7)^b,c^ | 857 (9)^a,b^ | 772 (6) | 299 (5)^a^ |
| PaO2/FiO2, tested with arterial blood gas, n (%) | 6,113 (15) | 1,714 (14)^c^ | 1,992 (20)^a,b^ | 1,630 (13) | 777 (13) |
| <200 among tested, n (%) | 2,265 (37) | 673 (39)^a,b^ | 788 (40)^a,b^ | 550 (34) | 254 (33) |
| Mechanical ventilation, n (%) | 2,123 (5) | 646 (5)^a,b,c^ | 735 (7)^a,b^ | 514 (4) | 228 (4) |
| **Kidney and acid-base status** |  |  |  |  |  |
| Preadmission estimated glomerular filtration rate^e^ (mL/min per 1.73 m2), median (IQR) | 95 (78, 111) | 99 (82, 114)^a,b,c^ | 101 (85, 118)^a,b^ | 92 (76, 106) | 84 (49, 100)^a^ |
| Highest / reference creatinine^e^ ratio, mean (SD) | 1.24 (0.66) | 1.26 (0.75)^a,b,c^ | 1.28 (0.67)^a^ | 1.18 (0.52) | 1.25 (0.69)^a^ |
| Renal replacement therapy, n (%) | 641 (2) | 153 (1)^b^ | 117 (1)^b^ | 125 (1) | 246 (4)^a^ |
| Highest Anion Gap, median (IQR), mmol/L | 14 (12, 17) | 14 (11, 16)^b,c^ | 15 (12, 18)^a,b^ | 14 (11, 16) | 15 (12, 17)^a^ |
| Arterial Blood Gas tested, n (%) | 6,115 (15) | 1,717 (14)^c^ | 1,992 (20)^a,b^ | 1,629 (13) | 777 (13) |
| pH < 7.3 among tested, n (%) | 1,437 (23) | 477 (28)^a,b^ | 561 (28)^a,b^ | 281 (17) | 118 (15) |
| Highest Base deficit, mean (SD), mmol/L | 4.8 (4.7) | 4.8 (4.7)^a,c^ | 6.0 (5.4)^a,b^ | 3.6 (3.4) | 4.1 (3.7) |
| Lactate, tested, n (%) | 15,447 (37) | 4,194 (33)^b,c^ | 4,725 (47)^a,b^ | 4,139 (32) | 2,389 (39)^a^ |
| 2 - 4 mmol/L among tested, n (%) | 3,739 (24) | 992 (24)^a,c^ | 1,384 (29)^a,b^ | 847 (20) | 516 (22) |
| > 4 mmol/L among tested, n (%) | 1,374 (9) | 355 (8)^a,b,c^ | 625 (13)^a,b^ | 246 (6) | 148 (6) |
| **Inflammation** |  |  |  |  |  |
| Highest White blood cell count, median (IQR), x10^9/L | 9 (7, 13) | 9 (7, 12)^a,b,c^ | 11 (8, 14)^a,b^ | 9 (7, 12) | 9 (7, 12) |
| Highest Premature neutrophils (bands)), median (IQR), % | 10 (4, 20) | 9 (3, 18)^a,c^ | 12 (5, 24)^a,b^ | 7 (3, 15) | 7 (3, 14) |
| Lowest Lymphocytes, median (IQR), % | 16 (9, 24) | 17 (9, 26)^a,c^ | 12 (6, 20)^a,b^ | 17 (10, 26) | 16 (10, 24)^a^ |
| C-reactive protein, tested, n (%) | 5,862 (14) | 1,608 (13)^b,c^ | 1,741 (17)^a,b^ | 1,562 (12) | 951 (16)^a^ |
| Highest C-reactive protein, median (IQR), mg/L | 18 (5, 77) | 17 (4, 70)^a,c^ | 51 (10, 120)^a,b^ | 10 (3, 50) | 13 (4, 58) |
| Erythrocyte sedimentation rate, tested, n (%) | 3,903 (9) | 1,063 (8)^b,c^ | 1,055 (11)^a^ | 1,113 (9) | 672 (11)^a^ |
| Highest Erythrocyte sedimentation rate, median (IQR), mm/h | 40 (19, 73) | 36 (17, 67)^b,c^ | 48 (23, 84)^a^ | 33 (16, 64) | 47 (22, 76)^a^ |
| Highest Temperature, mean (SD), celsius | 37.7 (0.6) | 37.7 (0.6)^a,c^ | 37.9 (0.7)^a,b^ | 37.6 (0.5) | 37.7 (0.6) |
| 38 - 39, n (%) | 8,633 (21) | 2,617 (21)^a,b,c^ | 2,531 (25)^a,b^ | 2,336 (18) | 1,149 (19) |
| > 39, n (%) | 1,548 (4) | 339 (3)^a,c^ | 816 (8)^a,b^ | 251 (2) | 142 (2) |
| Lowest Temperature, mean (SD), celsius | 36.7 (1.0) | 36.6 (1.2)^a,b,c^ | 36.7 (0.9)^a^ | 36.7 (0.9) | 36.8 (0.7)^a^ |
| **Hematologic** |  |  |  |  |  |
| Lowest Hemoglobin, mean (SD), g/dL | 11.5 (2.3) | 11.3 (2.3)^a,b^ | 11.2 (2.4)^a,b^ | 11.9 (2.2) | 11.9 (2.3) |
| Highest RDW, mean (SD), % | 15.5 (2.1) | 15.5 (2.3)^a,b,c^ | 15.8 (2.3)^a,b^ | 15.2 (1.9) | 15.5 (2.0)^a^ |
| Lowest Platelets, median (IQR), x10^9/L | 210 (161, 269) | 204 (154, 264)^a,b,c^ | 221 (164, 288)^a,b^ | 208 (163, 260) | 214 (167, 270)^a^ |
| Platelets < 200, n (%) | 16,707 (40) | 5,349 (43)^b,c^ | 3,823 (38)^a,b^ | 5,153 (40) | 2,382 (39)^a^ |
| < 100 | 2,643 (16) | 951 (18)^a,b,c^ | 811 (21)^a,b^ | 615 (12) | 266 (11) |
| 100 - 200 | 14,064 (84) | 4,398 (82)^a,b,c^ | 3,012 (79)^a,b^ | 4,538 (88) | 2,116 (89) |
| International normalized ratio, tested, n (%) | 20,357 (49) | 5,586 (45)^a,b,c^ | 5,152 (51)^a,b^ | 6,077 (47) | 3,542 (58)^a^ |
| >= 2 | 1,836 (9) | 645 (12)^a,b,c^ | 509 (10)^a,b^ | 475 (8) | 207 (6)^a^ |
| **Neurologic** |  |  |  |  |  |
| Glasgow Coma Scale score, n (%) |  |  |  |  |  |
| Moderate (9 - 12) | 1,708 (4) | 543 (4)^a,b^ | 491 (5)^a,b^ | 470 (4) | 204 (3) |
| Severe (<= 8) | 1,482 (4) | 396 (3)^c^ | 523 (5)^a,b^ | 364 (3) | 199 (3) |
| **Liver and metabolic** |  |  |  |  |  |
| Bilirubin, tested, n (%) | 21,183 (51) | 5,808 (46)^b,c^ | 5,921 (59)^a^ | 5,870 (46) | 3,584 (59)^a^ |
| >= 2 mg/dL, n (%) | 1,427 (7) | 564 (10)^a,b,c^ | 473 (8)^a,b^ | 282 (5) | 108 (3)^a^ |
| Highest Glucose, median (IQR), mg/dL | 126 (104, 170) | 119 (100, 158)^a,b,c^ | 130 (106, 177)^a,b^ | 125 (103, 167) | 137 (108, 198)^a^ |
| Albumin, tested, n (%) | 21,368 (51) | 5,873 (47)^b,c^ | 5,961 (60)^a^ | 5,929 (46) | 3,605 (59)^a^ |
| < 2.5 | 1,243 (6) | 407 (7)^a,b,c^ | 546 (9)^a,b^ | 191 (3) | 99 (3) |
| 2.5 - 3.5 | 6,904 (32) | 2,018 (34)^a,b,c^ | 2,227 (37)^a,b^ | 1,623 (27) | 1,036 (29) |

Abbreviation: ICU: intensive care unit; IMC: intermediate care unit; MAP: mean aterial pressure; RDW: red cell distribution width; SD: standard deviation; IQR: interquartile range.

All p-values were adjusted for multiple comparisons using Bonferroni method.

^a^ p < 0.05 compared to Physiotype C .

^b^ p < 0.05 compared to Physiotype D.

^c^ p < 0.05 compared to Physiotype B.

^d^ Cardiovascular disease was considered if there was a history of congestive heart failure, coronary artery disease of peripheral vascular disease.

^e^ Reference glomerular filtration rate and reference creatinine were derived without use of race correction (see S1 Text for details).
