# Supplementary material for: Physiologic signatures within six hours of hospitalization identify acute illness phenotypes
Source: PLOS Digit Health. 2022 Oct 13;1(10):e0000110. doi: 10.1371/journal.pdig.0000110 (PMC9802629; doi:10.1371/journal.pdig.0000110)
Supplement: S12 Table — (DOCX) [file pdig.0000110.s043.docx]

# S12 Table. Physiotype illness severity, clinical outcomes, and resource use in sensitivity analysis by excluding variables with high missingness (temperature) and correlation (diastolic blood pressure and respiratory rate) in the training cohort

| **Variables** | **Total** | **Acute Illness Physiotypes** | | | |
| --- | --- | --- | --- | --- | --- |
|  |  | Physiotype A | Physiotype B | Physiotype C | Physiotype D |
| Number of Encounters (%) | 41,502 | 12,544 (30) | 10,013 (24) | 12,874 (31) | 6,071 (15) |
| **Acuity scores within 24h of admission** |  |  |  |  |  |
| SOFA score > 6, n (%) | 3,506 (8) | 1,293 (10)^a,b^ | 1,021 (10)^a,b^ | 838 (7) | 354 (6) |
| Patients in ICU/IMC, SOFA score <= 6, n (%) | 6,882 (17) | 1,756 (14)^b,c^ | 2,144 (21)^a,b^ | 1,870 (15) | 1,112 (18)^a^ |
| Patients in ICU/IMC, SOFA score > 6, n (%) | 2,544 (6) | 888 (7)^a,b,c^ | 862 (9)^a,b^ | 545 (4) | 249 (4) |
| Patients in ward, SOFA score <= 6, n (%) | 31,114 (75) | 9,495 (76)^a,c^ | 6,848 (68)^a,b^ | 10,166 (79) | 4,605 (76)^a^ |
| Patients in ward, SOFA score > 6, n (%) | 962 (2) | 405 (3)^a,b,c^ | 159 (2)^a^ | 293 (2) | 105 (2) |
| MEWS score > 4, n (%) | 2,828 (7) | 498 (4)^a,b,c^ | 1,582 (16)^a,b^ | 267 (2) | 481 (8)^a^ |
| Patients in ICU/IMC, MEWS score <= 4, n (%) | 7,235 (17) | 2,235 (18) | 1,760 (18) | 2,195 (17) | 1,045 (17) |
| Patients in ICU/IMC, MEWS score > 4, n (%) | 2,191 (5) | 409 (3)^a,b,c^ | 1,246 (12)^a,b^ | 220 (2) | 316 (5)^a^ |
| Patients in ward, MEWS score <= 4, n (%) | 31,439 (76) | 9,811 (78)^a,b,c^ | 6,671 (67)^a,b^ | 10,412 (81) | 4,545 (75)^a^ |
| Patients in ward, MEWS score > 4, n (%) | 637 (2) | 89 (1)^a,b,c^ | 336 (3)^a^ | 47 (0) | 165 (3)^a^ |
| **Resource use during hospitalization** |  |  |  |  |  |
| Hospital days, median (IQR) | 4 (2, 7) | 4 (2, 6)^a,c^ | 4 (3, 8)^a,b^ | 3 (2, 6) | 4 (2, 7)^a^ |
| Surgery at any time, n (%) | 11,634 (28) | 4,266 (34)^b,c^ | 2,023 (20)^a,b^ | 4,261 (33) | 1,084 (18)^a^ |
| Admitted to ICU/IMC^d^, n (%) | 11,121 (27) | 3,118 (25)^a,b,c^ | 3,478 (35)^a,b^ | 2,876 (22) | 1,649 (27)^a^ |
| Days in ICU/IMC^e^, median (IQR) | 4 (2, 7) | 4 (3, 7)^a,b^ | 4 (3, 8)^a,b^ | 4 (2, 7) | 4 (2, 6) |
| Days in ICU/IMC greater than 48 hrs, n (%) | 8,332 (75) | 2,384 (76)^a^ | 2,684 (77)^a^ | 2,041 (71) | 1,223 (74) |
| Mechanical Ventilation, n (%) | 3,218 (8) | 999 (8)^a,b,c^ | 1,055 (11)^a,b^ | 811 (6) | 353 (6) |
| Mechanical Ventilation hours, median (IQR)^f^ | 35 (14, 113) | 26 (12, 87)^b,c^ | 45 (16, 139)^a^ | 25 (12, 105) | 52 (20, 141)^a^ |
| Mechanical Ventilation greater than 2 calendar days, n (%) | 1,661 (52) | 459 (46)^b,c^ | 614 (58)^a^ | 374 (46) | 214 (61)^a^ |
| Renal replacement therapy, n (%) | 1,262 (3) | 318 (3)^b^ | 287 (3)^a,b^ | 264 (2) | 393 (6)^a^ |
| **Complications** |  |  |  |  |  |
| Acute kidney injury overall, n (%) | 6,905 (17) | 1,989 (16)^a,b,c^ | 1,996 (20)^a^ | 1,721 (13) | 1,199 (20)^a^ |
| Community-acquired AKI, n (%) | 3,839 (56) | 1,244 (63)^a,b,c^ | 1,123 (56)^b^ | 905 (53) | 567 (47)^a^ |
| Hospital-acquired AKI, n (%) | 3,066 (44) | 745 (37)^a,b,c^ | 873 (44)^b^ | 816 (47) | 632 (53)^a^ |
| Worst AKI staging, n (%) |  |  |  |  |  |
| Stage 1 | 4,360 (63) | 1,173 (59)^a,b^ | 1,209 (61)^a,b^ | 1,184 (69) | 794 (66) |
| Stage 2 | 1,362 (20) | 422 (21)^a,b^ | 434 (22)^a,b^ | 299 (17) | 207 (17) |
| Stage 3 | 848 (12) | 285 (14)^a^ | 249 (12) | 177 (10) | 137 (11) |
| Stage 3 with RRT | 335 (5) | 109 (5)^a^ | 104 (5) | 61 (4) | 61 (5) |
| Venous Thromboembolism, n (%) | 1,257 (3) | 357 (3)^c^ | 407 (4)^a,b^ | 340 (3) | 153 (3) |
| Sepsis, n (%) | 3,750 (9) | 1,026 (8)^a,b,c^ | 1,825 (18)^a,b^ | 516 (4) | 383 (6)^a^ |
| Hospital disposition, n (%) |  |  |  |  |  |
| Hospital mortality | 1,141 (3) | 315 (3)^a,c^ | 455 (5)^a,b^ | 236 (2) | 135 (2) |
| Another hospital, LTAC, SNF, Hospice | 4,475 (11) | 1,219 (10)^b,c^ | 1,224 (12)^a^ | 1,279 (10) | 753 (12)^a^ |
| Home or short-term rehabilitation | 35,886 (86) | 11,010 (88)^b,c^ | 8,334 (83)^a,b^ | 11,359 (88) | 5,183 (85)^a^ |
| Thirty-day mortality, n (%) | 1,633 (4) | 455 (4)^a,c^ | 623 (6)^a,b^ | 352 (3) | 203 (3) |
| Three-year mortality, n (%) | 8,013 (19) | 2,224 (18)^b,c^ | 2,333 (23)^a^ | 2,130 (17) | 1,326 (22)^a^ |

Abbreviation: SOFA: sequential organ failure assessment; MEWS: modified early warning score; ICU: intensive care unit; IMC: intermediate care unit; IQR: interquartile range.

All p-values were adjusted for multiple comparisons using Bonferroni method.

^a^ p < 0.05 compared to Physiotype C .

^b^ p < 0.05 compared to Physiotype D.

^c^ p < 0.05 compared to Physiotype B.

^d^ At any time during hospitalization.

^e^ Values were calculated among patients admitted to ICU/IMC.

^f^ Values were calculated among patients requiring MV.
