# Supplementary material for: Physiologic signatures within six hours of hospitalization identify acute illness phenotypes
Source: PLOS Digit Health. 2022 Oct 13;1(10):e0000110. doi: 10.1371/journal.pdig.0000110 (PMC9802629; doi:10.1371/journal.pdig.0000110)
Supplement: S13 Table — (DOCX) [file pdig.0000110.s044.docx]

# S13 Table. Physiotype clinical characteristics and biomarkers in sensitivity analysis by using a 12 hour window of EHR data in the training cohort

| **Variables** | **Total** | **Acute Illness Physiotypes** | | | |
| --- | --- | --- | --- | --- | --- |
|  |  | Physiotype A | Physiotype B | Physiotype C | Physiotype D |
| Number of Encounters (%) | 41,502 | 12,229 (29) | 8,694 (21) | 13,563 (33) | 7,016 (17) |
| **Preadmission clinical characteristics** |  |  |  |  |  |
| Age, mean (SD) | 54 (19) | 52 (19)^a,b,c^ | 50 (19)^a,b^ | 57 (18) | 57 (17) |
| Female sex, n (%) | 22,745 (55) | 7,319 (60)^a,b,x^ | 5,004 (58)^a,b^ | 6,849 (50) | 3,573 (51) |
| Race, n (%) |  |  |  |  |  |
| White | 29,076 (70) | 9,235 (76)^a,b,c^ | 5,995 (69)^a,b^ | 9,730 (72) | 4,116 (59)^a^ |
| African American | 9,634 (23) | 1,977 (16)^a,b,c^ | 2,135 (25)^a,b^ | 2,936 (22) | 2,586 (37)^a^ |
| Primary Insurance, n (%) |  |  |  |  |  |
| Private | 9,591 (23) | 3,113 (25)^a,b^ | 2,081 (24)^b^ | 3,115 (23) | 1,282 (18)^a^ |
| Medicare | 18,499 (45) | 5,159 (42)^a,b,c^ | 3,401 (39)^a,b^ | 6,520 (48) | 3,419 (49) |
| Medicaid | 9,231 (22) | 2,844 (23)^a,b,c^ | 2,295 (26)^a,b^ | 2,620 (19) | 1,472 (21)^a^ |
| Uninsured | 4,181 (10) | 1,113 (9)^b,c^ | 917 (11)^b^ | 1,308 (10) | 843 (12)^a^ |
| Residency area characteristics |  |  |  |  |  |
| Total Proportion of African-American (%), mean (SD) | 18.7 (17.5) | 17.3 (16.1)^a,b,c^ | 19.5 (18.0)^a,b^ | 18.3 (17.1) | 21.3 (19.4)^a^ |
| Population Proportion Below Poverty (%), mean (SD) | 22.7 (10.1) | 21.9 (9.8)^a,b,c^ | 23.2 (10.0)^a,b^ | 22.4 (10.1) | 24.1 (10.3)^a^ |
| distance from Residency to Hospital (mile), median (IQR) | 18 (3, 34) | 22 (3, 37)^a,b,c^ | 14 (3, 32)^a,b^ | 18 (3, 36) | 14 (3, 27)^a^ |
| **Comorbidities** |  |  |  |  |  |
| Hypertension, n (%) | 21,639 (52) | 6,205 (51)^b^ | 4,472 (51)^b^ | 7,054 (52) | 3,908 (56)^a^ |
| Cardiovascular disease, n (%)^d^ | 12,058 (29) | 3,363 (28)^b^ | 2,515 (29)^b^ | 3,920 (29) | 2,260 (32)^a^ |
| Diabetes mellitus, n (%) | 10,111 (24) | 2,836 (23)^b^ | 2,134 (25)^b^ | 3,230 (24) | 1,911 (27)^a^ |
| Chronic kidney disease, n (%) | 6,518 (16) | 1,636 (13)^a,b,c^ | 1,287 (15)^b^ | 2,115 (16) | 1,480 (21)^a^ |
| **Admission characteristics of patients** |  |  |  |  |  |
| Emergent Admission, n (%) | 30,177 (73) | 7,480 (61)^a,b,c^ | 7,300 (84)^a,b^ | 9,182 (68) | 6,215 (89)^a^ |
| Transfer from another hospital, n (%) | 7,115 (17) | 1,989 (16)^c^ | 1,807 (21)^a,b^ | 2,099 (15) | 1,220 (17)^a^ |
| **Primary admission diagnostic groups** |  |  |  |  |  |
| Diseases of the circulatory system, n (%) | 7,719 (19) | 2,047 (17)^a,b,c^ | 1,313 (15)^a,b^ | 2,644 (19) | 1,715 (24)^a^ |
| Respiratory and infectious diseases, n (%) | 3,306 (8) | 593 (5)^b,c^ | 1,302 (15)^a,b^ | 704 (5) | 707 (10)^a^ |
| Complications of pregnancy and childbirth, n (%) | 3,148 (8) | 1,056 (9)^a,b,c^ | 926 (11)^a,b^ | 826 (6) | 340 (5)^a^ |
| Diseases of the digestive/genitourinary systems, n (%) | 5,184 (12) | 1,746 (14)^b,c^ | 866 (10)^a^ | 1,810 (13) | 762 (11)^a^ |
| Diseases of the musculoskeletal/connective tissue and skin, n (%) | 3,651 (9) | 1,266 (10)^b,c^ | 446 (5)^a,b^ | 1,396 (10) | 543 (8)^a^ |
| Neoplasms, n (%) | 2,743 (7) | 1,093 (9)^b,c^ | 335 (4)^a,b^ | 1,101 (8) | 214 (3)^a^ |
| **Clinical biomarkers and interventions within 24 hours of admission** |  |  |  |  |  |
| Surgery on admission day, n (%) | 8,644 (21) | 3,838 (31)^a,b,c^ | 706 (8)^a^ | 3,550 (26) | 550 (8)^a^ |
| ICU/IMC admission within first 24 hours, n (%) | 9,426 (23) | 3,008 (25)^a,b,c^ | 2,863 (33)^a,b^ | 2,224 (16) | 1,331 (19)^a^ |
| **Cardiovascular system** |  |  |  |  |  |
| Hypotension (MAP < 60 mmHg) at any time, n (%) | 14,470 (35) | 7,311 (60)^a,b,c^ | 2,927 (34)^a,b^ | 3,659 (27) | 573 (8)^a^ |
| Duration, median (IQR), minutes | 57 (15, 168) | 83 (27, 240)^a,b,c^ | 69 (28, 195)^a,b^ | 15 (5, 54) | 15 (5, 55) |
| Vasopressors used, n (%) | 7,531 (18) | 3,632 (30)^a,b,c^ | 887 (10)^a,b^ | 2,618 (19) | 394 (6)^a^ |
| Out of operating room | 1,403 (3) | 690 (6)^a,b^ | 448 (5)^a,b^ | 208 (2) | 57 (1)^a^ |
| Hypertension (SBP > 160 mmHg) at any time, n (%) | 14,838 (36) | 2,166 (18)^a,b^ | 1,488 (17)^a,b^ | 5,662 (42) | 5,522 (79)^a^ |
| Troponin, tested, n (%) | 14,616 (35) | 3,167 (26)^a,b,c^ | 3,664 (42)^a,b^ | 4,256 (31) | 3,529 (50)^a^ |
| Abnormal result among tested, n (%) | 3,398 (23) | 849 (27)^a,c^ | 871 (24)^a^ | 814 (19) | 864 (24)^a^ |
| **Respiratory system** |  |  |  |  |  |
| Highest administered FiO2, median (IQR) | 0.21 (0.21, 0.40) | 0.21 (0.21, 0.40)^a,b,c^ | 0.21 (0.21, 0.33)^b^ | 0.21 (0.21, 0.40) | 0.21 (0.21, 0.29)^a^ |
| Room air only, n (%) | 23,963 (58) | 6,377 (52)^a,b,c^ | 4,869 (56)^a,b^ | 7,952 (59) | 4,765 (68)^a^ |
| 0.22 - 0.40, n (%) | 14,790 (36) | 4,863 (40)^a,b,c^ | 3,012 (35)^a,b^ | 4,950 (36) | 1,965 (28)^a^ |
| > 0.4, n (%) | 2,749 (7) | 989 (8)^a,b,c^ | 813 (9)^a,b^ | 661 (5) | 286 (4) |
| PaO2/FiO2, tested with arterial blood gas, n (%) | 6,113 (15) | 2,003 (16)^a,b,c^ | 1,848 (21)_a,b_ | 1,456 (11) | 806 (11) |
| <200 among tested, n (%) | 2,265 (37) | 778 (39)^a,b^ | 793 (43)^a,b^ | 439 (30) | 255 (32) |
| Mechanical ventilation, n (%) | 2,123 (5) | 870 (7)^a,b^ | 624 (7)^a,b^ | 436 (3) | 193 (3) |
| **Kidney and acid-base status** |  |  |  |  |  |
| Preadmission estimated glomerular filtration rate^e^ (mL/min per 1.73 m2), median (IQR) | 95 (78, 111) | 97 (81, 113)^a,b,c^ | 100 (83, 117)^a,b^ | 92 (77, 107) | 90 (59, 105)^a^ |
| Highest / reference creatinine^e^ ratio, mean (SD) | 1.24 (0.66) | 1.26 (0.74)^a,c^ | 1.30 (0.7)^a,b^ | 1.18 (0.54) | 1.23 (0.65)^a^ |
| Renal replacement therapy, n (%) | 641 (2) | 173 (1)^a,b^ | 103 (1)^b^ | 131 (1) | 234 (3)^a^ |
| Highest Anion Gap, median (IQR), mmol/L | 14 (12, 17) | 14 (11, 16)^b,c^ | 15 (12, 18)^a,b^ | 14 (11, 16) | 15 (12, 17)^a^ |
| Arterial Blood Gas tested, n (%) | 6,115 (15) | 2,005 (16)^a,b,c^ | 1,848 (21)^a,b^ | 1,456 (11) | 806 (11) |
| pH < 7.3 among tested, n (%) | 1,437 (23) | 560 (28)^a,b^ | 533 (29)^a,b^ | 235 (16) | 109 (14) |
| Highest Base deficit, mean (SD), mmol/L | 4.8 (4.7) | 4.7 (4.4)^b,c^ | 6.4 (5.8)^a,b^ | 3.4 (3.0) | 3.9 (3.3) |
| Lactate, tested, n (%) | 15,447 (37) | 4,369 (36)^a,b,c^ | 4,285 (49)^a,b^ | 4,092 (30) | 2,701 (38)^a^ |
| 2 - 4 mmol/L among tested, n (%) | 3,739 (24) | 1,038 (24)^a,c^ | 1,243 (29)^a,b^ | 847 (21) | 611 (23) |
| > 4 mmol/L among tested, n (%) | 1,374 (9) | 436 (10)^a,b,c^ | 564 (13)^a,b^ | 196 (5) | 178 (7)^a^ |
| **Inflammation** |  |  |  |  |  |
| Highest White blood cell count, median (IQR), x10^9/L | 9 (7, 13) | 10 (7, 13)^a,b,c^ | 11 (8, 15)^a,b^ | 9 (7, 12) | 9 (7, 12) |
| Highest Premature neutrophils (bands)), median (IQR), % | 10 (4, 20) | 10 (4, 19)^a,b,c^ | 12 (5, 24)^a,b^ | 6 (3, 14) | 7 (3, 15) |
| Lowest Lymphocytes, median (IQR), % | 16 (9, 24) | 16 (8, 25)^a,b,c^ | 11 (6, 19)^a,b^ | 18 (11, 26) | 17 (10, 25)^a^ |
| C-reactive protein, tested, n (%) | 5,862 (14) | 1,499 (12)^b,c^ | 1,572 (18)^a,b^ | 1,691 (12) | 1,100 (16)^a^ |
| Highest C-reactive protein, median (IQR), mg/L | 18 (5, 77) | 18 (5, 75)^a,b,c^ | 57 (12, 131)^a,b^ | 12 (3, 51) | 11 (4, 51) |
| Erythrocyte sedimentation rate, tested, n (%) | 3,903 (9) | 958 (8)^a,b,c^ | 946 (11)^a^ | 1,196 (9) | 803 (11)^a^ |
| Highest Erythrocyte sedimentation rate, median (IQR), mm/h | 40 (19, 73) | 37 (17, 67)^c^ | 52 (26, 88)^a,b^ | 34 (16, 64) | 39 (19, 73)^a^ |
| Highest Temperature, mean (SD), celsius | 37.7 (0.6) | 37.7 (0.6)^a,b,c^ | 37.9 (0.8)^a,b^ | 37.6 (0.5) | 37.6 (0.5) |
| 38 - 39, n (%) | 8,633 (21) | 2,699 (22)^a,b,c^ | 2,216 (25)^a,b^ | 2,446 (18) | 1,272 (18) |
| > 39, n (%) | 1,548 (4) | 352 (3)^a,c^ | 826 (10)^a,b^ | 212 (2) | 158 (2)^a^ |
| Lowest Temperature, mean (SD), celsius | 36.7 (1.0) | 36.5 (1.4)^a,b,c^ | 36.8 (0.8)^a,b^ | 36.7 (0.8) | 36.8 (0.6)^a^ |
| **Hematologic** |  |  |  |  |  |
| Lowest Hemoglobin, mean (SD), g/dL | 11.5 (2.3) | 11.0 (2.3)^a,b,c^ | 11.2 (2.4)^a,b^ | 12.0 (2.2) | 12.1 (2.3)^a^ |
| Highest RDW, mean (SD), % | 15.5 (2.1) | 15.5 (2.3)^a,b,c^ | 15.9 (2.3)^a,b^ | 15.2 (1.9) | 15.5 (2.0)^a^ |
| Lowest Platelets, median (IQR), x10^9/L | 210 (161, 269) | 201 (152, 260)^a,b,c^ | 218 (162, 287)^a^ | 210 (165, 263) | 218 (170, 275)^a^ |
| Platelets < 200, n (%) | 16,707 (40) | 5,377 (44)^b,c^ | 3,440 (40)^a,b^ | 5,220 (38) | 2,670 (38)^a^ |
| < 100 | 2,643 (16) | 986 (18) | 735 (21) | 616 (12) | 306 (11) |
| 100 - 200 | 14,064 (84) | 4,391 (82) | 2,705 (79) | 4,604 (88) | 2,364 (89) |
| International normalized ratio, tested, n (%) | 20,357 (49) | 5,571 (46)^b,c^ | 4,693 (54)^a^ | 6,243 (46) | 3,850 (55)^a^ |
| >= 2 | 1,836 (9) | 610 (11)^a,b^ | 523 (11)^a,b^ | 473 (8) | 230 (6)^a^ |
| **Neurologic** |  |  |  |  |  |
| Glasgow Coma Scale score, n (%) |  |  |  |  |  |
| Moderate (9 - 12) | 1,708 (4) | 644 (5)^a,b^ | 437 (5)^a,b^ | 418 (3) | 209 (3) |
| Severe (<= 8) | 1,482 (4) | 505 (4)^a,b,c^ | 470 (5)^a,b^ | 325 (2) | 182 (3) |
| **Liver and metabolic** |  |  |  |  |  |
| Bilirubin, tested, n (%) | 21,183 (51) | 5,537 (45)^b,c^ | 5,352 (62)^a^ | 6,104 (45) | 4,190 (60)^a^ |
| >= 2 mg/dL, n (%) | 1,427 (7) | 548 (10)^a,b,c^ | 438 (8)^a,b^ | 314 (5) | 127 (3)^a^ |
| Highest Glucose, median (IQR), mg/dL | 126 (104, 170) | 123 (101, 164)^a,b,c^ | 130 (106, 176)^a^ | 125 (103, 166) | 130 (106, 184)^a^ |
| Albumin, tested, n (%) | 21,368 (51) | 5,611 (46)^b,c^ | 5,377 (62)^a^ | 6,164 (45) | 4,216 (60)^a^ |
| < 2.5 | 1,243 (6) | 453 (8)^a,b^ | 505 (9)^a,b^ | 176 (3) | 109 (3) |
| 2.5 - 3.5 | 6,904 (32) | 1,936 (35)^a,b,c^ | 2,139 (40)^a,b^ | 1,645 (27) | 1,184 (28) |

Abbreviation: ICU: intensive care unit; IMC: intermediate care unit; MAP: mean aterial pressure; RDW: red cell distribution width; SD: standard deviation; IQR: interquartile range.

All p-values were adjusted for multiple comparisons using Bonferroni method.

^a^ p < 0.05 compared to Physiotype C .

^b^ p < 0.05 compared to Physiotype D.

^c^ p < 0.05 compared to Physiotype B.

^d^ Cardiovascular disease was considered if there was a history of congestive heart failure, coronary artery disease of peripheral vascular disease.

^e^ Reference glomerular filtration rate and reference creatinine were derived without use of race correction (see S1 Text for details).
