# Supplementary material for: Physiologic signatures within six hours of hospitalization identify acute illness phenotypes
Source: PLOS Digit Health. 2022 Oct 13;1(10):e0000110. doi: 10.1371/journal.pdig.0000110 (PMC9802629; doi:10.1371/journal.pdig.0000110)
Supplement: S14 Table — (DOCX) [file pdig.0000110.s045.docx]

# S14 Table. Physiotype illness severity, clinical outcomes, and resource use in sensitivity analysis by using a 12 hour window of EHR data in the training cohort

| **Variables** | **Total** | **Acute Illness Physiotypes** | | | |
| --- | --- | --- | --- | --- | --- |
|  |  | Physiotype A | Physiotype B | Physiotype C | Physiotype D |
| Number of Encounters (%) | 41,502 | 12,229 (29) | 8,694 (21) | 13,563 (33) | 7,016 (17) |
| **Acuity scores within 24h of admission** |  |  |  |  |  |
| SOFA score > 6, n (%) | 3,506 (8) | 1,554 (13)^a,b,c^ | 890 (10)^a,b^ | 760 (6) | 302 (4)^a^ |
| Patients in ICU/IMC, SOFA score <= 6, n (%) | 6,882 (17) | 1,905 (16)^a,c^ | 2,092 (24)^a,b^ | 1,767 (13) | 1,118 (16)^a^ |
| Patients in ICU/IMC, SOFA score > 6, n (%) | 2,544 (6) | 1,103 (9)^a,b^ | 771 (9)^a,b^ | 457 (3) | 213 (3) |
| Patients in ward, SOFA score <= 6, n (%) | 31,114 (75) | 8,770 (72)^a,b,c^ | 5,712 (66)^a,b^ | 11,036 (81) | 5,596 (80)^a^ |
| Patients in ward, SOFA score > 6, n (%) | 962 (2) | 451 (4)^a,b,c^ | 119 (1)^a^ | 303 (2) | 89 (1)^a^ |
| MEWS score > 4, n (%) | 2,828 (7) | 513 (4)^a,b,c^ | 1,544 (18)^a,b^ | 246 (2) | 525 (7)^a^ |
| Patients in ICU/IMC, MEWS score <= 4, n (%) | 7,235 (17) | 2,578 (21)^a,b,c^ | 1,622 (19)^a,b^ | 2,031 (15) | 1,004 (14) |
| Patients in ICU/IMC, MEWS score > 4, n (%) | 2,191 (5) | 430 (4)^a,b,c^ | 1,241 (14)^a,b^ | 193 (1) | 327 (5)^a^ |
| Patients in ward, MEWS score <= 4, n (%) | 31,439 (76) | 9,138 (75)^a,b,c^ | 5,528 (64)^a,b^ | 11,286 (83) | 5,487 (78)^a^ |
| Patients in ward, MEWS score > 4, n (%) | 637 (2) | 83 (1)^a,b,c^ | 303 (3)^a^ | 53 (0) | 198 (3)^a^ |
| **Resource use during hospitalization** |  |  |  |  |  |
| Hospital days, median (IQR) | 4 (2, 7) | 4 (2, 7)^a,b,c^ | 5 (3, 8)^a,b^ | 3 (2, 6) | 4 (2, 6)^a^ |
| Surgery at any time, n (%) | 11,634 (28) | 4,621 (38)^a,b,c^ | 1,394 (16)^a^ | 4,530 (33) | 1,089 (16)^a^ |
| Admitted to ICU/IMC^d^, n (%) | 11,121 (27) | 3,435 (28)^a,b,c^ | 3,317 (38)^a,b^ | 2,699 (20) | 1,670 (24)^a^ |
| Days in ICU/IMC^e^, median (IQR) | 4 (2, 7) | 4 (3, 8)^a,b^ | 4 (3, 8)^a,b^ | 4 (2, 7) | 4 (2, 6) |
| Days in ICU/IMC greater than 48 hrs, n (%) | 8,332 (75) | 2,639 (77)^a,b^ | 2,575 (78)^a,b^ | 1,899 (70) | 1,219 (73) |
| Mechanical Ventilation, n (%) | 3,218 (8) | 1,209 (10)^a,b,c^ | 972 (11)^a,b^ | 721 (5) | 316 (5) |
| Mechanical Ventilation hours, median (IQR)^f^ | 35 (14, 113) | 25 (11, 81)^b,c^ | 52 (19, 148)^a^ | 27 (12, 103) | 50 (18, 134)^a^ |
| Mechanical Ventilation greater than 2 calendar days, n (%) | 1,661 (52) | 542 (45)^b,c^ | 594 (61)^a^ | 337 (47) | 188 (59)^a^ |
| Renal replacement therapy, n (%) | 1,262 (3) | 349 (3)^a,b^ | 270 (3)^a,b^ | 276 (2) | 367 (5)^a^ |
| **Complications** |  |  |  |  |  |
| Acute kidney injury overall, n (%) | 6,905 (17) | 1,988 (16)^a,c^ | 1,948 (22)^a,b^ | 1,734 (13) | 1,235 (18)^a^ |
| Community-acquired AKI, n (%) | 3,839 (56) | 1,265 (64)^a,b,c^ | 1,088 (56)^b^ | 916 (53) | 570 (46)^a^ |
| Hospital-acquired AKI, n (%) | 3,066 (44) | 723 (36)^a,b,c^ | 860 (44)^b^ | 818 (47) | 665 (54)^a^ |
| Worst AKI staging, n (%) |  |  |  |  |  |
| Stage 1 | 4,360 (63) | 1,177 (59)^a,b^ | 1,151 (59)^a,b^ | 1,216 (70) | 816 (66) |
| Stage 2 | 1,362 (20) | 423 (21)^a,b^ | 431 (22)^a,b^ | 295 (17) | 213 (17) |
| Stage 3 | 848 (12) | 273 (14)^a^ | 257 (13)^a^ | 164 (9) | 154 (12) |
| Stage 3 with RRT | 335 (5) | 115 (6)^a^ | 109 (6)^a^ | 59 (3) | 52 (4) |
| Venous Thromboembolism, n (%) | 1,257 (3) | 349 (3)^c^ | 365 (4)^a,b^ | 346 (3) | 197 (3) |
| Sepsis, n (%) | 3,750 (9) | 1,041 (9)^a,b,c^ | 1,814 (21)^a,b^ | 476 (4) | 419 (6)^a^ |
| Hospital disposition, n (%) |  |  |  |  |  |
| Hospital mortality | 1,141 (3) | 349 (3)^a,b,c^ | 465 (5)^a,b^ | 195 (1) | 132 (2) |
| Another hospital, LTAC, SNF, Hospice | 4,475 (11) | 1,250 (10)^c^ | 1,116 (13)^a,b^ | 1,312 (10) | 797 (11)^a^ |
| Home or short-term rehabilitation | 35,886 (86) | 10,630 (87)^a,c^ | 7,113 (82)^a,b^ | 12,056 (89) | 6,087 (87)^a^ |
| Thirty-day mortality, n (%) | 1,633 (4) | 477 (4)^a,b,c^ | 632 (7)^a,b^ | 317 (2) | 207 (3) |
| Three-year mortality, n (%) | 8,013 (19) | 2,223 (18)^a,b,c^ | 2,231 (26)^a,b^ | 2,139 (16) | 1,420 (20)^a^ |

Abbreviation: SOFA: sequential organ failure assessment; MEWS: modified early warning score; ICU: intensive care unit; IMC: intermediate care unit; IQR: interquartile range.

All p-values were adjusted for multiple comparisons using Bonferroni method.

^a^ p < 0.05 compared to Physiotype C .

^b^ p < 0.05 compared to Physiotype D.

^c^ p < 0.05 compared to Physiotype B.

^d^ At any time during hospitalization.

^e^ Values were calculated among patients admitted to ICU/IMC.

^f^ Values were calculated among patients requiring MV.
