# Supplementary material for: Physiologic signatures within six hours of hospitalization identify acute illness phenotypes
Source: PLOS Digit Health. 2022 Oct 13;1(10):e0000110. doi: 10.1371/journal.pdig.0000110 (PMC9802629; doi:10.1371/journal.pdig.0000110)
Supplement: S15 Table — (DOCX) [file pdig.0000110.s046.docx]

# S15 Table. Centroids of physiotypes for prediction

| **Hours from hospital admission** | **Acute Illness Physiotypes** | | | |
| --- | --- | --- | --- | --- |
|  | Physiotype A | Physiotype B | Physiotype C | Physiotype D |
| **Systolic blood pressure (mmHg)** |  |  |  |  |
| [0, 1) | 116.92 | 121.83 | 136.54 | 159.22 |
| [1, 2) | 113.98 | 119.81 | 136.13 | 159.93 |
| [2, 3) | 110.59 | 118.56 | 134.67 | 158.94 |
| [3, 4) | 109.47 | 117.99 | 133.39 | 157.57 |
| [4, 5) | 109.47 | 117.56 | 132.97 | 155.89 |
| [5, 6) | 110.75 | 117.63 | 132.85 | 153.65 |
| **Diastolic blood pressure (mmHg)** |  |  |  |  |
| [0, 1) | 65.61 | 71.47 | 75.94 | 89.85 |
| [1, 2) | 63.64 | 69.68 | 75.31 | 89.89 |
| [2, 3) | 61.69 | 68.56 | 74.46 | 89.07 |
| [3, 4) | 60.82 | 67.94 | 73.62 | 88.00 |
| [4, 5) | 60.75 | 67.59 | 73.29 | 87.09 |
| [5, 6) | 61.22 | 67.57 | 73.03 | 85.80 |
| **Heart rate (beats per minute)** |  |  |  |  |
| [0, 1) | 78.25 | 100.48 | 77.88 | 88.48 |
| [1, 2) | 76.50 | 99.95 | 76.24 | 87.13 |
| [2, 3) | 75.07 | 99.09 | 75.51 | 86.58 |
| [3, 4) | 74.26 | 98.27 | 74.91 | 86.26 |
| [4, 5) | 74.42 | 97.11 | 74.65 | 85.92 |
| [5, 6) | 74.96 | 96.02 | 74.69 | 85.35 |
| **Temperature (degree Celsius)** |  |  |  |  |
| [0, 1) | 37.23 | 37.34 | 37.22 | 37.23 |
| [1, 2) | 37.25 | 37.36 | 37.23 | 37.24 |
| [2, 3) | 37.23 | 37.36 | 37.23 | 37.24 |
| [3, 4) | 37.23 | 37.36 | 37.22 | 37.24 |
| [4, 5) | 37.20 | 37.35 | 37.22 | 37.25 |
| [5, 6) | 37.17 | 37.34 | 37.21 | 37.24 |
| **Peripheral capillary oxygen saturation (%)** |  |  |  |  |
| [0, 1) | 97.19 | 96.59 | 97.17 | 96.91 |
| [1, 2) | 97.31 | 96.61 | 97.17 | 96.80 |
| [2, 3) | 97.45 | 96.54 | 97.20 | 96.72 |
| [3, 4) | 97.48 | 96.54 | 97.14 | 96.70 |
| [4, 5) | 97.41 | 96.54 | 97.08 | 96.65 |
| [5, 6) | 97.34 | 96.56 | 97.03 | 96.64 |
| **Respiratory rate (breaths per minute)** |  |  |  |  |
| [0, 1) | 16.58 | 20.11 | 16.86 | 18.56 |
| [1, 2) | 16.06 | 20.48 | 16.70 | 18.54 |
| [2, 3) | 14.99 | 20.45 | 16.22 | 18.52 |
| [3, 4) | 14.60 | 20.32 | 16.07 | 18.40 |
| [4, 5) | 14.64 | 20.08 | 16.09 | 18.36 |
| [5, 6) | 15.02 | 19.82 | 16.18 | 18.27 |
