# Supplementary material for: Physiologic signatures within six hours of hospitalization identify acute illness phenotypes
Source: PLOS Digit Health. 2022 Oct 13;1(10):e0000110. doi: 10.1371/journal.pdig.0000110 (PMC9802629; doi:10.1371/journal.pdig.0000110)
Supplement: S16 Table — (DOCX) [file pdig.0000110.s047.docx]

# S16 Table. Physiotype clinical characteristics and biomarkers in the testing cohort

| **Variables** | **Total** | **Acute Illness Physiotypes** | | | |
| --- | --- | --- | --- | --- | --- |
|  |  | Physiotype A | Physiotype B | Physiotype C | Physiotype D |
| Number of Encounters (%) | 16,845 | 4,970 (30) | 4,016 (24) | 5,288 (31) | 2,571 (15) |
| **Preadmission clinical characteristics** |  |  |  |  |  |
| Age, mean (SD) | 55 (19) | 54 (19)^a,b,c^ | 51 (20)^a,b^ | 57 (18) | 57 (17) |
| Female sex, n (%) | 9,205 (55) | 2,855 (57)^a,b^ | 2,294 (57)^a,b^ | 2,707 (51) | 1,349 (52) |
| Race, n (%) |  |  |  |  |  |
| White | 11,854 (70) | 3,797 (76)^a,b,c^ | 2,777 (69)^b^ | 3,777 (71) | 1,503 (58)^a^ |
| African American | 3,845 (23) | 784 (16)^a,b,c^ | 952 (24)^b^ | 1,157 (22) | 952 (37)^a^ |
| Primary Insurance, n (%) |  |  |  |  |  |
| Private | 3,935 (23) | 1,330 (27)^a,b,c^ | 917 (23)^b^ | 1,211 (23) | 477 (19)^a^ |
| Medicare | 7,845 (47) | 2,213 (45)^a,b^ | 1,704 (42)^a,b^ | 2,636 (50) | 1,292 (50) |
| Medicaid | 3,592 (21) | 1,045 (21)^a,c^ | 1,036 (26)^a,b^ | 978 (18) | 533 (21) |
| Uninsured | 1,473 (9) | 382 (8)^b^ | 359 (9) | 463 (9) | 269 (10) |
| Residency area characteristics |  |  |  |  |  |
| Total Proportion of African-American (%), mean (SD) | 18.7 (17.4) | 17.3 (15.9)^b,c^ | 19.6 (18.1)^a,b^ | 18.2 (17.0) | 21.3 (19.6)^a^ |
| Population Proportion Below Poverty (%), mean (SD) | 22.5 (10.2) | 21.7 (10.1)^b,c^ | 23.1 (10.3)^a,b^ | 22.0 (10.0) | 23.8 (10.4)^a^ |
| distance from Residency to Hospital (mile), median (IQR) | 18 (3, 34) | 22 (3, 36)^a,b,c^ | 14 (3, 30)^a,b^ | 18 (3, 34) | 14 (3, 27)^a^ |
| **Comorbidities** |  |  |  |  |  |
| Hypertension, n (%) | 8,468 (50) | 2,494 (50) | 1,993 (50) | 2,702 (51) | 1,279 (50) |
| Cardiovascular disease, n (%)^d^ | 4,702 (28) | 1,375 (28) | 1,132 (28) | 1,488 (28) | 707 (27) |
| Diabetes mellitus, n (%) | 3,945 (23) | 1,116 (22) | 941 (23) | 1,262 (24) | 626 (24) |
| Chronic kidney disease, n (%) | 2,892 (17) | 690 (14)^a,b,c^ | 744 (19)^b^ | 880 (17) | 578 (22)^a^ |
| **Admission characteristics of patients** |  |  |  |  |  |
| Emergent Admission, n (%) | 12,289 (73) | 2,889 (58)^a,b,c^ | 3,381 (84)^a,b^ | 3,734 (71) | 2,285 (89)^a^ |
| Transfer from another hospital, n (%) | 2,859 (17) | 738 (15)^b,c^ | 797 (20)^a^ | 859 (16) | 465 (18) |
| **Primary admission diagnostic groups** |  |  |  |  |  |
| Diseases of the circulatory system, n (%) | 2,983 (18) | 800 (16)^a,b,c^ | 554 (14)^a,b^ | 1,034 (20) | 595 (23)^a^ |
| Respiratory and infectious diseases, n (%) | 1,525 (9) | 295 (6)^b,c^ | 630 (16)^a,b^ | 309 (6) | 291 (11)^a^ |
| Complications of pregnancy and childbirth, n (%) | 1,246 (7) | 336 (7)^b,c^ | 425 (11)^a,b^ | 356 (7) | 129 (5)^a^ |
| Diseases of the digestive/genitourinary systems, n (%) | 2,147 (13) | 780 (16)^a,b,c^ | 381 (9)^a^ | 705 (13) | 281 (11)^a^ |
| Diseases of the musculoskeletal/connective tissue and skin, n (%) | 1,418 (8) | 542 (11)^b,c^ | 189 (5)^a,b^ | 518 (10) | 169 (7)^a^ |
| Neoplasms, n (%) | 1,074 (6) | 456 (9)^a,b,c^ | 154 (4)^a^ | 384 (7) | 80 (3)^a^ |
| **Clinical biomarkers and interventions within 24 hours of admission** |  |  |  |  |  |
| Surgery on admission day, n (%) | 3,551 (21) | 1,761 (35)^a,b,c^ | 332 (8)^a^ | 1,251 (24) | 207 (8)^a^ |
| ICU/IMC admission within first 24 hours, n (%) | 3,838 (23) | 1,113 (22)^a,c^ | 1,269 (32)^a,b^ | 880 (17) | 576 (22)^a^ |
| **Cardiovascular system** |  |  |  |  |  |
| Hypotension (MAP < 60 mmHg) at any time, n (%) | 5,916 (35) | 2,917 (59)^a,b,c^ | 1,429 (36)^a,b^ | 1,299 (25) | 271 (11)^a^ |
| Duration, median (IQR), minutes | 60 (15, 167) | 63 (19, 207)^a,b,c^ | 75 (30, 210)^a,b^ | 21 (6, 68) | 30 (6, 90) |
| Vasopressors used, n (%) | 3,166 (19) | 1,631 (33)^a,b,c^ | 437 (11)^a,b^ | 937 (18) | 161 (6)^a^ |
| Out of operating room | 568 (3) | 259 (5)^a,b^ | 205 (5)^a,b^ | 79 (1) | 25 (1) |
| Hypertension (SBP > 160 mmHg) at any time, n (%) | 6,207 (37) | 1,141 (23)^a,b,c^ | 668 (17)^a,b^ | 2,336 (44) | 2,062 (80)^a^ |
| Troponin, tested, n (%) | 5,905 (35) | 1,215 (24)^a,b,c^ | 1,734 (43)^a,b^ | 1,682 (32) | 1,274 (50)^a^ |
| Abnormal result among tested, n (%) | 1,205 (20) | 265 (22)^a^ | 371 (21)^a^ | 265 (16) | 304 (24)^a^ |
| **Respiratory system** |  |  |  |  |  |
| Highest administered FiO2, median (IQR) | 0.21 (0.21, 0.40) | 0.21 (0.21, 0.40)^a,b,c^ | 0.21 (0.21, 0.33)^b^ | 0.21 (0.21, 0.40) | 0.21 (0.21, 0.29)^a^ |
| Room air only, n (%) | 9,682 (57) | 2,509 (50)^a,b,c^ | 2,263 (56)^a,b^ | 3,240 (61) | 1,670 (65)^a^ |
| 0.22 - 0.40, n (%) | 6,082 (36) | 2,100 (42)^a,b,c^ | 1,400 (35)^a^ | 1,819 (34) | 763 (30)^a^ |
| > 0.4, n (%) | 1,081 (6) | 361 (7)^a,b^ | 353 (9)^a,b^ | 229 (4) | 138 (5) |
| PaO2/FiO2, tested with arterial blood gas, n (%) | 2,603 (15) | 812 (16)^a,b,c^ | 891 (22)^a,b^ | 559 (11) | 341 (13)^a^ |
| <200 among tested, n (%) | 964 (37) | 304 (37)^a^ | 374 (42)^a^ | 161 (29) | 125 (37) |
| Mechanical ventilation, n (%) | 918 (5) | 336 (7)^a,b^ | 274 (7)^a,b^ | 198 (4) | 110 (4) |
| **Kidney and acid-base status** |  |  |  |  |  |
| Preadmission estimated glomerular filtration rate^e^ (mL/min per 1.73 m2), median (IQR) | 94 (76, 110) | 97 (80, 113)^a,b^ | 98 (80, 115)^a,b^ | 92 (76, 107) | 89 (56, 104)^a^ |
| Highest / reference creatinine^e^ ratio, mean (SD) | 1.23 (0.95) | 1.25 (1.48)^a,c^ | 1.31 (0.79)^a,b^ | 1.16 (0.51) | 1.22 (0.58)^a^ |
| Renal replacement therapy, n (%) | 268 (2) | 63 (1)^b^ | 57 (1)^b^ | 50 (1) | 98 (4)^s^ |
| Highest Anion Gap, median (IQR), mmol/L | 14 (12, 17) | 14 (12, 16)^a,b,c^ | 15 (13, 18)^a,b^ | 14 (12, 16) | 15 (13, 18)^a^ |
| Arterial Blood Gas tested, n (%) | 2,606 (15) | 812 (16)^a,b,c^ | 892 (22)^a,b^ | 560 (11) | 342 (13)^a^ |
| pH < 7.3 among tested, n (%) | 586 (22) | 213 (26)^a,b^ | 217 (24)^a,b^ | 100 (18) | 56 (16) |
| Highest Base deficit, mean (SD), mmol/L | 4.6 (4.8) | 4.2 (4.3)^c^ | 6.3 (6.0)^a,b^ | 3.3 (3.0) | 3.7 (3.7) |
| Lactate, tested, n (%) | 6,142 (36) | 1,657 (33)^a,b,c^ | 1,980 (49)^a,b^ | 1,534 (29) | 971 (38)^a^ |
| 2 - 4 mmol/L among tested, n (%) | 1,480 (24) | 365 (22)^c^ | 551 (28)^a^ | 332 (22) | 232 (24) |
| > 4 mmol/L among tested, n (%) | 580 (9) | 161 (10)^a,c^ | 279 (14)^a,b^ | 64 (4) | 76 (8)^a^ |
| **Inflammation** |  |  |  |  |  |
| Highest White blood cell count, median (IQR), x10^9/L | 9 (7, 12) | 9 (7, 12)^c^ | 10 (7, 14)^a,b^ | 9 (7, 12) | 9 (7, 12) |
| Highest Premature neutrophils (bands)), median (IQR), % | 9 (4, 18) | 9 (4, 17)^a,b^ | 11 (5, 21)^a,b^ | 6 (2, 12) | 5 (2, 13) |
| Lowest Lymphocytes, median (IQR), % | 16 (9, 24) | 16 (9, 26)^a,c^ | 12 (6, 20)^a,b^ | 18 (11, 26) | 16 (9, 24)^a^ |
| C-reactive protein, tested, n (%) | 2,255 (13) | 542 (11)^b,c^ | 757 (19)^a,b^ | 593 (11) | 363 (14)^a^ |
| Highest C-reactive protein, median (IQR), mg/L | 28 (5, 93) | 17 (4, 88)^a,c^ | 63 (12, 147)^a,b^ | 12 (4, 52) | 18 (4, 67) |
| Erythrocyte sedimentation rate, tested, n (%) | 1,263 (7) | 320 (6)^c^ | 370 (9)^a^ | 370 (7) | 203 (8) |
| Highest Erythrocyte sedimentation rate, median (IQR), mm/h | 41 (20, 73) | 33 (15, 65)^b,c^ | 47 (24, 77)^a^ | 37 (17, 67) | 48 (26, 84)^a^ |
| Highest Temperature, mean (SD), celsius | 37.7 (0.6) | 37.7 (0.5)^a,b,c^ | 37.9 (0.7)^a,b^ | 37.7 (0.5) | 37.7 (0.5) |
| 38 - 39, n (%) | 3,578 (21) | 1,134 (23)^a,b^ | 974 (24)^a,b^ | 993 (19) | 477 (19) |
| > 39, n (%) | 627 (4) | 136 (3)^a,c^ | 342 (9)^a,b^ | 82 (2) | 67 (3)^a^ |
| Lowest Temperature, mean (SD), celsius | 36.8 (0.9) | 36.6 (1.2)^a,b,c^ | 36.8 (0.8)^a^ | 36.8 (0.8) | 36.8 (0.7)^a^ |
| **Hematologic** |  |  |  |  |  |
| Lowest Hemoglobin, mean (SD), g/dL | 11.2 (2.3) | 10.8 (2.2)^a,b^ | 10.9 (2.4)^a,b^ | 11.7 (2.1) | 11.8 (2.2) |
| Highest RDW, mean (SD), % | 15.3 (2.1) | 15.3 (2.1)^a,c^ | 15.6 (2.3)^a,b^ | 15.0 (1.8) | 15.3 (1.9)^a^ |
| Lowest Platelets, median (IQR), x10^9/L | 207 (160, 266) | 199 (154, 256)^a,b,c^ | 210 (158, 278) | 208 (164, 264) | 215 (168, 273)^a^ |
| Platelets < 200, n (%) | 6,882 (41) | 2,158 (43)^a,b,c^ | 1658 (41)^a^ | 2,077 (39) | 989 (38)^a^ |
| < 100 | 1,008 (15) | 340 (16)^a,b,c^ | 342 (21)^a,b^ | 218 (10) | 108 (11) |
| 100 - 200 | 5,874 (85) | 1,818 (84)^a,b,c^ | 1,316 (79)^a,b^ | 1,859 (90) | 881 (89) |
| International normalized ratio, tested, n (%) | 7,070 (42) | 1,927 (39)^b,c^ | 1,823 (45)^a^ | 2,134 (40) | 1,186 (46)^a^ |
| >= 2 | 698 (10) | 208 (11)^a,b^ | 243 (13)^a,b^ | 161 (8) | 86 (7) |
| **Neurologic** |  |  |  |  |  |
| Glasgow Coma Scale score, n (%) |  |  |  |  |  |
| Moderate (9 - 12) | 730 (4) | 252 (5)^a^ | 187 (5) | 191 (4) | 100 (4) |
| Severe (<= 8) | 593 (4) | 187 (4)^a^ | 189 (5)^a^ | 130 (2) | 87 (3) |
| **Liver and metabolic** |  |  |  |  |  |
| Bilirubin, tested, n (%) | 7,931 (47) | 1,954 (39)^a,b,c^ | 2,305 (57)^a^ | 2240 (42) | 1,432 (56)^a^ |
| >= 2 mg/dL, n (%) | 496 (6) | 165 (8)^a,b^ | 186 (8)^a,b^ | 94 (4) | 51 (4) |
| Highest Glucose, median (IQR), mg/dL | 128 (104, 172) | 124 (102, 162)^a,b,c^ | 131 (107, 179)^a^ | 125 (103, 169) | 135 (108, 189)^a^ |
| Albumin, tested, n (%) | 8,023 (48) | 1,979 (40)^a,b,c^ | 2,326 (58)^a^ | 2,274 (43) | 1,444 (56)^a^ |
| < 2.5 | 445 (6) | 139 (7)^a,b^ | 215 (9)^a,b^ | 54 (2) | 37 (3) |
| 2.5 - 3.5 | 2,549 (32) | 699 (35)^a,b^ | 861 (37)^a,b^ | 579 (25) | 410 (28) |

Abbreviation: ICU: intensive care unit; IMC: intermediate care unit; MAP: mean aterial pressure; RDW: red cell distribution width; SD: standard deviation; IQR: interquartile range.

All p-values were adjusted for multiple comparisons using Bonferroni method.

^a^ p < 0.05 compared to Physiotype C .

^b^ p < 0.05 compared to Physiotype D.

^c^ p < 0.05 compared to Physiotype B.

^d^ Cardiovascular disease was considered if there was a history of congestive heart failure, coronary artery disease of peripheral vascular disease.

^e^ Reference glomerular filtration rate and reference creatinine were derived without use of race correction (see S1 Text for details).
