# Supplementary material for: Physiologic signatures within six hours of hospitalization identify acute illness phenotypes
Source: PLOS Digit Health. 2022 Oct 13;1(10):e0000110. doi: 10.1371/journal.pdig.0000110 (PMC9802629; doi:10.1371/journal.pdig.0000110)
Supplement: S17 Table — (DOCX) [file pdig.0000110.s048.docx]

# S17 Table. Physiotype illness severity, clinical outcomes, and resource use in the testing cohort

| **Variables** | **Total** | **Acute Illness Physiotypes** | | | |
| --- | --- | --- | --- | --- | --- |
|  |  | Physiotype A | Physiotype B | Physiotype C | Physiotype D |
| Number of Encounters (%) | 16,845 | 4,970 (30) | 4,016 (24) | 5,288 (31) | 2,571 (15) |
| **Acuity scores within 24h of admission** |  |  |  |  |  |
| SOFA score > 6, n (%) | 1,454 (9) | 569 (11)^a,b^ | 424 (11)^a,b^ | 311 (6) | 150 (6) |
| Patients in ICU/IMC, SOFA score <= 6, n (%) | 2,800 (17) | 724 (15)^b,c^ | 925 (23)^a,b^ | 683 (13) | 468 (18)^a^ |
| Patients in ICU/IMC, SOFA score > 6, n (%) | 1,038 (6) | 389 (8)^a,b^ | 344 (9)^a,b^ | 197 (4) | 108 (4) |
| Patients in ward, SOFA score <= 6, n (%) | 12,591 (75) | 3,677 (74)^a,c^ | 2,667 (66)^a,b^ | 4,294 (81) | 1,953 (76)^a^ |
| Patients in ward, SOFA score > 6, n (%) | 416 (2) | 180 (4)^a,b,c^ | 80 (2) | 114 (2) | 42 (2) |
| MEWS score > 4, n (%) | 1,090 (6) | 156 (3)^a,b,c^ | 601 (15)^a,b^ | 95 (2) | 238 (9)^a^ |
| Patients in ICU/IMC, MEWS score <= 4, n (%) | 3,040 (18) | 982 (20)^a,b^ | 828 (21)^a,b^ | 806 (15) | 424 (16) |
| Patients in ICU/IMC, MEWS score > 4, n (%) | 798 (5) | 131 (3)^a,b,c^ | 441 (11)^a,b^ | 74 (1) | 152 (6)^a^ |
| Patients in ward, MEWS score <= 4, n (%) | 12,715 (75) | 3,832 (77)^a,b,c^ | 2,587 (64)^a,b^ | 4,387 (83) | 1,909 (74)^a^ |
| Patients in ward, MEWS score > 4, n (%) | 292 (2) | 25 (1)^b,c^ | 160 (4)^a^ | 21 (0) | 86 (3)^a^ |
| **Resource use during hospitalization** |  |  |  |  |  |
| Hospital days, median (IQR) | 4 (2, 7) | 4 (2, 6)^a,c^ | 4 (3, 8)^a,b^ | 3 (2, 6) | 4 (2, 7)^a^ |
| Surgery at any time, n (%) | 4,718 (28) | 2,027 (41)^a,b,c^ | 640 (16)^a^ | 1,664 (31) | 387 (15)^a^ |
| Admitted to ICU/IMC^d^, n (%) | 4,616 (27) | 1,291 (26)^a,c^ | 1,535 (38)^a,b^ | 1,094 (21) | 696 (27)^a^ |
| Days in ICU/IMC^e^, median (IQR) | 4 (2, 7) | 4 (2, 7)^a,b^ | 4 (3, 8)^a,b^ | 4 (2, 6) | 3 (2, 6) |
| Days in ICU/IMC greater than 48 hrs, n (%) | 3,401 (74) | 965 (75) | 1,172 (76)^a,b^ | 778 (71) | 486 (70) |
| Mechanical Ventilation, n (%) | 1,349 (8) | 453 (9)^a,b,c^ | 444 (11)^a,b^ | 297 (6) | 155 (6) |
| Mechanical Ventilation hours, median (IQR)^f^ | 28 (10, 92) | 19 (7, 60)^b,c^ | 45 (16, 127)^a^ | 22 (8, 62) | 46 (17, 134)^a^ |
| Mechanical Ventilation greater than 2 calendar days, n (%) | 633 (47) | 163 (36)^b,c^ | 265 (60)^a^ | 115 (39) | 90 (58)^a^ |
| Renal replacement therapy, n (%) | 530 (3) | 124 (2)^b,c^ | 150 (4)^a,b^ | 100 (2) | 156 (6)^a^ |
| **Complications** |  |  |  |  |  |
| Acute kidney injury overall, n (%) | 2,741 (16) | 737 (15)^a,b,c^ | 848 (21)^a^ | 670 (13) | 486 (19)^a^ |
| Community-acquired AKI, n (%) | 1,565 (57) | 457 (62)^a,b^ | 513 (60)^b^ | 368 (55) | 227 (47)^a^ |
| Hospital-acquired AKI, n (%) | 1,176 (43) | 280 (38)^a,b^ | 335 (40)^b^ | 302 (45) | 259 (53)^a^ |
| Worst AKI staging, n (%) |  |  |  |  |  |
| Stage 1 | 1,798 (66) | 472 (64)^a^ | 502 (59)^a,b^ | 489 (73) | 335 (69) |
| Stage 2 | 512 (19) | 146 (20) | 183 (22)^a^ | 98 (15) | 85 (17) |
| Stage 3 | 300 (11) | 83 (11) | 109 (13) | 60 (9) | 48 (10) |
| Stage 3 with RRT | 131 (5) | 36 (5) | 54 (6) | 23 (3) | 18 (4) |
| Venous Thromboembolism, n (%) | 937 (6) | 227 (5)^b,c^ | 330 (8)^a,b^ | 223 (4) | 157 (6)^a^ |
| Sepsis, n (%) | 1,913 (11) | 433 (9)^a,c^ | 996 (25)^a,b^ | 246 (5) | 238 (9)^a^ |
| Hospital disposition, n (%) |  |  |  |  |  |
| Hospital mortality | 513 (3) | 130 (3)^a,c^ | 239 (6)^a,b^ | 88 (2) | 56 (2) |
| Another hospital, LTAC, SNF, Hospice | 1,946 (12) | 535 (11)^c^ | 562 (14)^a^ | 529 (10) | 320 (12)^a^ |
| Home or short-term rehabilitation | 14,386 (85) | 4,305 (87)^c^ | 3,215 (80)^a,b^ | 4,671 (88) | 2,195 (85)^a^ |
| Thirty-day mortality, n (%) | 705 (4) | 178 (4)^a,c^ | 305 (8)^a,b^ | 128 (2) | 94 (4)^a^ |
| Three-year mortality, n (%) | 3,324 (20) | 852 (17)^b,c^ | 1,081 (27)^a,b^ | 839 (16) | 552 (21)^a^ |

Abbreviation: SOFA: sequential organ failure assessment; MEWS: modified early warning score; ICU: intensive care unit; IQR: interquartile range.

All p-values were adjusted for multiple comparisons using Bonferroni method.

^a^ p < 0.05 compared to Physiotype C .

^b^ p < 0.05 compared to Physiotype D.

^c^ p < 0.05 compared to Physiotype B.

^d^ At any time during hospitalization.

^e^ Values were calculated among patients admitted to ICU.

^f^ Values were calculated among patients requiring MV.
