# Supplementary material for: Physiologic signatures within six hours of hospitalization identify acute illness phenotypes
Source: PLOS Digit Health. 2022 Oct 13;1(10):e0000110. doi: 10.1371/journal.pdig.0000110 (PMC9802629; doi:10.1371/journal.pdig.0000110)
Supplement: S18 Table — (DOCX) [file pdig.0000110.s049.docx]

# S18 Table. Statistical output from gaussian mixture modeling in the training cohort (N=41,502)

|  | **Statics** | | **Class size (N = 41,502), n (%)** | | | | | | | |
| --- | --- | --- | --- | --- | --- | --- | --- | --- | --- | --- |
| **Class number** | **BIC** | **Median [IQR] probability of**  **group membership** | **1** | **2** | **3** | **4** | **5** | **6** | **7** | **8** |
| 2 | 4631229 | 100.0 [100.0 – 100.0] | 15,071 (36) | 26,431 (64) | . | . | . | . | . | . |
| 3 | 4777129 | 100.0 [99.5 – 100.0] | 19,467 (47) | 12,005 (29) | 10,030 (24) | . | . | . | . | . |
| 4 | 4851748 | 100.0 [98.0 – 100.0] | 13,508 (33) | 8,929 (22) | 13,777 (33) | 5289 (13) | . | . | . | . |
| 5 | 4909693 | 99.9 [96.9 – 100.0] | 10,287 (25) | 5,768 (14) | 4,818 (12) | 13,341 (32) | 7,288 (18) | . | . | . |
| 6 | 4959853 | 99.9 [96.2 – 100.0] | 7,015 (17) | 7,903 (19) | 10,605 (26) | 3,995 (10) | 2,686 (6) | 9,298 (22) | . | . |
| 7 | 4990147 | 99.8 [94.5 – 100.0] | 9,325 (22) | 6,694 (16) | 6,425 (15) | 2,307 (6) | 3,855 (9) | 3,870 (9) | 9,026 (22) | . |
| 8 | 5002554 | 99.8 [94.6 – 100.0] | 7,569 (18) | 9,022 (22) | 6,962 (17) | 2,460 (6) | 6,236 (15) | 6,596 (16) | 2,626 (6) | 31 (0) |

Abbreviation: BIC: Bayesian information criteria; IQR: interquartile range.
