# Supplementary material for: Physiologic signatures within six hours of hospitalization identify acute illness phenotypes
Source: PLOS Digit Health. 2022 Oct 13;1(10):e0000110. doi: 10.1371/journal.pdig.0000110 (PMC9802629; doi:10.1371/journal.pdig.0000110)
Supplement: S19 Table — (DOCX) [file pdig.0000110.s050.docx]

# S19 Table. Physiotype clinical characteristics and biomarkers by physiotypes derived using gaussian mixture modeling in sensitivity analysis in the training cohort

| **Variables** | **Total** | **Acute Illness Physiotypes** | | | |
| --- | --- | --- | --- | --- | --- |
|  |  | Physiotype A | Physiotype B | Physiotype C | Physiotype D |
| Number of Encounters (%) | 41,502 | 13,508 (33) | 8,929 (22) | 13,777 (33) | 5,288 (13) |
| **Preadmission clinical characteristics** |  |  |  |  |  |
| Age, mean (SD) | 54 (19) | 53 (19)^a,b,c^ | 51 (20)^a,b^ | 56 (18) | 56 (17) |
| Female sex, n (%) | 22,745 (55) | 7,748 (57)^a,b^ | 5,133 (57)^a,b^ | 7,102 (52) | 2,762 (52) |
| Race, n (%) |  |  |  |  |  |
| White | 29,076 (70) | 10,187 (75)^a,b,c^ | 6,178 (69)^b^ | 9,689 (70) | 3,022 (57)^a^ |
| African American | 9,634 (23) | 2,222 (16)^a,b,c^ | 2,178 (24)^b^ | 3,215 (23) | 2,019 (38)^a^ |
| Primary Insurance, n (%) |  |  |  |  |  |
| Private | 9,591 (23) | 3,368 (25)^a,b,c^ | 2,065 (23)^b^ | 3,186 (23) | 972 (18)^a^ |
| Medicare | 18,499 (45) | 5,952 (44)^a,b,c^ | 3,578 (40)^a,b^ | 6,433 (47) | 2,536 (48) |
| Medicaid | 9,231 (22) | 2,960 (22)^a,c^ | 2,368 (27)^a,b^ | 2,772 (20) | 1,131 (21) |
| Uninsured | 4,181 (10) | 1,228 (9)^a,b,c^ | 918 (10)^b^ | 1,386 (10) | 649 (12)^a^ |
| Residency area characteristics |  |  |  |  |  |
| Total Proportion of African-American (%), mean (SD) | 18.7 (17.5) | 17.3 (16.2)^a,b,c^ | 19.3 (17.8)^b^ | 18.7 (17.5) | 21.5 (19.5)^a^ |
| Population Proportion Below Poverty (%), mean (SD) | 22.7 (10.1) | 21.8 (10.0)^a,b,c^ | 23.2 (9.9)^a,b^ | 22.7 (10.1) | 24.1 (10.3)^a^ |
| distance from Residency to Hospital (mile), median (IQR) | 18 (3, 34) | 22 (3, 37)^a,b,c^ | 14 (3, 31)^a,b^ | 18 (3, 34) | 14 (3, 26)^a^ |
| **Comorbidities** |  |  |  |  |  |
| Hypertension, n (%) | 21,639 (52) | 6,917 (51)^b^ | 4,602 (52)^b^ | 7,156 (52) | 2,964 (56)^a^ |
| Cardiovascular disease, n (%)^d^ | 12,058 (29) | 3,729 (28)^a,b^ | 2,604 (29)^b^ | 4,028 (29) | 1,697 (32)^a^ |
| Diabetes mellitus, n (%) | 10,111 (24) | 3,124 (23)^b,c^ | 2,220 (25)^b^ | 3,312 (24) | 1,455 (28)^a^ |
| Chronic kidney disease, n (%) | 6,518 (16) | 1,856 (14)^a,b,c^ | 1,357 (15)^b^ | 2,200 (16) | 1,105 (21)^a^ |
| **Admission characteristics of patients** |  |  |  |  |  |
| Emergent Admission, n (%) | 30,177 (73) | 7,876 (58)^a,b,c^ | 7,540 (84)^a,b^ | 9,996 (73) | 4,765 (90)^a^ |
| Transfer from another hospital, n (%) | 7,115 (17) | 2,066 (15)^b,c^ | 1,845 (21)^a,b^ | 2,238 (16) | 966 (18)^a^ |
| **Primary admission diagnostic groups** |  |  |  |  |  |
| Diseases of the circulatory system, n (%) | 7,719 (19) | 2,288 (17)^a,b^ | 1,406 (16)^a,b^ | 2,654 (19) | 1,371 (26)^a^ |
| Respiratory and infectious diseases, n (%) | 3,306 (8) | 618 (5)^a,b,c^ | 1,337 (15)^a,b^ | 786 (6) | 565 (11)^a^ |
| Complications of pregnancy and childbirth, n (%) | 3,148 (8) | 942 (7)^b,c^ | 955 (11)^a,b^ | 984 (7) | 267 (5)^a^ |
| Diseases of the digestive/genitourinary systems, n (%) | 5,184 (12) | 1,973 (15)^a,b,c^ | 926 (10)^a^ | 1,734 (13) | 551 (10)^a^ |
| Diseases of the musculoskeletal/connective tissue and skin, n (%) | 3,651 (9) | 1,581 (12)^a,b,c^ | 422 (5)^a,b^ | 1,253 (9) | 395 (7)^a^ |
| Neoplasms, n (%) | 2,743 (7) | 1,312 (10)^a,b,c^ | 336 (4)^a,b^ | 968 (7) | 127 (2)^a^ |
| **Clinical biomarkers and interventions within 24 hours of admission** |  |  |  |  |  |
| Surgery on admission day, n (%) | 8,644 (21) | 4,672 (35)^a,b,c^ | 710 (8)^a^ | 2,890 (21) | 372 (7)^a^ |
| ICU/IMC admission within first 24 hours, n (%) | 9,426 (23) | 3,045 (23)^a,c^ | 2,918 (33)^a,b^ | 2,237 (16) | 1,226 (23)^a^ |
| **Cardiovascular system** |  |  |  |  |  |
| Hypotension (MAP < 60 mmHg) at any time, n (%) | 14,470 (35) | 7,733 (57)^a,b,c^ | 3,197 (36)^a,b^ | 3,031 (22) | 509 (10)^a^ |
| Duration, median (IQR), minutes | 57 (15, 168) | 60 (17, 194)^a,b,c^ | 79 (30, 218)^a,b^ | 19 (6, 63) | 26 (8, 66) |
| Vasopressors used, n (%) | 7,531 (18) | 4,263 (32)^a,b,c^ | 927 (10)^a,b^ | 2,054 (15) | 287 (5)^a^ |
| Out of operating room | 1,403 (3) | 661 (5)^a,b^ | 481 (5)^a,b^ | 205 (1) | 56 (1) |
| Hypertension (SBP > 160 mmHg) at any time, n (%) | 14,838 (36) | 2,959 (22)^a,b,c^ | 1,495 (17)^a,b^ | 6,025 (44) | 4,359 (82)^a^ |
| Troponin, tested, n (%) | 14,616 (35) | 3,420 (25)^a,b,c^ | 3,879 (43)^a,b^ | 4,540 (33) | 2,777 (53)^a^ |
| Abnormal result among tested, n (%) | 3,398 (23) | 829 (24)^a^ | 944 (24)^a^ | 891 (20) | 734 (26)^a^ |
| **Respiratory system** |  |  |  |  |  |
| Highest administered FiO2, median (IQR) | 0.21 (0.21, 0.40) | 0.25 (0.21, 0.40)^a,b,c^ | 0.21 (0.21, 0.33)^a,b^ | 0.21 (0.21, 0.37) | 0.21 (0.21, 0.29)^a^ |
| Room air only, n (%) | 23,963 (58) | 6,753 (50)^a,b,c^ | 5,007 (56)^a,b^ | 8,711 (63) | 3,492 (66)^a^ |
| 0.22 - 0.40, n (%) | 14,790 (36) | 5,707 (42)^a,b,c^ | 3,101 (35)^a,b^ | 4,451 (32) | 1,531 (29)^a^ |
| > 0.4, n (%) | 2,749 (7) | 1,048 (8)^a,b,c^ | 821 (9)^a,b^ | 615 (4) | 265 (5) |
| PaO2/FiO2, tested with arterial blood gas, n (%) | 6,113 (15) | 2,115 (16)^a,b,c^ | 1,890 (21)^a,b^ | 1,405 (10) | 703 (13)^a^ |
| <200 among tested, n (%) | 2,265 (37) | 773 (37)^c^ | 807 (43)^a,b^ | 456 (32) | 229 (33) |
| Mechanical ventilation, n (%) | 2,123 (5) | 842 (6)^a,b^ | 632 (7)^a,b^ | 458 (3) | 191 (4) |
| **Kidney and acid-base status** |  |  |  |  |  |
| Preadmission estimated glomerular filtration rate^e^ (mL/min per 1.73 m2), median (IQR) | 95 (78, 111) | 96 (80, 112)^a,b,c^ | 99 (82, 117)^a,b^ | 93 (77, 108) | 90 (57, 105)^a^ |
| Highest / reference creatinine^e^ ratio, mean (SD) | 1.24 (0.66) | 1.24 (0.70)^a,b,c^ | 1.31 (0.73)^a,b^ | 1.19 (0.55) | 1.24 (0.69)^a^ |
| Renal replacement therapy, n (%) | 641 (2) | 173 (1)^b^ | 115 (1)^b^ | 146 (1) | 207 (4)^a^ |
| Highest Anion Gap, median (IQR), mmol/L | 14 (12, 17) | 13 (11, 16)^a,b,c^ | 15 (12, 18)^a,b^ | 14 (12, 16) | 15 (12, 18)^a^ |
| Arterial Blood Gas tested, n (%) | 6,115 (15) | 2,116 (16)^a,b,c^ | 1,891 (21)^a,b^ | 1,405 (10) | 703 (13)^a^ |
| pH < 7.3 among tested, n (%) | 1,437 (23) | 543 (26)^a,b^ | 549 (29)^a,b^ | 227 (16) | 118 (17) |
| Highest Base deficit, mean (SD), mmol/L | 4.8 (4.7) | 4.3 (4.2)^a,c^ | 6.5 (5.9)^a,b^ | 3.6 (3.2) | 4.4 (3.8)^a^ |
| Lactate, tested, n (%) | 15,447 (37) | 4,614 (34)^a,b,c^ | 4,414 (49)^a,b^ | 4,280 (31) | 2,139 (40)^a^ |
| 2 - 4 mmol/L among tested, n (%) | 3,739 (24) | 1,059 (23)^c^ | 1,249 (28)^a,b^ | 929 (22) | 502 (23) |
| > 4 mmol/L among tested, n (%) | 1,374 (9) | 391 (8)^a,c^ | 586 (13)^a,b^ | 232 (5) | 165 (8)^a^ |
| **Inflammation** |  |  |  |  |  |
| Highest White blood cell count, median (IQR), x10^9/L | 9 (7, 13) | 9 (7, 13)^a,b,c^ | 11 (8, 15)^a,b^ | 9 (7, 12) | 9 (7, 12) |
| Highest Premature neutrophils (bands)), median (IQR), % | 10 (4, 20) | 10 (4, 17)^a,c^ | 12 (5, 24)^a,b^ | 6 (2, 14) | 8 (3, 15) |
| Lowest Lymphocytes, median (IQR), % | 16 (9, 24) | 16 (9, 26)^a,c^ | 12 (6, 20)^a,b^ | 18 (11, 26) | 17 (10, 24)^a^ |
| C-reactive protein, tested, n (%) | 5,862 (14) | 1,594 (12)^a,b,c^ | 1,571 (18)^a,b^ | 1,860 (14) | 837 (16)^a^ |
| Highest C-reactive protein, median (IQR), mg/L | 18 (5, 77) | 17 (4, 69)^a,b,c^ | 56 (12, 126)^a,b^ | 12 (3, 57) | 12 (4, 50) |
| Erythrocyte sedimentation rate, tested, n (%) | 3,903 (9) | 1,044 (8)^a,b,c^ | 944 (11) | 1,320 (10) | 595 (11)^a^ |
| Highest Erythrocyte sedimentation rate, median (IQR), mm/h | 40 (19, 73) | 37 (17, 66)^c^ | 51 (24, 88)^a,b^ | 34 (17, 67) | 40 (20, 73) |
| Highest Temperature, mean (SD), celsius | 37.7 (0.6) | 37.7 (0.6)^a,b,c^ | 37.9 (0.8)^a,b^ | 37.6 (0.5) | 37.7 (0.6)^a^ |
| 38 - 39, n (%) | 8,633 (21) | 3,027 (22)^a,b,c^ | 2,210 (25)^a,b^ | 2,387 (17) | 1,009 (19)^a^ |
| > 39, n (%) | 1,548 (4) | 376 (3)^a,c^ | 799 (9)^a,b^ | 232 (2) | 141 (3)^a^ |
| Lowest Temperature, mean (SD), celsius | 36.7 (1.0) | 36.5 (1.3)^a,b,c^ | 36.7 (0.8)^a^ | 36.7 (0.8) | 36.8 (0.7)^a^ |
| **Hematologic** |  |  |  |  |  |
| Lowest Hemoglobin, mean (SD), g/dL | 11.5 (2.3) | 11.1 (2.3)^a,b^ | 11.1 (2.4)^a,b^ | 12.0 (2.2) | 12.0 (2.3) |
| Highest RDW, mean (SD), % | 15.5 (2.1) | 15.5 (2.2)^a,b,c^ | 15.9 (2.3)^a,b^ | 15.3 (1.9) | 15.5 (2.0)^a^ |
| Lowest Platelets, median (IQR), x10^9/L | 210 (161, 269) | 201 (153, 259)^a,b,c^ | 217 (161, 285)^a^ | 212 (167, 266) | 219 (170, 274)^a^ |
| Platelets < 200, n (%) | 16,707 (40) | 5,865 (43)^a,b,c^ | 3,582 (40)^a,b^ | 5,273 (38) | 1,987 (38)^a^ |
| < 100 | 2,643 (16) | 1,020 (17)^a,b,c^ | 747 (21)^a,b^ | 654 (12) | 222 (11) |
| 100 - 200 | 14,064 (84) | 4,845 (83)^a,b,c^ | 2,835 (79)^a,b^ | 4,619 (88) | 1,765 (89) |
| International normalized ratio, tested, n (%) | 20,357 (49) | 5,969 (44)^a,b,c^ | 4,842 (54)^a,b^ | 6,547 (48) | 2,999 (57)^a^ |
| >= 2 | 1,836 (9) | 620 (10)^a,b^ | 548 (11)^a,b^ | 486 (7) | 182 (6) |
| **Neurologic** |  |  |  |  |  |
| Glasgow Coma Scale score, n (%) |  |  |  |  |  |
| Moderate (9 - 12) | 1,708 (4) | 662 (5)^a,b^ | 456 (5)^a,b^ | 418 (3) | 172 (3) |
| Severe (<= 8) | 1,482 (4) | 498 (4)^a,c^ | 461 (5)^a,b^ | 348 (3) | 175 (3)^a^ |
| **Liver and metabolic** |  |  |  |  |  |
| Bilirubin, tested, n (%) | 21,183 (51) | 5,787 (43)^a,b,c^ | 5,490 (61)^a^ | 6,645 (48) | 3,261 (62)^a^ |
| >= 2 mg/dL, n (%) | 1,427 (7) | 559 (10)^a,b^ | 452 (8)^a,b^ | 310 (5) | 106 (3)^a^ |
| Highest Glucose, median (IQR), mg/dL | 126 (104, 170) | 124 (102, 165)^b,c^ | 129 (106, 175)^a,b^ | 124 (103, 167) | 132 (107, 189)^a^ |
| Albumin, tested, n (%) | 21,368 (51) | 5,866 (43)^a,b,c^ | 5,514 (62)^a^ | 6,712 (49) | 3,276 (62)^a^ |
| < 2.5 | 1,243 (6) | 420 (7)^a,b,c^ | 535 (10)^a,b^ | 194 (3) | 94 (3) |
| 2.5 - 3.5 | 6,904 (32) | 2,033 (35)^a,b,c^ | 2,139 (39)^a,b^ | 1,780 (27) | 952 (29)^a^ |

Abbreviation: ICU: intensive care unit; IMC: intermediate care unit; MAP: mean aterial pressure; RDW: red cell distribution width; SD: standard deviation; IQR: interquartile range.

All p-values were adjusted for multiple comparisons using Bonferroni method.

^a^ p < 0.05 compared to Physiotype C .

^b^ p < 0.05 compared to Physiotype D.

^c^ p < 0.05 compared to Physiotype B.

^d^ Cardiovascular disease was considered if there was a history of congestive heart failure, coronary artery disease of peripheral vascular disease.

^e^ Reference glomerular filtration rate and reference creatinine were derived without use of race correction (see S1 Text for details).
