# Supplementary material for: Physiologic signatures within six hours of hospitalization identify acute illness phenotypes
Source: PLOS Digit Health. 2022 Oct 13;1(10):e0000110. doi: 10.1371/journal.pdig.0000110 (PMC9802629; doi:10.1371/journal.pdig.0000110)
Supplement: S20 Table — (DOCX) [file pdig.0000110.s051.docx]

# S20 Table. Physiotype illness severity, clinical outcomes, and resource use by physiotypes derived using gaussian mixture modeling in sensitivity analysis in the training cohort

| **Variables** | **Total** | **Acute Illness Physiotypes** | | | |
| --- | --- | --- | --- | --- | --- |
|  |  | Physiotype A | Physiotype B | Physiotype C | Physiotype D |
| Number of Encounters (%) | 41,502 | 13,508 (33) | 8,929 (22) | 13,777 (33) | 5,288 (13) |
| **Acuity scores within 24h of admission** |  |  |  |  |  |
| SOFA score > 6, n (%) | 3,506 (8) | 1,551 (11)^a,b^ | 941 (11)^a,b^ | 731 (5) | 283 (5) |
| Patients in ICU/IMC, SOFA score <= 6, n (%) | 6,882 (17) | 1,986 (15)^a,b,c^ | 2,114 (24)^a,b^ | 1,770 (13) | 1,012 (19)^a^ |
| Patients in ICU/IMC, SOFA score > 6, n (%) | 2,544 (6) | 1,059 (8)^a,b,c^ | 804 (9)^a,b^ | 467 (3) | 214 (4) |
| Patients in ward, SOFA score <= 6, n (%) | 31,114 (75) | 9,971 (74)^a,c^ | 5,874 (66)^a,b^ | 11,276 (82) | 3,993 (76)^a^ |
| Patients in ward, SOFA score > 6, n (%) | 962 (2) | 492 (4)^a,b,c^ | 137 (2) | 264 (2) | 69 (1)^a^ |
| MEWS score > 4, n (%) | 2,828 (7) | 495 (4)^a,b,c^ | 1,527 (17)^a,b^ | 293 (2) | 513 (10)^a^ |
| Patients in ICU/IMC, MEWS score <= 4, n (%) | 7,235 (17) | 2,637 (20)^a,b^ | 1,700 (19)^a,b^ | 2,012 (15) | 886 (17)^a^ |
| Patients in ICU/IMC, MEWS score > 4, n (%) | 2,191 (5) | 408 (3)^a,b,c^ | 1,218 (14)^a,b^ | 225 (2) | 340 (6)^a^ |
| Patients in ward, MEWS score <= 4, n (%) | 31,439 (76) | 10,376 (77)^a,b,c^ | 5,702 (64)^a,b^ | 11,472 (83) | 3,889 (74)^a^ |
| Patients in ward, MEWS score > 4, n (%) | 637 (2) | 87 (1)^b,c^ | 309 (3)^a^ | 68 (0) | 173 (3)^a^ |
| **Resource use during hospitalization** |  |  |  |  |  |
| Hospital days, median (IQR) | 4 (2, 7) | 4 (2, 6)^a,c^ | 4 (3, 8)^a,b^ | 3 (2, 6) | 4 (2, 7)^a^ |
| Surgery at any time, n (%) | 11,634 (28) | 5,516 (41)^a,b,c^ | 1,363 (15)^a^ | 3,977 (29) | 778 (15)^a^ |
| Admitted to ICU/IMC^d^, n (%) | 11,121 (27) | 3,510 (26)^a,b,c^ | 3,376 (38)^a^ | 2,751 (20) | 1,484 (28)^a^ |
| Days in ICU/IMC^e^, median (IQR) | 4 (2, 7) | 4 (3, 7)^a,b^ | 4 (3, 8)^a,b^ | 4 (2, 7) | 4 (2, 6) |
| Days in ICU/IMC greater than 48 hrs, n (%) | 8,332 (75) | 2,646 (75)^a^ | 2,626 (78)^a^ | 1,957 (71) | 1,103 (74) |
| Mechanical Ventilation, n (%) | 3,218 (8) | 1,185 (9)^a,b,c^ | 988 (11)^a,b^ | 755 (5) | 290 (5) |
| Mechanical Ventilation hours, median (IQR)^f^ | 35 (14, 113) | 24 (11, 83)^b,c^ | 48 (17, 143)^a^ | 31 (13, 110) | 53 (20, 148)^a^ |
| Mechanical Ventilation greater than 2 calendar days, n (%) | 1,661 (52) | 520 (44)^b,c^ | 588 (60)^a^ | 373 (49) | 180 (62)^a^ |
| Renal replacement therapy, n (%) | 1,262 (3) | 343 (3)^b,c^ | 288 (3)^a,b^ | 298 (2) | 333 (6)^a^ |
| **Complications** |  |  |  |  |  |
| Acute kidney injury overall, n (%) | 6,905 (17) | 2,089 (15)^a,b,c^ | 2,011 (23)^a,b^ | 1,804 (13) | 1,001 (19)^a^ |
| Community-acquired AKI, n (%) | 3,839 (56) | 1,299 (62)^a,b,c^ | 1,165 (58)^a,b^ | 935 (52) | 440 (44)^a^ |
| Hospital-acquired AKI, n (%) | 3,066 (44) | 790 (38)^a,b,c^ | 846 (42)^a,b^ | 869 (48) | 561 (56)^a^ |
| Worst AKI staging, n (%) |  |  |  |  |  |
| Stage 1 | 4,360 (63) | 1,274 (61)^a,b^ | 1,171 (58)^a,b^ | 1,250 (69) | 665 (66) |
| Stage 2 | 1,362 (20) | 435 (21)^a,b^ | 463 (23)^a,b^ | 297 (16) | 167 (17) |
| Stage 3 | 848 (12) | 274 (13) | 264 (13) | 190 (11) | 120 (12) |
| Stage 3 with RRT | 335 (5) | 106 (5) | 113 (6)^a^ | 67 (4) | 49 (5) |
| Venous Thromboembolism, n (%) | 1,257 (3) | 357 (3)^c^ | 360 (4)^a,b^ | 387 (3) | 153 (3) |
| Sepsis, n (%) | 3,750 (9) | 941 (7)^a,c^ | 1,881 (21)^a,b^ | 561 (4) | 367 (7)^a^ |
| Hospital disposition, n (%) |  |  |  |  |  |
| Hospital mortality | 1,141 (3) | 305 (2)^a,c^ | 487 (5)^a,b^ | 241 (2) | 108 (2) |
| Another hospital, LTAC, SNF, Hospice | 4,475 (11) | 1,357 (10)^b,c^ | 1,157 (13)^a^ | 1,330 (10) | 631 (12)^a^ |
| Home or short-term rehabilitation | 35,886 (86) | 11,846 (88)^b,c^ | 7,285 (82)^a,b^ | 12,206 (89) | 4,549 (86)^a^ |
| Thirty-day mortality, n (%) | 1,633 (4) | 448 (3)^a,c^ | 666 (7)^a,b^ | 352 (3) | 167 (3) |
| Three-year mortality, n (%) | 8,013 (19) | 2,325 (17)^b,c^ | 2,341 (26)^a,b^ | 2,261 (16) | 1,086 (21)^a^ |

Abbreviation: SOFA: sequential organ failure assessment; MEWS: modified early warning score; ICU: intensive care unit; IMC: intermediate care unit; IQR: interquartile range.

All p-values were adjusted for multiple comparisons using Bonferroni method.

^a^ p < 0.05 compared to Physiotype C .

^b^ p < 0.05 compared to Physiotype D.

^c^ p < 0.05 compared to Physiotype B.

^d^ At any time during hospitalization.

^e^ Values were calculated among patients admitted to ICU/IMC.

^f^ Values were calculated among patients requiring MV.
